# The Metacarpophalangeal Pattern Profile: An Old Method With New Insights Into the Evaluation of Short Stature

**DOI:** 10.1002/ajhb.70212

**Published:** 2026-02-02

**Authors:** Marcelo Damaso Maruichi, Bruno Telma Destailleur, Giulia Maesta Apelbaum, Carlos Alberto Longui, Cristiane Kochi

**Affiliations:** ^1^ Faculdade de Ciencias Medicas da Santa Casa de Sao Paulo São Paulo Brazil

**Keywords:** metacarpophalangeal pattern profile, short stature, skeletal dysplasias

## Abstract

**Objective:**

To characterize the metacarpophalangeal pattern profile (MCPP) of healthy children and adolescents from São Paulo, Brazil, and to establish percentile curves by chronological age (CA), bone age (BA), and sex using the LMS method. Additionally, to compare these findings with previous population‐based data and to apply the derived standards to patients with skeletal dysplasias.

**Methods:**

Left hand and wrist radiographs were obtained from healthy individuals and age‐matched patients with confirmed skeletal dysplasias. Tubular bone lengths were compared across CA and BA, against prior normative studies, and with dysplastic cohorts using Student's *t*‐test. Patient Z‐scores were calculated from LMS parameters generated from the healthy population.

**Results:**

We analyzed 974 radiographs from healthy subjects and 83 from patients (18 hypochondroplasia, 27 achondroplasia, 14 osteogenesis imperfecta, 24 Turner syndrome). In healthy participants, hand bone lengths correlated significantly with CA and BA. Compared with international reference data, differences in mean metacarpal and phalangeal lengths were noted. Patients with achondroplasia and hypochondroplasia exhibited markedly reduced Z‐scores relative to controls, whereas those with Turner syndrome showed reductions of up to 1.8 SD in the fourth metacarpal. Patients with osteogenesis imperfecta demonstrated no significant deviations.

**Conclusion:**

This study established MCPP reference percentiles for Brazilian children and adolescents using the LMS method. Bone measurements showed consistent associations with CA and BA. Although differences were observed relative to international cohorts, the generated standards effectively discriminated dysplastic phenotypes, particularly achondroplasia and hypochondroplasia, supporting the use of MCPP analysis as an adjunct tool for evaluating short stature and suspected skeletal dysplasias.

## 1 Introduction

Metacarpophalangeal pattern profile (MCPP) analysis is a method for representing the lengths of the metacarpals and phalanges of the hand according to age and sex (Garn et al. 1972). In this technique, the tubular bones of the hand are measured at their maximum length and then the extent to which each of these bones deviates from the mean reference values for the patient's age and sex is determined. This dispersion around the mean is given in units of standard deviations (SD) or converted into Z scores (Garn et al. 1972).

Bone dysplasias are a heterogeneous group of diseases that present a wide range of clinical phenotypes, ranging from subtle bone deformities to severe forms, which can significantly compromise the quality of life of patients (Archibald et al. 1959; Butler et al. 1986; Panda et al. 2014; Poznanski and Gartman 1997). Many severe malformations can be diagnosed in the perinatal period (Andersen and Hauge 1989). Nonetheless, bone dysplasias with milder phenotypes can easily go unnoticed, delaying the diagnosis and treatment of these patients, thus resulting in a worse prognosis. These dysplasias can progress to disproportionate short stature or other physical deformities, many of them leading to changes in the length of the long bones of the hand (Andersen and Hauge 1989; Butler et al. 1986; Fendel 1976; Garn et al. 1972).

Many morphostructural alterations of the hand characteristic of various diseases have already been described, as exemplified in Table 1. Therefore, assessing phalangeal and metacarpal length measurements in healthy individuals is important for establishing reference values to support the evaluation of potential cases of skeletal dysplasia.

**TABLE 1:** Description of morphostructural alterations of hand bones by disease.

Disorder	Inheritance	Gene(s) involved	Hand bone abnormalities	Author/Year of classical description
Holt‐Oram syndrome	AD	*TBX5*	Radial ray defects, shortened/absent thumbs, carpal anomalies	Holt & Oram, 1960
Turner syndrome	Chromosomal (45, X)	—	Shortening of the 4th metacarpal, brachydactyly; mild clinodactyly	Turner, 1938
Pseudohypoparathyroidism (Albright's hereditary osteodystrophy)	AD	*GNAS*	Brachydactyly (short. 4th/5th metacarpals); short phalanges; cone‐shaped epiphyses	Albright, 1942
Robinow syndrome	AR/AD	*ROR2, WNT5A*	Brachydactyly; broad thumbs; mesomelic limb shortening; hypoplastic distal phalanges	Robinow, 1969
Diastrophic dysplasia	AR	*SLC26A2*	shortened metacarpals/phalanges	Lamy & Maroteaux, 1960
Acrodysostosis	AD	*PRKAR1A, PDE4D*	Severe shortening of metacarpals and phalanges	Maroteaux & Malamut, 1968
Rett syndrome	X‐linked dominant (de novo)	*MECP2*	Small hands, short metacarpals and phalanges	Rett, 1966
Marfan syndrome	AD	*FBN1*	Arachnodactyly (long metacarpals and phalanges)	Marfan, 1896
Prader‐Willi syndrome	Chromosomal (15q11–q13)	—	Small hands (acromicria), short 1st metacarpal	Prader, 1956
Gorlin‐Goltz syndrome (Basal cell nevus syndrome)	AD	*PTCH1*	Occasionally brachydactyly, shortened metacarpals reported	Gorlin & Goltz, 1960
Apert syndrome	AD	*FGFR2*	Syndactyly with shortened/broad phalanges	Apert, 1906
Campomelic dysplasia	AD (mostly de novo)	*SOX9*	Shortened metacarpals and phalanges	Maroteaux, 1971
Ellis‐van Creveld disease	AR	*EVC, EVC2*	Short metacarpals and phalanges	Ellis & van Creveld, 1940
Roberts syndrome	AR	*ESCO2*	Severe shortening/absence of phalanges/metacarpals	Roberts, 1919
Ehlers‐Danlos syndrome (subtypes)	AD/AR	*COL5A1, COL3A1*	Long slender fingers (mild arachnodactyly)	Ehlers, 1901; Danlos, 1908
Langer‐Giedion syndrome	AD (8q24 deletion)	*EXT1, TRPS1*	Shortened phalanges, cone‐shaped epiphyses	Langer & Giedion, 1969
Achondroplasia	AD (mostly de novo)	*FGFR3*	Shortening of metacarpals/phalanges, “trident hand”	Parrot, 1878
Brachydactyly type E	AD	*PTHLH, HOXD13*	Shortening of 4th/5th metacarpals	Bell, 1951
Cleidocranial dysplasia	AD	*RUNX2*	Shortened metacarpals/phalanges	Marie & Sainton, 1898
Léri‐Weill dyschondrosteosis	Pseudo‐AD (SHOX haploinsufficiency)	*SHOX*	Short metacarpals, Madelung deformity	Léri & Weill, 1929
Langer syndrome (homozygous SHOX deficiency)	AR	*SHOX*	Severe micromelia and short metacarpals and phalanges	Langer, 1965
Meier‐Gorlin syndrome	AR	*ORC1, ORC4, ORC6, CDT1, CDC6*	Short metacarpals and phalanges	Meier & Gorlin, 1959
Pseudoachondroplasia	AD	*COMP*	Short metacarpals and irregular phalanges	Maroteaux & Lamy, 1959
Spondyloepiphyseal dysplasia congenita	AD	*COL2A1*	Short tubular hand bones	Spranger, 1966
Seckel syndrome	AR	*ATR, RBBP8*, others	Small hands, short phalanges	Seckel, 1960

Abbreviations: AD: autossomal dominant inheritance; AR: autossomal recessive inheritance.

There is a difference in the size of the phalanges and metacarpals according to sex and chronological age (El Morsi and Al Hawary 2013); however, there are no recent studies of healthy children and adolescents to demonstrate the normal values in both genders that include not only chronological age (CA) but also bone age (BA).

Therefore, the aim of this study was to determine the metacarpophalangeal profile of a sample of healthy children and adolescents from the population of the city of São Paulo, Brazil, and to create percentile intervals for the metacarpophalangeal profile according to chronological age (CA), bone age (BA), and sex using the parametric LMS method. In addition, we compared these intervals with previous population‐based studies and we used radiographs of patients with known bone dysplasias to *t*‐test these measurements.

## 2 Material and Methods

This study used left hand and wrist radiographs from a previous study (Artioli et al. 2019) to determine bone age and MCPP in healthy individuals aged 6 to 15 years. In this study, healthy children and adolescents from public and private schools in the city of São Paulo were selected between 2016 and 2018. The participation of children and adolescents was voluntary, without any financial incentive.

For the participants with bone dysplasia, a retrospective analysis of left hand and wrist radiographs was performed to establish MCPP of all children and adolescents who had been referred to the Skeletal Dysplasia Service Clinic at Santa Casa de Misericordia de Sao Paulo Hospital, between 2021 and 2022, with a confirmed diagnosis of Turner syndrome, achondroplasia, hypochondroplasia or osteogenesis imperfecta (OI), the four most prevalent skeletal dysplasias in our department. These radiographs were selected by two medical radiologists with 7 and 8 years of experience in musculoskeletal radiology. All of these patients presented with a height Z‐score below −2 SD.

The participants and their guardians agreed to participate in the study and signed a Free and Informed Assent Form or a Free and Informed Consent Form, respectively. This project was approved by the Human Research Ethics Committee of the Institution (CAAE: 32572120.00000.5479).

Turner syndrome was diagnosed by karyotype (Cui et al. 2018; Laurencikas et al. 2005). Achondroplasia and hypochondroplasia were diagnosed by clinical and imaging examinations and by the presence of a pathogenic variant of the fibroblast growth factor receptor type 3 (*FGFR3*) gene (Bober et al. 1993; Laurencikas et al. 2006; Pauli 2019; Wynn et al. 2007). OI was diagnosed by clinical data and radiological examinations. In addition, patients who presented genetic *t*‐tests indicating the presence of a pathogenic variant in the *COL1A1* or *COL1A2* genes of OI (Renaud et al. 2013; Santili et al. 2005), in addition to compatible clinical and radiological alterations, were also included. The exclusion criteria for both groups were the presence of any evidence of acute fractures or surgeries in the evaluated region or the presence of tumor lesions in fingers or hand bones. Individuals with completely absent ossification/maturation of the epiphyses of the phalanges and metacarpals were also excluded.

In the radiographs of healthy individuals, bone age was assessed by two pediatric endocrinologists with 30 years of experience and one medical radiologist with 10 years of experience in musculoskeletal radiology, using the conventional Greulich Pyle method (Greulich and Pyle 1950). The bone age readers were blind to chronological age.

The radiographic examinations were performed using a mobile X‐ray device (Portable X‐Ray Poskom PXM20BT and CR Carestream Vita, Rochester, NY) using the same protocol as a standard hand and wrist radiograph for bone age assessment. Radiographs were acquired in the DICOM (Digital Imaging and Communications in Medicine) file format and stored in the PACS platform. The measurements on the radiograph were performed using the Radiant program version 2023.1.

**FIGURE 1:**
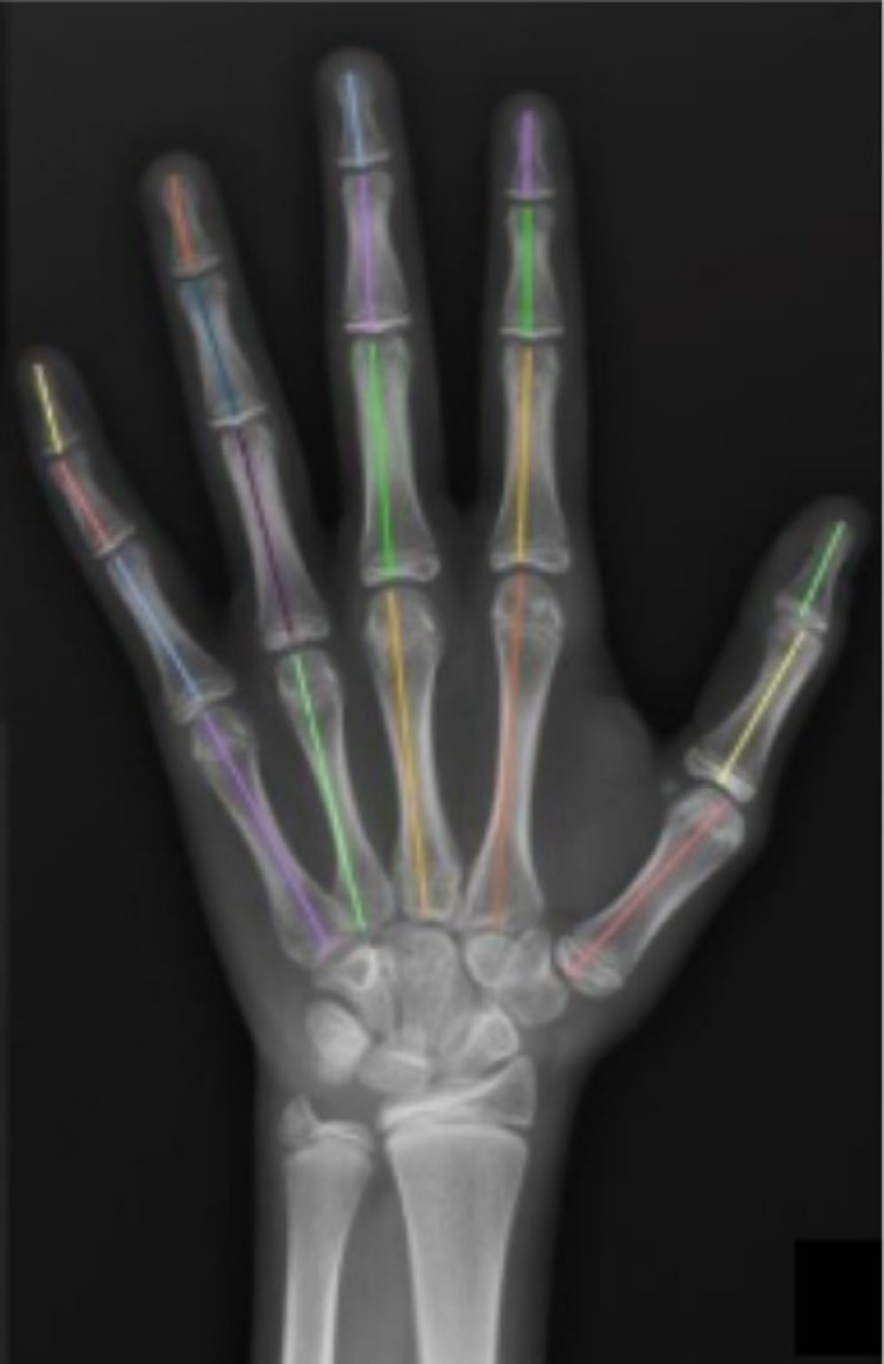
Radiograph of the left hand and wrist of a 13‐year‐old female patient showing the measurements of the length of the phalanges and metacarpals according to the Garn technique (Garn et al. 1972). The colors are for illustrative purposes only.

The proximal and distal phalanges of the thumb; the proximal, middle, and distal phalanges of the second to fifth fingers; and the metacarpals of the first to fifth fingers were measured in centimeters to one decimal place according to the technique described by Garn (Garn et al. 1972) (Figure 1), excluding the styloid process of the base of the third metacarpal.

These measurements were then distributed according to the BA, CA, and sex, with the aim of calculating the mean and standard deviation (SD) of the healthy population. This data was used to create percentile intervals, enabling the evaluation of patients according to the Z score. All of the measurements described above were performed by three medical radiologists with 7, 8, and 10 years of experience in musculoskeletal radiology.

## 3 Statistical Analysis

Exploratory data analysis was performed by calculating summary measures (mean, standard deviation, minimum, median, and maximum). The comparison between the BA and CA of the healthy population was performed using Spearman's correlation coefficient and Student's *t*‐test. The BA and CA in years were used as independent variables. Inter‐observer agreement was assessed using the Intraclass Correlation Coefficient (ICC).

To enable the creation of growth percentiles intervals for each measurement according to sex for the healthy individuals, the Lambda, Mu, and Sigma (LMS) parametric model was used. The Z score of the patients was calculated using the parameters L, M, and S of the normal individuals, following the formula $Z=\frac{{\left(\frac{\mathrm{Vd}}{M}\right)}^{L}-1\;}{L*S}$, where Vd = observed value of the patient, L, M, and S are the parameters of the healthy children of the same sex and chronological age as the patient.

To compare hand bone lengths between the studied population and population‐based studies from the literature, a *t*‐test based on summary data (mean, standard deviation, and sample size of each group) was used. The False Discovery Rate (FDR) method was applied to reduce the risk of identifying differences as statistically significant when they occurred merely by chance.

The significance level adopted was 5%. R software, version 4.2.0 (Copyright 2022, The R Foundation for Statistical Computing) was used for the analysis.

## 4 Results

The group of initially seen individuals comprised 1037 children and adolescents, but five were excluded due to type 1 diabetes mellitus and 31 were excluded due to incomplete data in the anamnesis. Thus, a total of 1001 radiographs were performed, of which 27 were excluded due to trauma sequelae confirmed by clinical history, resulting in a final sample size of 974 children and adolescents, 560 of whom were male (57.5%), with a mean (SD) chronological age of 11 (3.2) years, ranging from 6 to 16 years.

Patients under 6 years of age and over 16 years of age were not included, since only school‐age children and adolescents participated in the study. Furthermore, all epiphyses of the hand bones evaluated were fully or partially ossified in this age group.

The calculation of the mean, standard deviation, LMS parameters, and percentiles for the lengths of the hand metacarpals and phalanges in relation to chronological age is described in Table 2 for females and Table 3 for males.

**TABLE 2:** Age (years), number of individuals (*N*), mean length (cm), standard deviation (SD), LMS parameters and percentiles of the long bones of the hand by chronological age for females.

	Age	N	Mean	S.D.	L	M	S	P5	P10	P25	P50	P75	P90	P95
DP1	6	37	1.50	0.16	0.613	1.517	0.099	1.27	1.32	1.41	1.52	1.62	1.71	1.77
	7	30	1.58	0.16	0.613	1.580	0.099	1.32	1.38	1.47	1.58	1.69	1.78	1.84
	8	43	1.67	0.19	0.613	1.641	0.099	1.37	1.43	1.53	1.64	1.75	1.85	1.91
	9	40	1.69	0.17	0.613	1.698	0.099	1.42	1.48	1.58	1.70	1.81	1.92	1.98
	10	63	1.77	0.17	0.613	1.750	0.099	1.46	1.53	1.63	1.75	1.87	1.97	2.04
	11	53	1.79	0.18	0.613	1.797	0.099	1.50	1.57	1.68	1.80	1.92	2.03	2.09
	12	42	1.83	0.19	0.613	1.841	0.099	1.54	1.60	1.72	1.84	1.96	2.08	2.14
	13	33	1.91	0.17	0.613	1.883	0.099	1.57	1.64	1.76	1.88	2.01	2.12	2.19
	14	24	1.90	0.20	0.613	1.924	0.099	1.61	1.68	1.79	1.92	2.05	2.17	2.24
	15	23	1.97	0.15	0.613	1.966	0.099	1.64	1.71	1.83	1.97	2.10	2.22	2.29
PP1	6	37	2.00	0.16	0.986	1.987	0.090	1.66	1.73	1.85	1.99	2.12	2.24	2.31
	7	30	2.07	0.17	0.986	2.084	0.090	1.74	1.82	1.94	2.08	2.22	2.35	2.43
	8	43	2.17	0.20	0.986	2.189	0.090	1.83	1.91	2.04	2.19	2.34	2.47	2.55
	9	40	2.29	0.21	0.986	2.298	0.090	1.92	2.00	2.14	2.30	2.45	2.59	2.68
	10	63	2.43	0.23	0.986	2.403	0.090	2.01	2.09	2.24	2.40	2.57	2.71	2.80
	11	53	2.51	0.19	0.986	2.492	0.090	2.08	2.17	2.32	2.49	2.66	2.81	2.90
	12	42	2.51	0.28	0.986	2.567	0.090	2.14	2.24	2.39	2.57	2.74	2.90	2.99
	13	33	2.65	0.28	0.986	2.634	0.090	2.20	2.30	2.46	2.63	2.81	2.97	3.07
	14	24	2.73	0.16	0.986	2.696	0.090	2.25	2.35	2.51	2.70	2.88	3.04	3.14
	15	23	2.73	0.25	0.986	2.752	0.090	2.30	2.40	2.57	2.75	2.94	3.10	3.20
MC1	6	37	3.06	0.24	0.787	3.064	0.081	2.56	2.67	2.86	3.06	3.27	3.46	3.57
	7	30	3.21	0.20	0.787	3.241	0.081	2.71	2.83	3.02	3.24	3.46	3.66	3.77
	8	43	3.46	0.36	0.787	3.420	0.081	2.86	2.98	3.19	3.42	3.65	3.86	3.98
	9	40	3.57	0.31	0.787	3.587	0.081	3.00	3.13	3.35	3.59	3.83	4.05	4.18
	10	63	3.76	0.26	0.787	3.735	0.081	3.12	3.26	3.48	3.73	3.99	4.21	4.35
	11	53	3.87	0.29	0.787	3.859	0.081	3.22	3.36	3.60	3.86	4.12	4.35	4.49
	12	42	3.91	0.40	0.787	3.963	0.081	3.31	3.46	3.70	3.96	4.23	4.47	4.62
	13	33	4.09	0.35	0.787	4.054	0.081	3.39	3.53	3.78	4.05	4.33	4.57	4.72
	14	24	4.18	0.21	0.787	4.124	0.081	3.45	3.60	3.85	4.12	4.40	4.65	4.80
	15	23	4.13	0.29	0.787	4.177	0.081	3.49	3.64	3.89	4.18	4.46	4.71	4.86
DP2	6	37	1.09	0.09	0.781	1.089	0.091	0.91	0.95	1.02	1.09	1.16	1.23	1.27
	7	30	1.14	0.10	0.781	1.157	0.091	0.97	1.01	1.08	1.16	1.23	1.30	1.35
	8	43	1.24	0.11	0.781	1.224	0.091	1.02	1.07	1.14	1.22	1.31	1.38	1.43
	9	40	1.31	0.13	0.781	1.283	0.091	1.07	1.12	1.20	1.28	1.37	1.45	1.49
	10	63	1.32	0.12	0.781	1.330	0.091	1.11	1.16	1.24	1.33	1.42	1.50	1.55
	11	53	1.37	0.14	0.781	1.369	0.091	1.14	1.19	1.28	1.37	1.46	1.54	1.59
	12	42	1.40	0.14	0.781	1.405	0.091	1.17	1.23	1.31	1.41	1.50	1.59	1.64
	13	33	1.45	0.11	0.781	1.437	0.091	1.20	1.25	1.34	1.44	1.53	1.62	1.67
	14	24	1.45	0.13	0.781	1.463	0.091	1.22	1.28	1.36	1.46	1.56	1.65	1.70
	15	23	1.49	0.11	0.781	1.486	0.091	1.24	1.30	1.39	1.49	1.59	1.68	1.73
MP2	6	37	1.54	0.12	1.433	1.536	0.089	1.28	1.34	1.43	1.54	1.64	1.73	1.79
	7	30	1.58	0.14	1.433	1.609	0.089	1.34	1.40	1.50	1.61	1.72	1.81	1.87
	8	43	1.69	0.16	1.433	1.684	0.089	1.41	1.47	1.57	1.68	1.80	1.90	1.96
	9	40	1.74	0.17	1.433	1.758	0.089	1.47	1.53	1.64	1.76	1.88	1.98	2.05
	10	63	1.85	0.16	1.433	1.828	0.089	1.53	1.59	1.70	1.83	1.95	2.06	2.13
	11	53	1.88	0.17	1.433	1.889	0.089	1.58	1.65	1.76	1.89	2.02	2.13	2.20
	12	42	1.91	0.21	1.433	1.944	0.089	1.62	1.69	1.81	1.94	2.08	2.19	2.26
	13	33	2.02	0.20	1.433	1.993	0.089	1.67	1.74	1.86	1.99	2.13	2.25	2.32
	14	24	2.02	0.12	1.433	2.038	0.089	1.70	1.78	1.90	2.04	2.18	2.30	2.37
	15	23	2.08	0.16	1.433	2.081	0.089	1.74	1.81	1.94	2.08	2.22	2.35	2.42
PP2	6	37	2.68	0.18	1.339	2.683	0.075	2.24	2.34	2.50	2.68	2.86	3.03	3.12
	7	30	2.83	0.19	1.339	2.822	0.075	2.36	2.46	2.63	2.82	3.01	3.18	3.29
	8	43	2.96	0.22	1.339	2.963	0.075	2.48	2.58	2.76	2.96	3.16	3.34	3.45
	9	40	3.07	0.25	1.339	3.102	0.075	2.59	2.70	2.89	3.10	3.31	3.50	3.61
	10	63	3.26	0.23	1.339	3.229	0.075	2.70	2.82	3.01	3.23	3.45	3.64	3.76
	11	53	3.35	0.26	1.339	3.337	0.075	2.79	2.91	3.11	3.34	3.56	3.77	3.89
	12	42	3.37	0.33	1.339	3.428	0.075	2.86	2.99	3.20	3.43	3.66	3.87	3.99
	13	33	3.53	0.27	1.339	3.509	0.075	2.93	3.06	3.27	3.51	3.75	3.96	4.09
	14	24	3.59	0.18	1.339	3.583	0.075	2.99	3.12	3.34	3.58	3.82	4.04	4.17
	15	23	3.63	0.27	1.339	3.652	0.075	3.05	3.18	3.41	3.65	3.90	4.12	4.25
MC2	6	37	4.61	0.34	1.666	4.631	0.071	3.87	4.04	4.32	4.63	4.94	5.23	5.39
	7	30	4.85	0.32	1.666	4.874	0.071	4.07	4.25	4.55	4.87	5.20	5.50	5.68
	8	43	5.14	0.38	1.666	5.120	0.071	4.28	4.46	4.77	5.12	5.47	5.78	5.96
	9	40	5.28	0.42	1.666	5.358	0.071	4.48	4.67	5.00	5.36	5.72	6.05	6.24
	10	63	5.62	0.36	1.666	5.574	0.071	4.66	4.86	5.20	5.57	5.95	6.29	6.49
	11	53	5.77	0.43	1.666	5.750	0.071	4.80	5.01	5.36	5.75	6.14	6.49	6.70
	12	42	5.75	0.48	1.666	5.892	0.071	4.92	5.14	5.49	5.89	6.29	6.65	6.86
	13	33	6.09	0.46	1.666	6.022	0.071	5.03	5.25	5.62	6.02	6.43	6.79	7.01
	14	24	6.16	0.28	1.666	6.137	0.071	5.13	5.35	5.72	6.14	6.55	6.92	7.15
	15	23	6.21	0.40	1.666	6.239	0.071	5.21	5.44	5.82	6.24	6.66	7.04	7.27
DP3	6	37	1.17	0.11	0.677	1.170	0.098	0.98	1.02	1.09	1.17	1.25	1.32	1.36
	7	30	1.21	0.12	0.677	1.231	0.098	1.03	1.07	1.15	1.23	1.31	1.39	1.43
	8	43	1.30	0.13	0.677	1.293	0.098	1.08	1.13	1.21	1.29	1.38	1.46	1.51
	9	40	1.38	0.15	0.677	1.353	0.098	1.13	1.18	1.26	1.35	1.44	1.53	1.58
	10	63	1.41	0.13	0.677	1.405	0.098	1.17	1.22	1.31	1.40	1.50	1.59	1.64
	11	53	1.44	0.16	0.677	1.450	0.098	1.21	1.26	1.35	1.45	1.55	1.64	1.69
	12	42	1.49	0.17	0.677	1.490	0.098	1.24	1.30	1.39	1.49	1.59	1.68	1.73
	13	33	1.53	0.13	0.677	1.525	0.098	1.27	1.33	1.42	1.52	1.63	1.72	1.78
	14	24	1.57	0.11	0.677	1.557	0.098	1.30	1.36	1.45	1.56	1.66	1.76	1.81
	15	23	1.58	0.14	0.677	1.586	0.098	1.33	1.38	1.48	1.59	1.69	1.79	1.85
MP3	6	37	1.89	0.17	1.263	1.898	0.084	1.59	1.65	1.77	1.90	2.03	2.14	2.21
	7	30	1.99	0.14	1.263	1.983	0.084	1.66	1.73	1.85	1.98	2.12	2.24	2.31
	8	43	2.07	0.16	1.263	2.071	0.084	1.73	1.81	1.93	2.07	2.21	2.34	2.41
	9	40	2.14	0.18	1.263	2.157	0.084	1.80	1.88	2.01	2.16	2.30	2.43	2.51
	10	63	2.26	0.19	1.263	2.239	0.084	1.87	1.95	2.09	2.24	2.39	2.53	2.61
	11	53	2.32	0.22	1.263	2.311	0.084	1.93	2.01	2.16	2.31	2.47	2.61	2.69
	12	42	2.32	0.25	1.263	2.374	0.084	1.98	2.07	2.21	2.37	2.53	2.68	2.76
	13	33	2.46	0.22	1.263	2.431	0.084	2.03	2.12	2.27	2.43	2.59	2.74	2.83
	14	24	2.48	0.13	1.263	2.484	0.084	2.08	2.17	2.32	2.48	2.65	2.80	2.89
	15	23	2.53	0.16	1.263	2.535	0.084	2.12	2.21	2.36	2.53	2.71	2.86	2.95
PP3	6	37	3.01	0.21	1.458	3.023	0.074	2.53	2.64	2.82	3.02	3.23	3.41	3.52
	7	30	3.19	0.22	1.458	3.172	0.074	2.65	2.77	2.96	3.17	3.39	3.58	3.69
	8	43	3.32	0.23	1.458	3.323	0.074	2.78	2.90	3.10	3.32	3.55	3.75	3.87
	9	40	3.44	0.29	1.458	3.470	0.074	2.90	3.03	3.24	3.47	3.70	3.92	4.04
	10	63	3.63	0.26	1.458	3.609	0.074	3.02	3.15	3.37	3.61	3.85	4.07	4.20
	11	53	3.75	0.27	1.458	3.730	0.074	3.12	3.25	3.48	3.73	3.98	4.21	4.34
	12	42	3.77	0.35	1.458	3.834	0.074	3.20	3.34	3.58	3.83	4.09	4.32	4.46
	13	33	3.95	0.29	1.458	3.927	0.074	3.28	3.42	3.66	3.93	4.19	4.43	4.57
	14	24	4.03	0.22	1.458	4.011	0.074	3.35	3.50	3.74	4.01	4.28	4.52	4.67
	15	23	4.06	0.30	1.458	4.090	0.074	3.42	3.57	3.81	4.09	4.37	4.61	4.76
MC3	6	37	4.50	0.34	1.639	4.520	0.073	3.78	3.94	4.21	4.52	4.82	5.10	5.26
	7	30	4.74	0.33	1.639	4.753	0.073	3.97	4.14	4.43	4.75	5.07	5.36	5.53
	8	43	5.01	0.35	1.639	4.987	0.073	4.17	4.35	4.65	4.99	5.32	5.63	5.81
	9	40	5.14	0.41	1.639	5.211	0.073	4.35	4.54	4.86	5.21	5.56	5.88	6.07
	10	63	5.45	0.38	1.639	5.411	0.073	4.52	4.72	5.05	5.41	5.78	6.10	6.30
	11	53	5.59	0.42	1.639	5.573	0.073	4.66	4.86	5.20	5.57	5.95	6.29	6.49
	12	42	5.58	0.45	1.639	5.702	0.073	4.76	4.97	5.32	5.70	6.09	6.43	6.64
	13	33	5.86	0.48	1.639	5.822	0.073	4.86	5.08	5.43	5.82	6.21	6.57	6.78
	14	24	5.95	0.29	1.639	5.933	0.073	4.96	5.17	5.53	5.93	6.33	6.69	6.91
	15	23	6.02	0.41	1.639	6.037	0.073	5.04	5.26	5.63	6.04	6.44	6.81	7.03
DP4	6	37	1.21	0.10	0.531	1.202	0.093	1.00	1.05	1.12	1.20	1.28	1.36	1.40
	7	30	1.25	0.12	0.531	1.268	0.093	1.06	1.11	1.18	1.27	1.35	1.43	1.48
	8	43	1.35	0.12	0.531	1.336	0.093	1.12	1.16	1.25	1.34	1.43	1.51	1.56
	9	40	1.41	0.15	0.531	1.400	0.093	1.17	1.22	1.31	1.40	1.49	1.58	1.63
	10	63	1.46	0.14	0.531	1.456	0.093	1.22	1.27	1.36	1.46	1.55	1.64	1.70
	11	53	1.51	0.14	0.531	1.503	0.093	1.26	1.31	1.40	1.50	1.60	1.70	1.75
	12	42	1.53	0.18	0.531	1.543	0.093	1.29	1.35	1.44	1.54	1.65	1.74	1.80
	13	33	1.60	0.12	0.531	1.577	0.093	1.32	1.38	1.47	1.58	1.68	1.78	1.84
	14	24	1.60	0.12	0.531	1.606	0.093	1.34	1.40	1.50	1.61	1.71	1.81	1.87
	15	23	1.63	0.14	0.531	1.631	0.093	1.36	1.42	1.52	1.63	1.74	1.84	1.90
MP4	6	37	1.79	0.15	0.465	1.792	0.089	1.50	1.56	1.67	1.79	1.91	2.02	2.09
	7	30	1.87	0.14	0.465	1.878	0.089	1.57	1.64	1.75	1.88	2.00	2.12	2.19
	8	43	1.97	0.18	0.465	1.966	0.089	1.64	1.71	1.83	1.97	2.10	2.22	2.29
	9	40	2.06	0.18	0.465	2.050	0.089	1.71	1.79	1.91	2.05	2.19	2.31	2.39
	10	63	2.14	0.18	0.465	2.129	0.089	1.78	1.86	1.99	2.13	2.27	2.40	2.48
	11	53	2.21	0.20	0.465	2.198	0.089	1.84	1.92	2.05	2.20	2.35	2.48	2.56
	12	42	2.24	0.28	0.465	2.261	0.089	1.89	1.97	2.11	2.26	2.41	2.55	2.63
	13	33	2.33	0.23	0.465	2.320	0.089	1.94	2.02	2.16	2.32	2.48	2.62	2.70
	14	24	2.38	0.14	0.465	2.380	0.089	1.99	2.07	2.22	2.38	2.54	2.68	2.77
	15	23	2.44	0.19	0.465	2.439	0.089	2.04	2.13	2.27	2.44	2.60	2.75	2.84
PP4	6	37	2.81	0.21	1.229	2.824	0.077	2.36	2.46	2.63	2.82	3.01	3.19	3.29
	7	30	2.98	0.20	1.229	2.964	0.077	2.48	2.58	2.76	2.96	3.16	3.34	3.45
	8	43	3.11	0.23	1.229	3.104	0.077	2.59	2.71	2.89	3.10	3.31	3.50	3.61
	9	40	3.21	0.28	1.229	3.241	0.077	2.71	2.83	3.02	3.24	3.46	3.66	3.77
	10	63	3.40	0.24	1.229	3.370	0.077	2.82	2.94	3.14	3.37	3.60	3.80	3.92
	11	53	3.49	0.28	1.229	3.481	0.077	2.91	3.04	3.25	3.48	3.72	3.93	4.05
	12	42	3.53	0.34	1.229	3.577	0.077	2.99	3.12	3.34	3.58	3.82	4.04	4.17
	13	33	3.67	0.31	1.229	3.665	0.077	3.06	3.20	3.42	3.66	3.91	4.13	4.27
	14	24	3.76	0.23	1.229	3.748	0.077	3.13	3.27	3.50	3.75	4.00	4.23	4.36
	15	23	3.81	0.30	1.229	3.830	0.077	3.20	3.34	3.57	3.83	4.09	4.32	4.46
MC4	6	37	3.95	0.31	1.008	3.958	0.078	3.31	3.45	3.69	3.96	4.23	4.47	4.61
	7	30	4.14	0.31	1.008	4.160	0.078	3.48	3.63	3.88	4.16	4.44	4.69	4.84
	8	43	4.40	0.34	1.008	4.363	0.078	3.65	3.80	4.07	4.36	4.66	4.92	5.08
	9	40	4.51	0.35	1.008	4.558	0.078	3.81	3.97	4.25	4.56	4.87	5.14	5.31
	10	63	4.78	0.37	1.008	4.736	0.078	3.96	4.13	4.42	4.74	5.06	5.34	5.52
	11	53	4.91	0.42	1.008	4.885	0.078	4.08	4.26	4.56	4.89	5.21	5.51	5.69
	12	42	4.92	0.43	1.008	5.010	0.078	4.19	4.37	4.67	5.01	5.35	5.65	5.83
	13	33	5.18	0.44	1.008	5.123	0.078	4.28	4.47	4.78	5.12	5.47	5.78	5.97
	14	24	5.23	0.30	1.008	5.228	0.078	4.37	4.56	4.88	5.23	5.58	5.90	6.09
	15	23	5.32	0.36	1.008	5.328	0.078	4.45	4.65	4.97	5.33	5.69	6.01	6.20
DP5	6	37	1.05	0.10	−1.617	1.073	0.108	0.90	0.94	1.00	1.07	1.15	1.21	1.25
	7	30	1.10	0.10	−1.617	1.113	0.108	0.93	0.97	1.04	1.11	1.19	1.26	1.30
	8	43	1.40	1.54	−1.617	1.155	0.108	0.96	1.01	1.08	1.15	1.23	1.30	1.34
	9	40	1.25	0.15	−1.617	1.198	0.108	1.00	1.04	1.12	1.20	1.28	1.35	1.40
	10	63	1.28	0.11	−1.617	1.243	0.108	1.04	1.08	1.16	1.24	1.33	1.40	1.45
	11	53	1.32	0.13	−1.617	1.289	0.108	1.08	1.12	1.20	1.29	1.38	1.45	1.50
	12	42	1.36	0.15	−1.617	1.337	0.108	1.12	1.17	1.25	1.34	1.43	1.51	1.56
	13	33	1.42	0.13	−1.617	1.387	0.108	1.16	1.21	1.29	1.39	1.48	1.56	1.62
	14	24	1.43	0.13	−1.617	1.439	0.108	1.20	1.25	1.34	1.44	1.54	1.62	1.68
	15	23	1.43	0.13	−1.617	1.492	0.108	1.25	1.30	1.39	1.49	1.59	1.68	1.74
MP5	6	37	1.19	0.14	1.713	1.217	0.124	1.02	1.06	1.14	1.22	1.30	1.37	1.42
	7	30	1.24	0.17	1.713	1.262	0.124	1.05	1.10	1.18	1.26	1.35	1.42	1.47
	8	43	1.30	0.16	1.713	1.308	0.124	1.09	1.14	1.22	1.31	1.40	1.48	1.52
	9	40	1.37	0.18	1.713	1.356	0.124	1.13	1.18	1.26	1.36	1.45	1.53	1.58
	10	63	1.40	0.19	1.713	1.406	0.124	1.17	1.23	1.31	1.41	1.50	1.59	1.64
	11	53	1.48	0.18	1.713	1.457	0.124	1.22	1.27	1.36	1.46	1.56	1.64	1.70
	12	42	1.48	0.23	1.713	1.510	0.124	1.26	1.32	1.41	1.51	1.61	1.70	1.76
	13	33	1.56	0.22	1.713	1.565	0.124	1.31	1.36	1.46	1.57	1.67	1.77	1.82
	14	24	1.63	0.13	1.713	1.623	0.124	1.36	1.41	1.51	1.62	1.73	1.83	1.89
	15	23	1.63	0.16	1.713	1.682	0.124	1.41	1.47	1.57	1.68	1.80	1.90	1.96
PP5	6	37	2.15	0.15	0.657	2.159	0.090	1.80	1.88	2.01	2.16	2.30	2.44	2.51
	7	30	2.29	0.16	0.657	2.270	0.090	1.90	1.98	2.12	2.27	2.42	2.56	2.64
	8	43	2.38	0.22	0.657	2.382	0.090	1.99	2.08	2.22	2.38	2.54	2.69	2.77
	9	40	2.47	0.22	0.657	2.493	0.090	2.08	2.17	2.32	2.49	2.66	2.81	2.90
	10	63	2.63	0.30	0.657	2.595	0.090	2.17	2.26	2.42	2.60	2.77	2.93	3.02
	11	53	2.70	0.22	0.657	2.682	0.090	2.24	2.34	2.50	2.68	2.86	3.03	3.12
	12	42	2.71	0.31	0.657	2.754	0.090	2.30	2.40	2.57	2.75	2.94	3.11	3.21
	13	33	2.83	0.24	0.657	2.821	0.090	2.36	2.46	2.63	2.82	3.01	3.18	3.28
	14	24	2.92	0.19	0.657	2.886	0.090	2.41	2.52	2.69	2.89	3.08	3.26	3.36
	15	23	2.93	0.21	0.657	2.948	0.090	2.46	2.57	2.75	2.95	3.15	3.33	3.43
MC5	6	37	3.59	0.26	1.345	3.599	0.076	3.01	3.14	3.36	3.60	3.84	4.06	4.19
	7	30	3.77	0.25	1.345	3.802	0.076	3.18	3.31	3.55	3.80	4.06	4.29	4.43
	8	43	4.03	0.32	1.345	4.006	0.076	3.35	3.49	3.74	4.01	4.28	4.52	4.66
	9	40	4.16	0.33	1.345	4.198	0.076	3.51	3.66	3.91	4.20	4.48	4.74	4.89
	10	63	4.39	0.31	1.345	4.367	0.076	3.65	3.81	4.07	4.37	4.66	4.93	5.09
	11	53	4.52	0.36	1.345	4.506	0.076	3.76	3.93	4.20	4.51	4.81	5.08	5.25
	12	42	4.51	0.45	1.345	4.619	0.076	3.86	4.03	4.31	4.62	4.93	5.21	5.38
	13	33	4.77	0.38	1.345	4.722	0.076	3.95	4.12	4.40	4.72	5.04	5.33	5.50
	14	24	4.85	0.24	1.345	4.812	0.076	4.02	4.20	4.49	4.81	5.14	5.43	5.60
	15	23	4.86	0.33	1.345	4.889	0.076	4.08	4.26	4.56	4.89	5.22	5.52	5.69

Abbreviations: DP1: distal phalanx of the first digit; DP2: distal phalanx of the second finger; DP3: distal phalanx of the third finger; DP4: distal phalanx of the fourth finger; DP5: distal phalanx of the fifth finger; MC1: first metacarpal; MC2: second metacarpal; MC3: third metacarpal; MC4: fourth metacarpal; MC5: fifth metacarpal; MP2: middle phalanx of the second finger; MP3: middle phalanx of the third finger; MP4: middle phalanx of the fourth finger; MP5: middle phalanx of the fifth finger; PP1: proximal phalanx of the first digit; PP2: proximal phalanx of the second finger; PP3: proximal phalanx of the third finger; PP4: proximal phalanx of the fourth finger; PP5: proximal phalanx of the fifth finger.

**TABLE 3:** Age (years), number of individuals (N), mean length (cm), standard deviation (SD), LMS parameters and percentiles of the long bones of the hand by chronological age for males.

	Age	N	Mean	S.D.	L	M	S	P5	P10	P25	P50	P75	P90	P95
DP1	6	42	1.50	0.16	0.027	1.509	0.095	1.26	1.32	1.41	1.51	1.61	1.70	1.76
	7	37	1.59	0.15	0.027	1.568	0.095	1.31	1.37	1.46	1.57	1.67	1.77	1.83
	8	55	1.65	0.17	0.027	1.630	0.095	1.36	1.42	1.52	1.63	1.74	1.84	1.90
	9	49	1.70	0.18	0.027	1.693	0.095	1.41	1.48	1.58	1.69	1.81	1.91	1.97
	10	55	1.76	0.17	0.027	1.760	0.095	1.47	1.53	1.64	1.76	1.88	1.99	2.05
	11	46	1.83	0.16	0.027	1.828	0.095	1.53	1.59	1.70	1.83	1.95	2.06	2.13
	12	62	1.93	0.17	0.027	1.900	0.095	1.59	1.66	1.77	1.90	2.03	2.14	2.21
	13	50	1.99	0.23	0.027	1.974	0.095	1.65	1.72	1.84	1.97	2.11	2.23	2.30
	14	45	2.06	0.16	0.027	2.051	0.095	1.71	1.79	1.91	2.05	2.19	2.31	2.39
	15	43	2.11	0.16	0.027	2.131	0.095	1.78	1.86	1.99	2.13	2.27	2.40	2.48
PP1	6	42	1.97	0.17	−0.392	1.967	0.086	1.64	1.71	1.83	1.97	2.10	2.22	2.29
	7	37	2.08	0.14	−0.392	2.048	0.086	1.71	1.79	1.91	2.05	2.19	2.31	2.39
	8	55	2.14	0.19	−0.392	2.132	0.086	1.78	1.86	1.99	2.13	2.28	2.41	2.48
	9	49	2.25	0.23	−0.392	2.221	0.086	1.86	1.94	2.07	2.22	2.37	2.51	2.59
	10	55	2.31	0.19	−0.392	2.322	0.086	1.94	2.02	2.17	2.32	2.48	2.62	2.70
	11	46	2.41	0.25	−0.392	2.440	0.086	2.04	2.13	2.28	2.44	2.60	2.75	2.84
	12	62	2.61	0.24	−0.392	2.577	0.086	2.15	2.25	2.40	2.58	2.75	2.91	3.00
	13	50	2.78	0.26	−0.392	2.723	0.086	2.28	2.37	2.54	2.72	2.91	3.07	3.17
	14	45	2.88	0.23	−0.392	2.870	0.086	2.40	2.50	2.68	2.87	3.06	3.24	3.34
	15	43	3.00	0.16	−0.392	3.018	0.086	2.52	2.63	2.81	3.02	3.22	3.40	3.51
MC1	6	42	2.93	0.28	−0.759	2.932	0.078	2.45	2.56	2.73	2.93	3.13	3.31	3.41
	7	37	3.13	0.20	−0.759	3.088	0.078	2.58	2.69	2.88	3.09	3.30	3.48	3.60
	8	55	3.28	0.27	−0.759	3.251	0.078	2.72	2.83	3.03	3.25	3.47	3.67	3.79
	9	49	3.44	0.32	−0.759	3.422	0.078	2.86	2.98	3.19	3.42	3.65	3.86	3.98
	10	55	3.62	0.28	−0.759	3.599	0.078	3.01	3.14	3.36	3.60	3.84	4.06	4.19
	11	46	3.75	0.29	−0.759	3.785	0.078	3.16	3.30	3.53	3.78	4.04	4.27	4.41
	12	62	4.03	0.30	−0.759	3.977	0.078	3.32	3.47	3.71	3.98	4.25	4.49	4.63
	13	50	4.25	0.39	−0.759	4.176	0.078	3.49	3.64	3.89	4.18	4.46	4.71	4.86
	14	45	4.41	0.29	−0.759	4.382	0.078	3.66	3.82	4.09	4.38	4.68	4.94	5.10
	15	43	4.53	0.25	−0.759	4.595	0.078	3.84	4.01	4.28	4.59	4.90	5.18	5.35
DP2	6	42	1.09	0.12	−0.630	1.075	0.097	0.90	0.94	1.00	1.07	1.15	1.21	1.25
	7	37	1.13	0.12	−0.630	1.126	0.097	0.94	0.98	1.05	1.13	1.20	1.27	1.31
	8	55	1.20	0.12	−0.630	1.179	0.097	0.99	1.03	1.10	1.18	1.26	1.33	1.37
	9	49	1.24	0.14	−0.630	1.236	0.097	1.03	1.08	1.15	1.24	1.32	1.39	1.44
	10	55	1.31	0.12	−0.630	1.294	0.097	1.08	1.13	1.21	1.29	1.38	1.46	1.51
	11	46	1.36	0.14	−0.630	1.356	0.097	1.13	1.18	1.26	1.36	1.45	1.53	1.58
	12	62	1.44	0.13	−0.630	1.420	0.097	1.19	1.24	1.32	1.42	1.52	1.60	1.65
	13	50	1.53	0.16	−0.630	1.487	0.097	1.24	1.30	1.39	1.49	1.59	1.68	1.73
	14	45	1.56	0.12	−0.630	1.558	0.097	1.30	1.36	1.45	1.56	1.66	1.76	1.81
	15	43	1.61	0.13	−0.630	1.632	0.097	1.36	1.42	1.52	1.63	1.74	1.84	1.90
MP2	6	42	1.48	0.15	−5.057	1.496	0.152	1.25	1.30	1.39	1.50	1.60	1.69	1.74
	7	37	1.58	0.14	−5.057	1.566	0.152	1.31	1.37	1.46	1.57	1.67	1.77	1.82
	8	55	1.61	0.15	−5.057	1.640	0.152	1.37	1.43	1.53	1.64	1.75	1.85	1.91
	9	49	1.73	0.17	−5.057	1.717	0.152	1.43	1.50	1.60	1.72	1.83	1.94	2.00
	10	55	1.74	0.15	−5.057	1.798	0.152	1.50	1.57	1.68	1.80	1.92	2.03	2.09
	11	46	1.83	0.18	−5.057	1.883	0.152	1.57	1.64	1.76	1.88	2.01	2.12	2.19
	12	62	1.95	0.19	−5.057	1.972	0.152	1.65	1.72	1.84	1.97	2.10	2.22	2.30
	13	50	2.04	0.21	−5.057	2.065	0.152	1.73	1.80	1.93	2.06	2.20	2.33	2.40
	14	45	2.12	0.17	−5.057	2.162	0.152	1.81	1.89	2.02	2.16	2.31	2.44	2.52
	15	43	2.18	0.14	−5.057	2.264	0.152	1.89	1.97	2.11	2.26	2.42	2.55	2.64
PP2	6	42	2.63	0.23	0.641	2.638	0.077	2.20	2.30	2.46	2.64	2.82	2.98	3.07
	7	37	2.78	0.18	0.641	2.753	0.077	2.30	2.40	2.57	2.75	2.94	3.11	3.21
	8	55	2.89	0.23	0.641	2.873	0.077	2.40	2.50	2.68	2.87	3.07	3.24	3.35
	9	49	3.02	0.25	0.641	2.998	0.077	2.50	2.61	2.80	3.00	3.20	3.38	3.49
	10	55	3.07	0.22	0.641	3.129	0.077	2.61	2.73	2.92	3.13	3.34	3.53	3.64
	11	46	3.22	0.26	0.641	3.265	0.077	2.73	2.85	3.04	3.26	3.49	3.68	3.80
	12	62	3.43	0.29	0.641	3.407	0.077	2.85	2.97	3.18	3.41	3.64	3.84	3.97
	13	50	3.62	0.31	0.641	3.556	0.077	2.97	3.10	3.32	3.56	3.80	4.01	4.14
	14	45	3.73	0.26	0.641	3.711	0.077	3.10	3.24	3.46	3.71	3.96	4.19	4.32
	15	43	3.84	0.20	0.641	3.872	0.077	3.24	3.38	3.61	3.87	4.13	4.37	4.51
MC2	6	42	4.46	0.39	−0.406	4.482	0.073	3.74	3.91	4.18	4.48	4.78	5.06	5.22
	7	37	4.71	0.30	−0.406	4.692	0.073	3.92	4.09	4.38	4.69	5.01	5.29	5.46
	8	55	4.99	0.40	−0.406	4.911	0.073	4.10	4.28	4.58	4.91	5.24	5.54	5.72
	9	49	5.18	0.44	−0.406	5.141	0.073	4.30	4.48	4.79	5.14	5.49	5.80	5.99
	10	55	5.37	0.36	−0.406	5.382	0.073	4.50	4.69	5.02	5.38	5.74	6.07	6.27
	11	46	5.62	0.41	−0.406	5.634	0.073	4.71	4.91	5.25	5.63	6.01	6.36	6.56
	12	62	5.94	0.40	−0.406	5.897	0.073	4.93	5.14	5.50	5.90	6.29	6.65	6.87
	13	50	6.26	0.51	−0.406	6.173	0.073	5.16	5.38	5.76	6.17	6.59	6.96	7.19
	14	45	6.50	0.41	−0.406	6.461	0.073	5.40	5.63	6.03	6.46	6.90	7.29	7.52
	15	43	6.68	0.35	−0.406	6.763	0.073	5.65	5.90	6.31	6.76	7.22	7.63	7.88
DP3	6	42	1.15	0.12	0.032	1.152	0.100	0.96	1.00	1.07	1.15	1.23	1.30	1.34
	7	37	1.22	0.12	0.032	1.207	0.100	1.01	1.05	1.13	1.21	1.29	1.36	1.41
	8	55	1.28	0.12	0.032	1.265	0.100	1.06	1.10	1.18	1.27	1.35	1.43	1.47
	9	49	1.34	0.17	0.032	1.326	0.100	1.11	1.16	1.24	1.33	1.42	1.50	1.54
	10	55	1.38	0.14	0.032	1.389	0.100	1.16	1.21	1.30	1.39	1.48	1.57	1.62
	11	46	1.45	0.16	0.032	1.455	0.100	1.22	1.27	1.36	1.46	1.55	1.64	1.69
	12	62	1.54	0.14	0.032	1.525	0.100	1.27	1.33	1.42	1.52	1.63	1.72	1.78
	13	50	1.64	0.19	0.032	1.598	0.100	1.33	1.39	1.49	1.60	1.71	1.80	1.86
	14	45	1.69	0.13	0.032	1.674	0.100	1.40	1.46	1.56	1.67	1.79	1.89	1.95
	15	43	1.72	0.13	0.032	1.754	0.100	1.47	1.53	1.64	1.75	1.87	1.98	2.04
MP3	6	42	1.85	0.17	−0.152	1.846	0.084	1.54	1.61	1.72	1.85	1.97	2.08	2.15
	7	37	1.95	0.15	−0.152	1.923	0.084	1.61	1.68	1.79	1.92	2.05	2.17	2.24
	8	55	1.99	0.16	−0.152	2.004	0.084	1.67	1.75	1.87	2.00	2.14	2.26	2.33
	9	49	2.12	0.20	−0.152	2.089	0.084	1.75	1.82	1.95	2.09	2.23	2.36	2.43
	10	55	2.15	0.16	−0.152	2.177	0.084	1.82	1.90	2.03	2.18	2.32	2.46	2.53
	11	46	2.27	0.22	−0.152	2.268	0.084	1.90	1.98	2.12	2.27	2.42	2.56	2.64
	12	62	2.39	0.21	−0.152	2.364	0.084	1.97	2.06	2.20	2.36	2.52	2.67	2.75
	13	50	2.49	0.23	−0.152	2.463	0.084	2.06	2.15	2.30	2.46	2.63	2.78	2.87
	14	45	2.59	0.22	−0.152	2.567	0.084	2.14	2.24	2.39	2.57	2.74	2.90	2.99
	15	43	2.65	0.15	−0.152	2.675	0.084	2.23	2.33	2.49	2.67	2.86	3.02	3.11
PP3	6	42	3.00	0.22	−0.816	2.973	0.072	2.48	2.59	2.77	2.97	3.17	3.35	3.46
	7	37	3.12	0.20	−0.816	3.093	0.072	2.58	2.70	2.88	3.09	3.30	3.49	3.60
	8	55	3.23	0.24	−0.816	3.219	0.072	2.69	2.81	3.00	3.22	3.44	3.63	3.75
	9	49	3.38	0.26	−0.816	3.350	0.072	2.80	2.92	3.12	3.35	3.58	3.78	3.90
	10	55	3.46	0.23	−0.816	3.488	0.072	2.91	3.04	3.25	3.49	3.72	3.94	4.06
	11	46	3.62	0.28	−0.816	3.634	0.072	3.04	3.17	3.39	3.63	3.88	4.10	4.23
	12	62	3.82	0.32	−0.816	3.788	0.072	3.17	3.30	3.53	3.79	4.04	4.27	4.41
	13	50	4.02	0.34	−0.816	3.950	0.072	3.30	3.44	3.68	3.95	4.22	4.46	4.60
	14	45	4.17	0.27	−0.816	4.118	0.072	3.44	3.59	3.84	4.12	4.40	4.65	4.80
	15	43	4.26	0.21	−0.816	4.293	0.072	3.59	3.74	4.00	4.29	4.58	4.84	5.00
MC3	6	42	4.32	0.40	−0.301	4.345	0.076	3.63	3.79	4.05	4.34	4.64	4.90	5.06
	7	37	4.57	0.32	−0.301	4.556	0.076	3.81	3.97	4.25	4.56	4.86	5.14	5.30
	8	55	4.85	0.41	−0.301	4.774	0.076	3.99	4.16	4.45	4.77	5.10	5.39	5.56
	9	49	5.06	0.42	−0.301	4.998	0.076	4.18	4.36	4.66	5.00	5.33	5.64	5.82
	10	55	5.22	0.32	−0.301	5.228	0.076	4.37	4.56	4.88	5.23	5.58	5.90	6.09
	11	46	5.41	0.43	−0.301	5.465	0.076	4.57	4.76	5.10	5.47	5.83	6.17	6.36
	12	62	5.75	0.42	−0.301	5.711	0.076	4.77	4.98	5.33	5.71	6.10	6.44	6.65
	13	50	6.05	0.49	−0.301	5.964	0.076	4.98	5.20	5.56	5.96	6.37	6.73	6.95
	14	45	6.29	0.45	−0.301	6.224	0.076	5.20	5.43	5.80	6.22	6.64	7.02	7.25
	15	43	6.41	0.39	−0.301	6.491	0.076	5.42	5.66	6.05	6.49	6.93	7.32	7.56
DP4	6	42	1.22	0.12	−0.251	1.220	0.099	1.02	1.06	1.14	1.22	1.30	1.38	1.42
	7	37	1.28	0.12	−0.251	1.275	0.099	1.07	1.11	1.19	1.28	1.36	1.44	1.49
	8	55	1.35	0.13	−0.251	1.333	0.099	1.11	1.16	1.24	1.33	1.42	1.50	1.55
	9	49	1.40	0.16	−0.251	1.393	0.099	1.16	1.21	1.30	1.39	1.49	1.57	1.62
	10	55	1.45	0.14	−0.251	1.456	0.099	1.22	1.27	1.36	1.46	1.55	1.64	1.70
	11	46	1.52	0.16	−0.251	1.521	0.099	1.27	1.33	1.42	1.52	1.62	1.72	1.77
	12	62	1.62	0.14	−0.251	1.590	0.099	1.33	1.39	1.48	1.59	1.70	1.79	1.85
	13	50	1.72	0.23	−0.251	1.661	0.099	1.39	1.45	1.55	1.66	1.77	1.87	1.93
	14	45	1.74	0.14	−0.251	1.736	0.099	1.45	1.51	1.62	1.74	1.85	1.96	2.02
	15	43	1.78	0.13	−0.251	1.813	0.099	1.51	1.58	1.69	1.81	1.94	2.05	2.11
MP4	6	42	1.78	0.17	−0.476	1.759	0.083	1.47	1.53	1.64	1.76	1.88	1.98	2.05
	7	37	1.85	0.16	−0.476	1.834	0.083	1.53	1.60	1.71	1.83	1.96	2.07	2.14
	8	55	1.91	0.16	−0.476	1.912	0.083	1.60	1.67	1.78	1.91	2.04	2.16	2.23
	9	49	2.01	0.18	−0.476	1.994	0.083	1.67	1.74	1.86	1.99	2.13	2.25	2.32
	10	55	2.05	0.16	−0.476	2.078	0.083	1.74	1.81	1.94	2.08	2.22	2.34	2.42
	11	46	2.18	0.20	−0.476	2.167	0.083	1.81	1.89	2.02	2.17	2.31	2.44	2.52
	12	62	2.29	0.19	−0.476	2.259	0.083	1.89	1.97	2.11	2.26	2.41	2.55	2.63
	13	50	2.41	0.20	−0.476	2.356	0.083	1.97	2.05	2.20	2.36	2.51	2.66	2.74
	14	45	2.48	0.20	−0.476	2.456	0.083	2.05	2.14	2.29	2.46	2.62	2.77	2.86
	15	43	2.52	0.15	−0.476	2.560	0.083	2.14	2.23	2.39	2.56	2.73	2.89	2.98
PP4	6	42	2.83	0.22	−2.634	2.783	0.075	2.33	2.43	2.60	2.78	2.97	3.14	3.24
	7	37	2.93	0.19	−2.634	2.898	0.075	2.42	2.53	2.70	2.90	3.09	3.27	3.38
	8	55	3.05	0.22	−2.634	3.018	0.075	2.52	2.63	2.81	3.02	3.22	3.41	3.51
	9	49	3.18	0.25	−2.634	3.143	0.075	2.63	2.74	2.93	3.14	3.36	3.55	3.66
	10	55	3.33	0.25	−2.634	3.273	0.075	2.74	2.85	3.05	3.27	3.49	3.69	3.81
	11	46	3.42	0.26	−2.634	3.409	0.075	2.85	2.97	3.18	3.41	3.64	3.85	3.97
	12	62	3.59	0.30	−2.634	3.551	0.075	2.97	3.10	3.31	3.55	3.79	4.01	4.13
	13	50	3.77	0.31	−2.634	3.698	0.075	3.09	3.22	3.45	3.70	3.95	4.17	4.31
	14	45	3.91	0.28	−2.634	3.852	0.075	3.22	3.36	3.59	3.85	4.11	4.35	4.49
	15	43	4.00	0.20	−2.634	4.012	0.075	3.35	3.50	3.74	4.01	4.28	4.53	4.67
MC4	6	42	3.78	0.38	−0.530	3.799	0.079	3.17	3.31	3.54	3.80	4.06	4.29	4.42
	7	37	4.01	0.29	−0.530	3.983	0.079	3.33	3.47	3.71	3.98	4.25	4.49	4.64
	8	55	4.24	0.36	−0.530	4.175	0.079	3.49	3.64	3.89	4.18	4.46	4.71	4.86
	9	49	4.43	0.37	−0.530	4.375	0.079	3.66	3.81	4.08	4.37	4.67	4.94	5.09
	10	55	4.57	0.32	−0.530	4.582	0.079	3.83	3.99	4.27	4.58	4.89	5.17	5.34
	11	46	4.75	0.39	−0.530	4.798	0.079	4.01	4.18	4.47	4.80	5.12	5.41	5.59
	12	62	5.06	0.40	−0.530	5.022	0.079	4.20	4.38	4.68	5.02	5.36	5.67	5.85
	13	50	5.36	0.45	−0.530	5.255	0.079	4.39	4.58	4.90	5.25	5.61	5.93	6.12
	14	45	5.58	0.45	−0.530	5.496	0.079	4.59	4.79	5.12	5.50	5.87	6.20	6.40
	15	43	5.65	0.33	−0.530	5.746	0.079	4.80	5.01	5.36	5.75	6.13	6.48	6.69
DP5	6	42	1.03	0.10	0.437	1.034	0.096	0.86	0.90	0.96	1.03	1.10	1.17	1.20
	7	37	1.10	0.08	0.437	1.095	0.096	0.91	0.95	1.02	1.09	1.17	1.23	1.27
	8	55	1.17	0.12	0.437	1.155	0.096	0.96	1.01	1.08	1.15	1.23	1.30	1.34
	9	49	1.21	0.15	0.437	1.212	0.096	1.01	1.06	1.13	1.21	1.29	1.37	1.41
	10	55	1.27	0.12	0.437	1.272	0.096	1.06	1.11	1.19	1.27	1.36	1.43	1.48
	11	46	1.32	0.15	0.437	1.338	0.096	1.12	1.17	1.25	1.34	1.43	1.51	1.56
	12	62	1.42	0.12	0.437	1.410	0.096	1.18	1.23	1.32	1.41	1.51	1.59	1.64
	13	50	1.50	0.15	0.437	1.478	0.096	1.23	1.29	1.38	1.48	1.58	1.67	1.72
	14	45	1.55	0.10	0.437	1.531	0.096	1.28	1.33	1.43	1.53	1.63	1.73	1.78
	15	43	1.56	0.13	0.437	1.572	0.096	1.31	1.37	1.47	1.57	1.68	1.77	1.83
MP5	6	42	1.18	0.17	1.388	1.181	0.113	0.99	1.03	1.10	1.18	1.26	1.33	1.37
	7	37	1.24	0.15	1.388	1.236	0.113	1.03	1.08	1.15	1.24	1.32	1.39	1.44
	8	55	1.27	0.14	1.388	1.294	0.113	1.08	1.13	1.21	1.29	1.38	1.46	1.51
	9	49	1.37	0.16	1.388	1.355	0.113	1.13	1.18	1.26	1.35	1.45	1.53	1.58
	10	55	1.38	0.16	1.388	1.418	0.113	1.18	1.24	1.32	1.42	1.51	1.60	1.65
	11	46	1.45	0.17	1.388	1.485	0.113	1.24	1.29	1.38	1.48	1.58	1.67	1.73
	12	62	1.57	0.16	1.388	1.554	0.113	1.30	1.36	1.45	1.55	1.66	1.75	1.81
	13	50	1.66	0.18	1.388	1.627	0.113	1.36	1.42	1.52	1.63	1.74	1.84	1.89
	14	45	1.73	0.21	1.388	1.703	0.113	1.42	1.49	1.59	1.70	1.82	1.92	1.98
	15	43	1.76	0.13	1.388	1.783	0.113	1.49	1.55	1.66	1.78	1.90	2.01	2.08
PP5	6	42	2.17	0.17	−0.606	2.143	0.080	1.79	1.87	2.00	2.14	2.29	2.42	2.50
	7	37	2.23	0.16	−0.606	2.228	0.080	1.86	1.94	2.08	2.23	2.38	2.51	2.59
	8	55	2.33	0.20	−0.606	2.318	0.080	1.94	2.02	2.16	2.32	2.47	2.61	2.70
	9	49	2.44	0.22	−0.606	2.413	0.080	2.02	2.10	2.25	2.41	2.58	2.72	2.81
	10	55	2.51	0.18	−0.606	2.517	0.080	2.10	2.19	2.35	2.52	2.69	2.84	2.93
	11	46	2.61	0.23	−0.606	2.630	0.080	2.20	2.29	2.45	2.63	2.81	2.97	3.06
	12	62	2.77	0.24	−0.606	2.753	0.080	2.30	2.40	2.57	2.75	2.94	3.11	3.21
	13	50	2.95	0.27	−0.606	2.881	0.080	2.41	2.51	2.69	2.88	3.08	3.25	3.36
	14	45	3.05	0.24	−0.606	3.013	0.080	2.52	2.63	2.81	3.01	3.22	3.40	3.51
	15	43	3.11	0.17	−0.606	3.149	0.080	2.63	2.75	2.94	3.15	3.36	3.55	3.67
MC5	6	42	3.47	0.31	−0.713	3.485	0.076	2.91	3.04	3.25	3.48	3.72	3.93	4.06
	7	37	3.67	0.24	−0.713	3.658	0.076	3.06	3.19	3.41	3.66	3.90	4.13	4.26
	8	55	3.89	0.31	−0.713	3.838	0.076	3.21	3.35	3.58	3.84	4.10	4.33	4.47
	9	49	4.07	0.34	−0.713	4.027	0.076	3.36	3.51	3.76	4.03	4.30	4.54	4.69
	10	55	4.23	0.31	−0.713	4.224	0.076	3.53	3.68	3.94	4.22	4.51	4.77	4.92
	11	46	4.39	0.35	−0.713	4.429	0.076	3.70	3.86	4.13	4.43	4.73	5.00	5.16
	12	62	4.70	0.35	−0.713	4.644	0.076	3.88	4.05	4.33	4.64	4.96	5.24	5.41
	13	50	4.95	0.43	−0.713	4.867	0.076	4.07	4.24	4.54	4.87	5.20	5.49	5.67
	14	45	5.16	0.37	−0.713	5.100	0.076	4.26	4.45	4.76	5.10	5.44	5.75	5.94
	15	43	5.26	0.30	−0.713	5.342	0.076	4.46	4.66	4.98	5.34	5.70	6.03	6.22

Abbreviations: DP1: distal phalanx of the first digit; DP2: distal phalanx of the second finger; DP3: distal phalanx of the third finger; DP4: distal phalanx of the fourth finger; DP5: distal phalanx of the fifth finger; MC1: first metacarpal; MC2: second metacarpal; MC3: third metacarpal; MC4: fourth metacarpal; MC5: fifth metacarpal. MP2: middle phalanx of the second finger; MP3: middle phalanx of the third finger; MP4: middle phalanx of the fourth finger; MP5: middle phalanx of the fifth finger; PP1: proximal phalanx of the first digit; PP2: proximal phalanx of the second finger; PP3: proximal phalanx of the third finger; PP4: proximal phalanx of the fourth finger; PP5: proximal phalanx of the fifth finger.

There was a strong correlation between all hand length measurements when analyzed by CA and BA. This correlation was observed in the total group (*r* = 0.917; *p* < 0.001), and also when separated by sex (females: *r* = 0.889; *p* < 0.001; and males: *r* = 0.936; *p* < 0.001). Therefore, only the measurements related to CA were described. The data regarding bone age and growth percentile curves are provided in the Supporting Information (Tables S1–S38 and Figures S1–S76).

There was a high level of agreement between the evaluators who measured the length of the phalanges and metacarpals, ranging from *r* = 0.912 (distal phalanx of the first digit) to *r* = 0.938 (metacarpal of the second digit).

Table 4 presents the Z‐scores for hand tubular bones length measurements that showed statistically significant differences compared to population‐based studies conducted in Japan, Nigeria, Sweden, and the United States. For each country, the mean Z‐score was calculated using Brazil as the reference population. The Z‐score reflects the number of standard deviations by which the average measurement of the compared country differs from that of Brazil, with positive values indicating greater measurements and negative values indicating smaller measurements. It is not possible to include *p*‐values in the table because each reported Z value represents the mean of multiple individual Z‐scores, which were calculated separately for each age group within each combination of sex, bone, and digit. Consequently, there is a set of *p*‐values associated with each table row, rather than a single *p*‐value. Since *p*‐values cannot be summarized as a single mean value, no *p*‐value corresponds to the mean Z.

**TABLE 4:** Mean Z‐score values of the other countries in comparison with Brazil, by sex, bone and finger.

Category	Japan	Nigeria	Sweden	USA	Total
*Female (mean)*	*0.590*	*0.872*	*0.386*	*0.652*	*0.625*
Distal phalanx	0.899	0.560	0.466	0.731	0.664
1	0.738	1.112	0.671	0.889	0.853
2	0.957	0.360	0.513	0.523	0.588
3	1.004	0.684	0.406	0.825	0.730
4	0.944	0.596	0.376	0.818	0.683
5	0.855	0.045	0.363	0.599	0.466
Middle phalanx	0.607	0.933	0.541	0.823	0.726
2	0.937	0.660	0.762	1.063	0.856
3	0.593	0.970	0.520	0.879	0.741
4	0.422	0.921	0.314	0.637	0.574
5	0.474	1.181	0.568	0.711	0.733
Proximal phalanx	0.826	1.240	0.449	0.722	0.809
1	0.926	1.235	0.743	0.999	0.976
2	0.947	1.172	0.577	0.644	0.835
3	0.772	1.369	0.340	0.744	0.806
4	0.661	1.255	0.174	0.431	0.630
5	0.821	1.168	0.409	0.793	0.798
Metacarpal	0.033	0.766	0.119	0.366	0.321
1	0.207	0.483	0.106	0.316	0.278
2	0.100	1.045	0.444	0.611	0.550
3	−0.189	0.812	−0.072	0.120	0.168
4	−0.007	0.771	0.033	0.444	0.310
5	0.054	0.719	0.082	0.337	0.298
Male (mean)	0.425	0.711	0.305	0.451	0.473
Distal phalanx	0.748	0.435	0.320	0.633	0.534
1	0.616	0.916	0.527	0.821	0.720
2	0.826	0.301	0.286	0.479	0.473
3	0.883	0.488	0.261	0.642	0.569
4	0.652	0.305	0.139	0.524	0.405
5	0.762	0.167	0.388	0.701	0.504
Middle phalanx	0.471	0.791	0.526	0.624	0.603
2	0.675	0.474	0.841	0.643	0.658
3	0.424	0.819	0.526	0.659	0.607
4	0.333	0.797	0.180	0.533	0.461
5	0.453	1.074	0.559	0.661	0.687
Proximal phalanx	0.630	1.023	0.384	0.546	0.646
1	0.667	0.932	0.570	0.470	0.660
2	0.706	0.857	0.511	0.618	0.673
3	0.663	1.378	0.227	0.567	0.709
4	0.476	0.990	0.206	0.415	0.522
5	0.639	0.959	0.406	0.662	0.666
Metacarpal	−0.138	0.609	0.035	0.033	0.135
1	0.034	0.379	0.071	0.116	0.150
2	−0.071	0.850	0.307	0.185	0.318
3	−0.323	0.656	−0.183	−0.400	−0.062
4	−0.147	0.624	0.061	0.139	0.170
5	−0.185	0.538	−0.083	0.126	0.099
Total	0.508	0.791	0.345	0.551	0.549

Overall, the greatest mean deviations from Brazil were observed in Nigeria (mean Z = 0.79). Smaller mean Z‐scores were found in Sweden (0.35), Japan (0.51), and the United States (0.55). Among females, the mean Z‐scores were higher than those observed in males (0.63 vs. 0.47, respectively). The proximal phalanx showed the highest mean Z‐scores in both sexes (0.81 for females and 0.65 for males), followed by the middle phalanx (0.73 for females and 0.60 for males). The metacarpal showed the smallest average deviations, with overall Z‐score values of 0.32 in females and 0.14 in males.

In the healthy population, phalangeal and metacarpal lengths in females were slightly greater than those in males until approximately 12–13 years of age, after which male measurements surpassed them.

For the patients, 89 radiographs of the left hand and wrist were analyzed. Of this total, 2 were excluded due to incomplete data in their medical records and four due to poor technical quality, leaving a total of 83 patients. The age distribution of the patients is shown in Table 5. The group comprised 18 patients with hypochondroplasia, 27 with achondroplasia, 14 with osteogenesis imperfecta, and 24 with Turner syndrome.

**TABLE 5:** Number of patients by sex and disease and mean and standard deviation of the chronological age of each disease in the group of patients with bone dysplasias.

Disease	N	Age Mean (SD)
Hypochondroplasia	18	8.8 (1.3)
Osteogenesis imperfecta (OI)	14	7.75 (4.2)
Turner syndrome	24	13.5 (3.5)
Achondroplasia	27	7.8 (3.2)

Table 6 presents a comparison of the mean Z‐scores of phalangeal and metacarpal lengths in patients with skeletal dysplasias, stratified by condition. The Table shows that the mean Z score of patients with achondroplasia and hypochondroplasia was lower than that of healthy individuals, with greater reductions being observed in the proximal phalanges and metacarpals. No reduction in the mean Z‐scores beyond −1.4 was observed in patients with OI and there was a maximum reduction of −1.8 SD in the length of the fourth metacarpal in patients with Turner syndrome.

**TABLE 6:** Mean (cm) and standard deviation (SD) of the Z score of patients, calculated from healthy individuals, by disease.

Bone	Achondroplasia (*N* = 27)		Hypochondroplasia (*N* = 18)		Osteogenesis imperfecta (*N* = 14)		Turner syndrome (*N* = 24)	
	Mean	SD	Mean	SD	Mean	SD	Mean	SD
DP1	−2.87	1.5	−0.31	1.3	−0.35	0.4	−0.47	0.7
PP1	−3.40	0.8	0.32	1.5	0.06	0.6	0.06	0.8
MC1	−4.64	0.8	−1.11	0.7	−0.48	1.2	−1.01	1.5
DP2	−2.18	2.0	−2.06	1.0	−0.61	1.0	−0.36	0.8
MP2	−4.14	3.1	−2.10	0.8	−0.47	0.8	−1.13	0.9
PP2	−3.80	0.9	−1.49	1.2	−0.82	1.5	−0.48	1.2
MC2	−4.41	4.3	−2.08	1.2	−0.59	1.1	−0.89	0.8
DP3	−1.63	1.6	−2.22	1.0	−0.14	1.1	−0.94	0.8
MP3	−3.28	2.9	−2.44	0.2	−0.92	2.0	−1.23	0.9
PP3	−4.44	0.9	−1.47	0.7	−0.18	1.5	−0.54	1.1
MC3	−4.17	1.6	−1.91	1.0	−0.38	1.3	−0.67	0.9
DP4	−2.13	2.7	−2.15	1.0	−0.53	1.2	−0.85	0.8
MP4	−3.42	2.3	−2.02	0.7	−1.09	2.0	−0.80	0.9
PP4	−5.74	0.9	−1.14	0.6	−0.44	1.6	−0.67	1.3
MC4	−4.01	1.9	−2.26	1.0	−0.26	1.2	−1.8	0.9
DP5	−2.20	4.8	−2.32	0.8	−0.66	0.7	−0.95	0.7
MP5	−1.41	2.6	−2.061	0.6	−0.87	1.9	−1.13	0.9
PP5	−3.29	0.9	−1.75	1.2	−1.40	1.7	−0.72	1.2
MC5	−3.55	1.0	−1.44	0.4	−1.05	0.7	−0.74	1.1

Abbreviations: DP1: distal phalanx of the first finger; DP2: distal phalanx of the second finger; DP3: distal phalanx of the third finger; DP4: distal phalanx of the fourth finger; DP5: distal phalanx of the fifth finger; MC1: first metacarpal; MC2: second metacarpal; MC3: third metacarpal; MC4: fourth metacarpal; MC5: fifth metacarpal. MP2: middle phalanx of the second finger; MP3: middle phalanx of the third finger; MP4: middle phalanx of the fourth finger; MP5: middle phalanx of the fifth finger; PP1: proximal phalanx of the second finger; PP2: proximal phalanx of the second finger; PP3: proximal phalanx of the third finger; PP4: proximal phalanx of the fourth finger; PP5: proximal phalanx of the fifth finger.

## 5 Discussion

This study assessed the MCPP of healthy individuals in relation to chronological age (CA), bone age (BA), and sex. Although BA estimates do not always align with CA, no significant differences were observed between the two in this cohort, likely due to the inclusion of only healthy subjects. However, in individuals with delayed or advanced skeletal maturation, such discrepancies are more likely to occur, and the use of BA is therefore recommended in these cases.

This is one of the few studies in the literature to apply the LMS method for constructing population‐based percentile intervals, a method that has been increasingly used in recent studies of anthropometric measurements.

The LMS (Lambda‐Mu‐Sigma) method offers several advantages for modeling anthropometric data. By incorporating the Lambda (L) parameter, it accounts for skewness in the data distribution through Box‐Cox transformation, which is particularly important in growth measurements where asymmetry is common. The three parameters (L for skewness, M for the median, and S for dispersion) are modeled as smooth functions of age, allowing the generated curves to accurately reflect changes in distribution over time. The LMS model also enables the calculation of standardized Z‐scores for individual values, even when the data are not normally distributed, facilitating more precise comparisons across individuals and populations (Cole and Green 1992).

Additionally, the smoothing process and the modeling of variability yield percentile curves that are both statistically robust and more representative of real‐world populations, making the LMS method especially valuable in longitudinal and cross‐sectional growth studies involving variables such as bone length. Due to these strengths, the LMS method has been widely adopted by major health organizations, including the World Health Organization (WHO) (de Onis et al. 2007), the Centers for Disease Control and Prevention (CDC) (Kuczmarski et al. 2000), and UK‐WHO (Wright et al. 2010), for the development of official growth reference charts.

Previous population studies conducted in the United States (Garn et al. 1972), Japan (Matsuura and Kajii 1989), Nigeria (Odita et al. 1991), and Sweden (Laurencikas and Rosenborg 2000) employed non‐parametric and empirical methods, such as quantile regression or direct percentile estimation. Although widely used, these approaches present several limitations when compared to the LMS method. Non‐parametric techniques estimate each percentile separately, which may result in non‐smooth or overlapping curves and do not allow for the calculation of standardized Z‐scores, limiting cross‐individual and cross‐population comparisons. Empirical methods are highly sensitive to sample size, lack smoothing, and do not account for age‐related variation in skewness or dispersion. Moreover, they do not support interpolation, extrapolation, or standardization of individual values. In contrast, the LMS method models the full distribution of the data and produces smooth, continuous, and clinically interpretable reference curves, enhancing statistical robustness and comparability.

Considering the methodological variations among the studies, the largest mean deviations from the Brazilian reference were observed in Nigeria (mean Z = 0.79) and Japan (0.51), while the smaller mean Z‐scores were reported in Sweden (0.35) and the United States (0.55). These discrepancies may result from differences in study methodologies and/or changes in population anthropometric characteristics over time. Therefore, this highlights the need for further contemporary population‐based investigations utilizing a standardized LMS‐based comparative framework to enable more precise and reliable cross‐population analyses.

Secular trends in body length provide a sensitive indicator of long‐term shifts in population health, nutrition, and socioeconomic conditions. Over the past 150 years, many populations have shown marked increases in adult stature, particularly in high‐income countries during the early and mid‐20th century, with more variable gains in low‐ and middle‐income settings. Anthropometric evidence demonstrates that increases in height parallel improvements in childhood survival, sanitation, and dietary quality (Robertson et al. 2014).

Comparable trends have been observed in rapidly developing East Asian populations, where improved living conditions and increased protein intake during childhood have supported accelerated height gains (Moon 2011). More recently, however, height increases have plateaued in several populations, including the United States, likely reflecting rising obesity, dietary changes, and persistent socioeconomic disparities (NCD Risk Factor Collaboration (NCD‐RisC) 2016).

On the other hand, bone dysmorphogenesis encompasses abnormalities in skeletal development arising from disruptions in the coordinated processes of cell proliferation, differentiation, patterning, and ossification. Normal bone formation relies on the interplay among chondrocytes, osteoblasts, and osteoclasts, regulated by key signaling pathways such as Indian hedgehog (IHH), parathyroid hormone–related protein (PTHrP), bone morphogenetic proteins (BMP), Wnt/*β*‐catenin signaling pathway, and fibroblast growth factors (FGF). At the growth plate, alterations in endochondral ossification are a major source of dysmorphogenesis. Mutations in cartilage matrix genes, including *COL2A1*, *COMP*, and *ACAN*, disrupt extracellular matrix organization and result in skeletal dysplasias (Long and Ornitz 2013). Any disturbance of these pathways can lead to defects in bone shape and impaired linear growth.

In the healthy population, phalangeal and metacarpal lengths in females were slightly greater than those in males until approximately 12–13 years of age, after which male measurements surpassed them. This is probably due to puberty, which begins earlier in girls. This finding has also been demonstrated in previous population studies (Garn et al. 1972; Laurencikas and Rosenborg 2000; Matsuura and Kajii 1989; Odita et al. 1991).

A reduction in the mean Z‐scores of phalangeal and metacarpal lengths was observed in patients with achondroplasia and hypochondroplasia compared with healthy individuals (Table 4), with more significant reductions in the proximal phalanges and metacarpals, mainly in achondroplasia. These findings are in agreement with the literature, which describes these reductions in length as the most common characteristics affecting the hands of patients with these diseases. (Bober et al. 1993; Heselson et al. 1979; Pauli 2019; Wynn et al. 2007).

As expected, no reduction in the mean Z score was observed in patients with OI compared to healthy individuals, given that this disease is not associated with shortening of the hand bones (Ablin et al. 1990; Renaud et al. 2013), except when there are associated fractures.

In patients with Turner syndrome, a reduction in the mean Z‐score of the fourth metacarpal by 1.8 SD has been demonstrated, a finding similar to that described in the literature, which might be better demonstrated with a larger number of cases (Park 1977; Robinow et al. 1980). It is important to emphasize that the reduction of the fourth metacarpal is not present in all girls with TS; therefore, the absence of this sign does not rule out the possibility of this diagnosis.

Thus, the application of the metacarpophalangeal pattern profile method was able to demonstrate reductions in the length of the phalanges and metacarpals in disorders with markedly affected phenotypes (achondroplasia), less affected phenotypes (hypochondroplasia), and mildly affected phenotypes (Turner syndrome), while no significant reductions were observed in patients with osteogenesis imperfecta, a condition that typically does not present this alteration.

**FIGURE 2:**
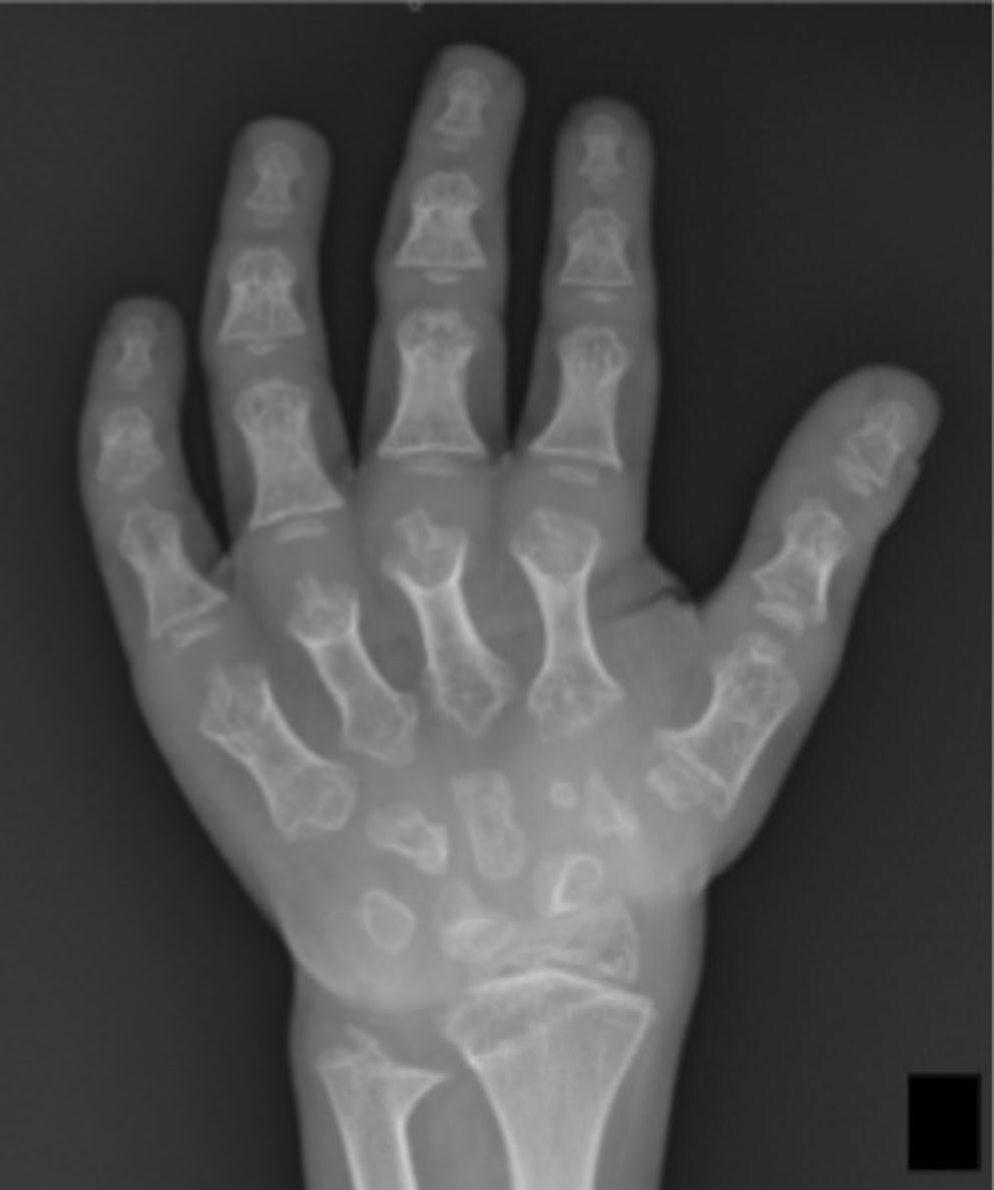
Radiograph of the left hand and wrist of a 7‐year‐old female patient with achondroplasia showing short phalanges and metacarpals, widening of the metaphyseal regions and slight separation between the middle and ring fingers are observed, characterizing the hand with a trident appearance. Negative ulnar variation.

**FIGURE 3:**
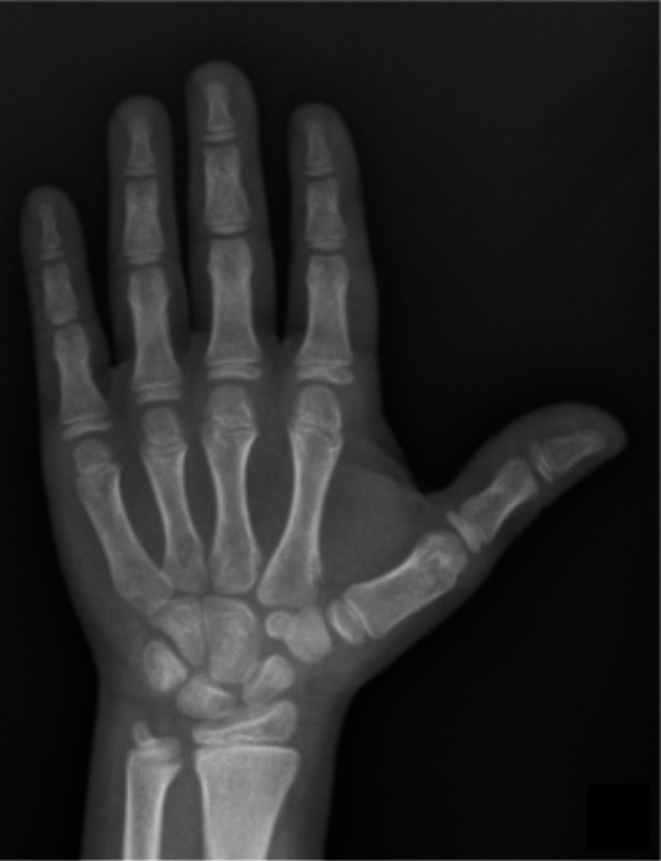
Radiograph of the left hand and wrist of a 10‐year‐old female patient with hypochondroplasia, showing reduced phalangeal and metacarpal lengths of less than 2 SD; however, this alteration is not readily apparent on a cursory analysis.

**FIGURE 4:**
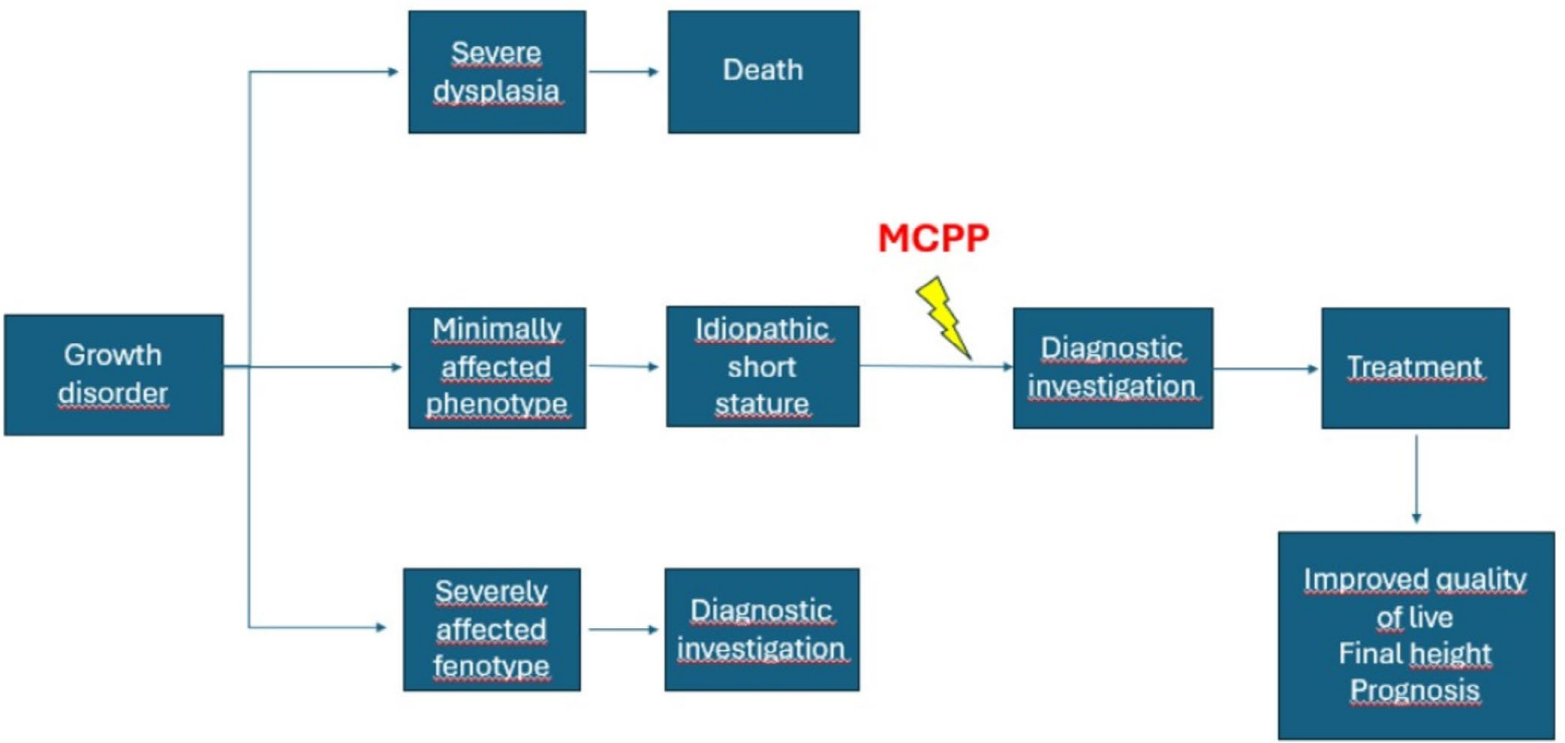
Diagnostic investigation scheme for a patient with growth disorder. (MCPP: Metacarpophalangeal profile).

Severe bone dysplasias usually result in death in the perinatal period (Andersen and Hauge 1989; Laurencikas et al. 2006; Rypens et al. 2006). Less severe dysplasias, but with a highly affected phenotype (like achondroplasia, Figure 2), tend to be suspected in perinatal examinations or in the first years of life (Krakow and Rimoin 2010). Dysplasias with a less affected phenotype may be erroneously classified as idiopathic short stature (ISS) (Grimberg and Allen 2017; Inzaghi et al. 2019), which is a frequent diagnosis in pediatric clinics and is characterized by short stature without systemic, hormonal, or psychosocial causes, and without intrauterine growth restriction. In these patients, like patients with hypochondroplasia (Figure 3), analysis of the MCPP pattern can identify those who present subtle changes in the length of the phalanges and metacarpals that may have gone unnoticed and, thus, better select patients who deserve a more in‐depth diagnostic investigation (Figure 4).

Although many skeletal dysplasias may not have a typical pattern, the identification of a reduction in the length > 2SD of any tubular bone in the hand in MCPP in a patient under investigation for short stature may be a warning sign of a possible dysplastic disease that deserves further investigation. In addition, it is important to assess the morphology of the diaphyses and epiphyses, segmentation defects, bone agenesis, and polydactyly, findings that are valuable for geneticists and may also aid in narrowing the diagnosis of dysplasia syndromes. (Krakow and Rimoin 2010; Ngo et al. 2018; Offiah and Hall 2020; Rypens et al. 2006).

With the discovery of new genes associated with growth cartilage, some patients previously diagnosed with ISS have now received a defined etiological diagnosis. Advances in molecular techniques have linked several genes related to growth cartilage with the etiology of ISS (Grimberg and Allen 2017; Inzaghi et al. 2019) and, as a result, signs such as body disproportion and radiographic changes may help to clarify the diagnosis and potentially predict the response to growth hormone treatment.

It is important to note that left hand and wrist radiography for bone age assessment is routinely requested in the diagnostic evaluation of patients with suspected short stature, and the analysis of the MCPP does not add any additional cost or radiation beyond what is already performed. Furthermore, the assessment of the MCPP can be conducted at any age in patients with growth disorders and may serve as a screening tool to better identify patients who would benefit from further investigation with full‐body radiographs and genetic and biochemical *t*‐tests for diagnostic elucidation, *t*‐tests that are typically more expensive and often less accessible. However, it is important to emphasize that a normal MCPP pattern does not exclude a possible dysplastic disease.

Although MCPP analysis is a method that was developed decades ago, its use in the daily practice of health professionals is still limited. This can be explained by the time required to perform manual measurements, by the fact that many physicians have limited knowledge of what constitutes normal or abnormal measurements, and also because normality standards are based on population studies published decades ago. (Garn et al. 1972; Laurencikas and Rosenborg 2000; Matsuura and Kajii 1989; Odita et al. 1991).

The standardization of phalanges and metacarpals measurements in healthy children and adolescents by age, as presented in this study, represents an important first step toward the development of databases necessary for the implementation of artificial intelligence tools and mobile applications. These technologies could enable the automated assessment of bone age and metacarpophalangeal profiles (MCPP) from a single hand and wrist radiograph, thereby facilitating their use as accessible and efficient initial screening tools in the diagnostic evaluation of patients with growth disorders.

This study has some limitations that should be noted. First, although this study evaluated almost 1000 healthy patients, the number of patients distributed by age group and sex is still relatively small. Second, no correlation was made between the measurements taken and pubertal stage.

Although this study did not evaluate children under 6 years of age or adolescents over 16 years of age, the most severe skeletal dysplasias are usually diagnosed at ages under 6 years and even in utero. Third, although schools from different socioeconomic levels were selected in the city of Sao Paulo, the most populous and mixed‐race city in Brazil, it is not possible to state that the sample used in this study is representative of the country's population. Finally, due to the rarity of the diseases analyzed, the number of patients in the analysis was small. Given these limitations, it is clear that further studies with larger samples of healthy individuals and patients with dysplasias are needed to assess the representativeness and discriminatory capacity of the proposed measures.

## 6 Conclusion

This study described the metacarpophalangeal profile of healthy children and adolescents and established percentile reference intervals using the LMS method. In this population, metacarpophalangeal profile measurements were consistent when assessed by both bone age and chronological age. Comparisons with previous population‐based studies revealed differences in hand long bone lengths, likely reflecting methodological variations and the secular anthropometric evolution since the construction of earlier reference curves. In patients, a reduction in the mean Z score of certain phalanges and metacarpals was observed compared to healthy individuals, supporting the potential utility of metacarpophalangeal profile analysis as an additional tool in the diagnostic evaluation and differentiation of short stature and skeletal dysplasias.

## Author Contributions

**Marcelo Damaso Maruichi:** conceptualization (lead), data curation (lead), formal analysis (lead), investigation (lead), methodology (lead), project administration (lead), resources (lead), validation (lead), visualization (lead), writing – original draft (lead), writing – review and editing (lead). **Bruno Telma Destailleur:** data curation (equal), formal analysis (equal), investigation (equal), resources (equal), validation (equal), visualization (equal). **Giulia Maesta Apelbaum:** data curation (equal), formal analysis (equal), investigation (equal), resources (equal), supervision (equal), validation (equal), visualization (equal). **Carlos Alberto Longui:** conceptualization (lead), formal analysis (lead), methodology (equal), validation (equal). **Cristiane Kochi:** conceptualization (equal), data curation (lead), formal analysis (equal), investigation (equal), methodology (equal), project administration (equal), supervision (equal), validation (equal), visualization (equal), writing – original draft (equal), writing – review and editing (equal).

## Funding

The authors have nothing to report.

## Conflicts of Interest

The authors declare no conflicts of interest.

## Supporting information


**Data S1:** ajhb70212‐sup‐0001‐Supinfo.docx.

## Data Availability

The data that supports the findings of this study are available in the Supporting Information Of This Article.
