# Supplementary material for: The Metacarpophalangeal Pattern Profile: An Old Method With New Insights Into the Evaluation of Short Stature
Source: Am J Hum Biol. 2026 Feb 2;38(2):e70212. doi: 10.1002/ajhb.70212 (PMC12862513; doi:10.1002/ajhb.70212)
Supplement: Supplementary file 1 — Data S1: ajhb70212‐sup‐0001‐Supinfo.docx. [file AJHB-38-e70212-s001.docx]

**Tables**

**Female – Bone Age**

**Table S1.** Age (years), number of individuals (N), mean, standard deviation (S.D.), L, M, and S values, and percentiles of the distal phalanx of the first digit according to bone age in females.

| **Age** | **N** | **Mean** | **S.D.** | **L** | **M** | **S** | **P5** | **P10** | **P25** | **P50** | **P75** | **P90** | **P95** |
| --- | --- | --- | --- | --- | --- | --- | --- | --- | --- | --- | --- | --- | --- |
| 6 | 25 | 1.47 | 0.14 | -0.002 | 1.503 | 0.095 | 1.26 | 1.31 | 1.40 | 1.50 | 1.60 | 1.70 | 1.75 |
| 7 | 32 | 1.59 | 0.17 | -0.002 | 1.547 | 0.095 | 1.29 | 1.35 | 1.44 | 1.55 | 1.65 | 1.75 | 1.80 |
| 8 | 34 | 1.61 | 0.16 | -0.002 | 1.593 | 0.095 | 1.33 | 1.39 | 1.49 | 1.59 | 1.70 | 1.80 | 1.85 |
| 9 | 4 | 1.58 | 0.15 | -0.002 | 1.640 | 0.095 | 1.37 | 1.43 | 1.53 | 1.64 | 1.75 | 1.85 | 1.91 |
| 10 | 43 | 1.69 | 0.15 | -0.002 | 1.688 | 0.095 | 1.41 | 1.47 | 1.57 | 1.69 | 1.80 | 1.90 | 1.97 |
| 11 | 68 | 1.74 | 0.17 | -0.002 | 1.738 | 0.095 | 1.45 | 1.52 | 1.62 | 1.74 | 1.86 | 1.96 | 2.02 |
| 12 | 58 | 1.80 | 0.17 | -0.002 | 1.789 | 0.095 | 1.50 | 1.56 | 1.67 | 1.79 | 1.91 | 2.02 | 2.08 |
| 13 | 44 | 1.85 | 0.21 | -0.002 | 1.842 | 0.095 | 1.54 | 1.61 | 1.72 | 1.84 | 1.97 | 2.08 | 2.15 |
| 14 | 17 | 1.86 | 0.13 | -0.002 | 1.897 | 0.095 | 1.58 | 1.65 | 1.77 | 1.90 | 2.02 | 2.14 | 2.21 |
| 15 | 26 | 1.98 | 0.14 | -0.002 | 1.953 | 0.095 | 1.63 | 1.70 | 1.82 | 1.95 | 2.08 | 2.20 | 2.27 |

**Table S2.** Age (years), number of individuals (N), mean, standard deviation (S.D.), L, M, and S values, and percentiles of the proximal phalanx of the first digit according to bone age in females.

| **Age** | **N** | **Mean** | **S.D.** | **L** | **M** | **S** | **P5** | **P10** | **P25** | **P50** | **P75** | **P90** | **P95** |
| --- | --- | --- | --- | --- | --- | --- | --- | --- | --- | --- | --- | --- | --- |
| 6 | 25 | 1.96 | 0.12 | 1.364 | 1.970 | 0.083 | 1.65 | 1.72 | 1.84 | 1.97 | 2.10 | 2.22 | 2.29 |
| 7 | 32 | 2.05 | 0.15 | 1.364 | 2.047 | 0.083 | 1.71 | 1.78 | 1.91 | 2.05 | 2.18 | 2.31 | 2.38 |
| 8 | 34 | 2.14 | 0.19 | 1.364 | 2.127 | 0.083 | 1.78 | 1.85 | 1.98 | 2.13 | 2.27 | 2.40 | 2.48 |
| 9 | 4 | 2.15 | 0.13 | 1.364 | 2.210 | 0.083 | 1.85 | 1.93 | 2.06 | 2.21 | 2.36 | 2.49 | 2.57 |
| 10 | 43 | 2.26 | 0.19 | 1.364 | 2.296 | 0.083 | 1.92 | 2.00 | 2.14 | 2.30 | 2.45 | 2.59 | 2.67 |
| 11 | 68 | 2.36 | 0.21 | 1.364 | 2.385 | 0.083 | 1.99 | 2.08 | 2.22 | 2.39 | 2.55 | 2.69 | 2.78 |
| 12 | 58 | 2.52 | 0.20 | 1.364 | 2.478 | 0.083 | 2.07 | 2.16 | 2.31 | 2.48 | 2.65 | 2.80 | 2.89 |
| 13 | 44 | 2.60 | 0.26 | 1.364 | 2.574 | 0.083 | 2.15 | 2.24 | 2.40 | 2.57 | 2.75 | 2.90 | 3.00 |
| 14 | 17 | 2.65 | 0.17 | 1.364 | 2.675 | 0.083 | 2.23 | 2.33 | 2.49 | 2.67 | 2.86 | 3.02 | 3.11 |
| 15 | 26 | 2.72 | 0.23 | 1.364 | 2.779 | 0.083 | 2.32 | 2.42 | 2.59 | 2.78 | 2.97 | 3.13 | 3.24 |

**Table S3.** Age (years), number of individuals (N), mean, standard deviation (S.D.), L, M, and S values, and percentiles of the first metacarpal according to bone age in females.

| **Age** | **N** | **Mean** | **S.D.** | **L** | **M** | **S** | **P5** | **P10** | **P25** | **P50** | **P75** | **P90** | **P95** |
| --- | --- | --- | --- | --- | --- | --- | --- | --- | --- | --- | --- | --- | --- |
| 6 | 25 | 3.06 | 0.16 | 1.101 | 3.054 | 0.069 | 2.55 | 2.66 | 2.85 | 3.05 | 3.26 | 3.45 | 3.56 |
| 7 | 32 | 3.18 | 0.21 | 1.101 | 3.169 | 0.069 | 2.65 | 2.76 | 2.96 | 3.17 | 3.38 | 3.57 | 3.69 |
| 8 | 34 | 3.29 | 0.24 | 1.101 | 3.287 | 0.069 | 2.75 | 2.87 | 3.06 | 3.29 | 3.51 | 3.71 | 3.83 |
| 9 | 4 | 3.40 | 0.26 | 1.101 | 3.412 | 0.069 | 2.85 | 2.97 | 3.18 | 3.41 | 3.64 | 3.85 | 3.97 |
| 10 | 43 | 3.52 | 0.22 | 1.101 | 3.555 | 0.069 | 2.97 | 3.10 | 3.32 | 3.56 | 3.80 | 4.01 | 4.14 |
| 11 | 68 | 3.72 | 0.29 | 1.101 | 3.719 | 0.069 | 3.11 | 3.24 | 3.47 | 3.72 | 3.97 | 4.20 | 4.33 |
| 12 | 58 | 3.90 | 0.22 | 1.101 | 3.881 | 0.069 | 3.24 | 3.38 | 3.62 | 3.88 | 4.14 | 4.38 | 4.52 |
| 13 | 44 | 4.02 | 0.37 | 1.101 | 4.012 | 0.069 | 3.35 | 3.50 | 3.74 | 4.01 | 4.28 | 4.53 | 4.67 |
| 14 | 17 | 4.11 | 0.26 | 1.101 | 4.102 | 0.069 | 3.43 | 3.58 | 3.83 | 4.10 | 4.38 | 4.63 | 4.78 |
| 15 | 26 | 4.14 | 0.24 | 1.101 | 4.167 | 0.069 | 3.48 | 3.63 | 3.89 | 4.17 | 4.45 | 4.70 | 4.85 |

**Table S4.** Age (years), number of individuals (N), mean, standard deviation (S.D.), L, M, and S values, and percentiles of the distal phalanx of the second digit according to bone age in females.

| **Age** | **N** | **Mean** | **S.D.** | **L** | **M** | **S** | **P5** | **P10** | **P25** | **P50** | **P75** | **P90** | **P95** |
| --- | --- | --- | --- | --- | --- | --- | --- | --- | --- | --- | --- | --- | --- |
| 6 | 25 | 1.10 | 0.09 | 0.926 | 1.101 | 0.089 | 0.92 | 0.96 | 1.03 | 1.10 | 1.18 | 1.24 | 1.28 |
| 7 | 32 | 1.14 | 0.10 | 0.926 | 1.142 | 0.089 | 0.95 | 1.00 | 1.07 | 1.14 | 1.22 | 1.29 | 1.33 |
| 8 | 34 | 1.19 | 0.10 | 0.926 | 1.185 | 0.089 | 0.99 | 1.03 | 1.11 | 1.19 | 1.27 | 1.34 | 1.38 |
| 9 | 4 | 1.28 | 0.10 | 0.926 | 1.229 | 0.089 | 1.03 | 1.07 | 1.15 | 1.23 | 1.31 | 1.39 | 1.43 |
| 10 | 43 | 1.27 | 0.11 | 0.926 | 1.273 | 0.089 | 1.06 | 1.11 | 1.19 | 1.27 | 1.36 | 1.44 | 1.48 |
| 11 | 68 | 1.31 | 0.12 | 0.926 | 1.318 | 0.089 | 1.10 | 1.15 | 1.23 | 1.32 | 1.41 | 1.49 | 1.53 |
| 12 | 58 | 1.37 | 0.12 | 0.926 | 1.362 | 0.089 | 1.14 | 1.19 | 1.27 | 1.36 | 1.45 | 1.54 | 1.59 |
| 13 | 44 | 1.43 | 0.14 | 0.926 | 1.406 | 0.089 | 1.17 | 1.23 | 1.31 | 1.41 | 1.50 | 1.59 | 1.64 |
| 14 | 17 | 1.42 | 0.08 | 0.926 | 1.449 | 0.089 | 1.21 | 1.26 | 1.35 | 1.45 | 1.55 | 1.63 | 1.69 |
| 15 | 26 | 1.47 | 0.14 | 0.926 | 1.492 | 0.089 | 1.25 | 1.30 | 1.39 | 1.49 | 1.59 | 1.68 | 1.74 |

**Table S5.** Age (years), number of individuals (N), mean, standard deviation (S.D.), L, M, and S values, and percentiles of the middle phalanx of the second digit according to bone age in females.

| **Age** | **N** | **Mean** | **S.D.** | **L** | **M** | **S** | **P5** | **P10** | **P25** | **P50** | **P75** | **P90** | **P95** |
| --- | --- | --- | --- | --- | --- | --- | --- | --- | --- | --- | --- | --- | --- |
| 6 | 25 | 1.51 | 0.11 | 1.578 | 1.524 | 0.085 | 1.27 | 1.33 | 1.42 | 1.52 | 1.63 | 1.72 | 1.78 |
| 7 | 32 | 1.59 | 0.13 | 1.578 | 1.579 | 0.085 | 1.32 | 1.38 | 1.47 | 1.58 | 1.69 | 1.78 | 1.84 |
| 8 | 34 | 1.64 | 0.17 | 1.578 | 1.635 | 0.085 | 1.37 | 1.43 | 1.52 | 1.63 | 1.75 | 1.84 | 1.90 |
| 9 | 4 | 1.68 | 0.15 | 1.578 | 1.693 | 0.085 | 1.41 | 1.48 | 1.58 | 1.69 | 1.81 | 1.91 | 1.97 |
| 10 | 43 | 1.73 | 0.14 | 1.578 | 1.754 | 0.085 | 1.47 | 1.53 | 1.64 | 1.75 | 1.87 | 1.98 | 2.04 |
| 11 | 68 | 1.80 | 0.15 | 1.578 | 1.816 | 0.085 | 1.52 | 1.58 | 1.69 | 1.82 | 1.94 | 2.05 | 2.11 |
| 12 | 58 | 1.89 | 0.15 | 1.578 | 1.881 | 0.085 | 1.57 | 1.64 | 1.75 | 1.88 | 2.01 | 2.12 | 2.19 |
| 13 | 44 | 1.96 | 0.21 | 1.578 | 1.948 | 0.085 | 1.63 | 1.70 | 1.82 | 1.95 | 2.08 | 2.20 | 2.27 |
| 14 | 17 | 2.04 | 0.14 | 1.578 | 2.017 | 0.085 | 1.69 | 1.76 | 1.88 | 2.02 | 2.15 | 2.28 | 2.35 |
| 15 | 26 | 2.05 | 0.17 | 1.578 | 2.089 | 0.085 | 1.75 | 1.82 | 1.95 | 2.09 | 2.23 | 2.36 | 2.43 |

**Table S6.** Age (years), number of individuals (N), mean, standard deviation (S.D.), L, M, and S values, and percentiles of the proximal phalanx of the second digit according to bone age in females.

| **Age** | **N** | **Mean** | **S.D.** | **L** | **M** | **S** | **P5** | **P10** | **P25** | **P50** | **P75** | **P90** | **P95** |
| --- | --- | --- | --- | --- | --- | --- | --- | --- | --- | --- | --- | --- | --- |
| 6 | 25 | 2.65 | 0.16 | 1.187 | 2.688 | 0.070 | 2.25 | 2.34 | 2.51 | 2.69 | 2.87 | 3.03 | 3.13 |
| 7 | 32 | 2.80 | 0.18 | 1.187 | 2.783 | 0.070 | 2.33 | 2.43 | 2.60 | 2.78 | 2.97 | 3.14 | 3.24 |
| 8 | 34 | 2.92 | 0.25 | 1.187 | 2.881 | 0.070 | 2.41 | 2.51 | 2.69 | 2.88 | 3.08 | 3.25 | 3.36 |
| 9 | 4 | 2.90 | 0.24 | 1.187 | 2.983 | 0.070 | 2.49 | 2.60 | 2.78 | 2.98 | 3.18 | 3.37 | 3.47 |
| 10 | 43 | 3.06 | 0.19 | 1.187 | 3.088 | 0.070 | 2.58 | 2.69 | 2.88 | 3.09 | 3.30 | 3.48 | 3.60 |
| 11 | 68 | 3.17 | 0.21 | 1.187 | 3.197 | 0.070 | 2.67 | 2.79 | 2.98 | 3.20 | 3.41 | 3.61 | 3.72 |
| 12 | 58 | 3.34 | 0.22 | 1.187 | 3.310 | 0.070 | 2.77 | 2.89 | 3.09 | 3.31 | 3.53 | 3.73 | 3.85 |
| 13 | 44 | 3.46 | 0.31 | 1.187 | 3.426 | 0.070 | 2.86 | 2.99 | 3.20 | 3.43 | 3.66 | 3.87 | 3.99 |
| 14 | 17 | 3.53 | 0.21 | 1.187 | 3.547 | 0.070 | 2.96 | 3.09 | 3.31 | 3.55 | 3.79 | 4.00 | 4.13 |
| 15 | 26 | 3.61 | 0.23 | 1.187 | 3.672 | 0.070 | 3.07 | 3.20 | 3.42 | 3.67 | 3.92 | 4.14 | 4.28 |

**Table S7.** Age (years), number of individuals (N), mean, standard deviation (S.D.), L, M, and S values, and percentiles of the second metacarpal according to bone age in females.

| **Age** | **N** | **Mean** | **S.D.** | **L** | **M** | **S** | **P5** | **P10** | **P25** | **P50** | **P75** | **P90** | **P95** |
| --- | --- | --- | --- | --- | --- | --- | --- | --- | --- | --- | --- | --- | --- |
| 6 | 25 | 4.57 | 0.30 | 1.986 | 4.622 | 0.065 | 3.86 | 4.03 | 4.31 | 4.62 | 4.93 | 5.21 | 5.38 |
| 7 | 32 | 4.84 | 0.33 | 1.986 | 4.806 | 0.065 | 4.02 | 4.19 | 4.48 | 4.81 | 5.13 | 5.42 | 5.60 |
| 8 | 34 | 4.97 | 0.37 | 1.986 | 4.978 | 0.065 | 4.16 | 4.34 | 4.64 | 4.98 | 5.31 | 5.62 | 5.80 |
| 9 | 4 | 5.10 | 0.34 | 1.986 | 5.144 | 0.065 | 4.30 | 4.48 | 4.80 | 5.14 | 5.49 | 5.80 | 5.99 |
| 10 | 43 | 5.26 | 0.34 | 1.986 | 5.324 | 0.065 | 4.45 | 4.64 | 4.96 | 5.32 | 5.68 | 6.01 | 6.20 |
| 11 | 68 | 5.51 | 0.33 | 1.986 | 5.530 | 0.065 | 4.62 | 4.82 | 5.16 | 5.53 | 5.90 | 6.24 | 6.44 |
| 12 | 58 | 5.78 | 0.36 | 1.986 | 5.744 | 0.065 | 4.80 | 5.01 | 5.36 | 5.74 | 6.13 | 6.48 | 6.69 |
| 13 | 44 | 5.89 | 0.50 | 1.986 | 5.928 | 0.065 | 4.95 | 5.17 | 5.53 | 5.93 | 6.33 | 6.69 | 6.90 |
| 14 | 17 | 6.13 | 0.37 | 1.986 | 6.070 | 0.065 | 5.07 | 5.29 | 5.66 | 6.07 | 6.48 | 6.85 | 7.07 |
| 15 | 26 | 6.14 | 0.29 | 1.986 | 6.182 | 0.065 | 5.17 | 5.39 | 5.77 | 6.18 | 6.60 | 6.97 | 7.20 |

**Table S8.** Age (years), number of individuals (N), mean, standard deviation (S.D.), L, M, and S values, and percentiles of the distal phalanx of the third digit according to bone age in females.

| **Age** | **N** | **Mean** | **S.D.** | **L** | **M** | **S** | **P5** | **P10** | **P25** | **P50** | **P75** | **P90** | **P95** |
| --- | --- | --- | --- | --- | --- | --- | --- | --- | --- | --- | --- | --- | --- |
| 6 | 25 | 1.15 | 0.10 | 1.087 | 1.174 | 0.093 | 0.98 | 1.02 | 1.09 | 1.17 | 1.25 | 1.32 | 1.37 |
| 7 | 32 | 1.23 | 0.12 | 1.087 | 1.215 | 0.093 | 1.02 | 1.06 | 1.13 | 1.21 | 1.30 | 1.37 | 1.41 |
| 8 | 34 | 1.27 | 0.12 | 1.087 | 1.257 | 0.093 | 1.05 | 1.10 | 1.17 | 1.26 | 1.34 | 1.42 | 1.46 |
| 9 | 4 | 1.30 | 0.12 | 1.087 | 1.301 | 0.093 | 1.09 | 1.13 | 1.21 | 1.30 | 1.39 | 1.47 | 1.52 |
| 10 | 43 | 1.33 | 0.12 | 1.087 | 1.347 | 0.093 | 1.13 | 1.17 | 1.26 | 1.35 | 1.44 | 1.52 | 1.57 |
| 11 | 68 | 1.38 | 0.13 | 1.087 | 1.394 | 0.093 | 1.16 | 1.22 | 1.30 | 1.39 | 1.49 | 1.57 | 1.62 |
| 12 | 58 | 1.47 | 0.14 | 1.087 | 1.442 | 0.093 | 1.21 | 1.26 | 1.34 | 1.44 | 1.54 | 1.63 | 1.68 |
| 13 | 44 | 1.50 | 0.17 | 1.087 | 1.493 | 0.093 | 1.25 | 1.30 | 1.39 | 1.49 | 1.59 | 1.68 | 1.74 |
| 14 | 17 | 1.54 | 0.11 | 1.087 | 1.545 | 0.093 | 1.29 | 1.35 | 1.44 | 1.54 | 1.65 | 1.74 | 1.80 |
| 15 | 26 | 1.57 | 0.12 | 1.087 | 1.598 | 0.093 | 1.34 | 1.39 | 1.49 | 1.60 | 1.71 | 1.80 | 1.86 |

**Table S9.** Age (years), number of individuals (N), mean, standard deviation (S.D.), L, M, and S values, and percentiles of the middle phalanx of the third digit according to bone age in females.

| **Age** | **N** | **Mean** | **S.D.** | **L** | **M** | **S** | **P5** | **P10** | **P25** | **P50** | **P75** | **P90** | **P95** |
| --- | --- | --- | --- | --- | --- | --- | --- | --- | --- | --- | --- | --- | --- |
| 6 | 25 | 1.87 | 0.11 | 1.630 | 1.888 | 0.077 | 1.58 | 1.65 | 1.76 | 1.89 | 2.02 | 2.13 | 2.20 |
| 7 | 32 | 1.97 | 0.15 | 1.630 | 1.952 | 0.077 | 1.63 | 1.70 | 1.82 | 1.95 | 2.08 | 2.20 | 2.27 |
| 8 | 34 | 2.04 | 0.17 | 1.630 | 2.017 | 0.077 | 1.69 | 1.76 | 1.88 | 2.02 | 2.15 | 2.28 | 2.35 |
| 9 | 4 | 2.10 | 0.18 | 1.630 | 2.085 | 0.077 | 1.74 | 1.82 | 1.94 | 2.09 | 2.23 | 2.35 | 2.43 |
| 10 | 43 | 2.13 | 0.14 | 1.630 | 2.155 | 0.077 | 1.80 | 1.88 | 2.01 | 2.16 | 2.30 | 2.43 | 2.51 |
| 11 | 68 | 2.19 | 0.17 | 1.630 | 2.228 | 0.077 | 1.86 | 1.94 | 2.08 | 2.23 | 2.38 | 2.51 | 2.59 |
| 12 | 58 | 2.33 | 0.18 | 1.630 | 2.303 | 0.077 | 1.92 | 2.01 | 2.15 | 2.30 | 2.46 | 2.60 | 2.68 |
| 13 | 44 | 2.39 | 0.23 | 1.630 | 2.380 | 0.077 | 1.99 | 2.08 | 2.22 | 2.38 | 2.54 | 2.69 | 2.77 |
| 14 | 17 | 2.48 | 0.18 | 1.630 | 2.460 | 0.077 | 2.06 | 2.14 | 2.29 | 2.46 | 2.63 | 2.78 | 2.86 |
| 15 | 26 | 2.50 | 0.20 | 1.630 | 2.543 | 0.077 | 2.12 | 2.22 | 2.37 | 2.54 | 2.71 | 2.87 | 2.96 |

**Table S10.** Age (years), number of individuals (N), mean, standard deviation (S.D.), L, M, and S values, and percentiles of the proximal phalanx of the third digit according to bone age in females.

| **Age** | **N** | **Mean** | **S.D.** | **L** | **M** | **S** | **P5** | **P10** | **P25** | **P50** | **P75** | **P90** | **P95** |
| --- | --- | --- | --- | --- | --- | --- | --- | --- | --- | --- | --- | --- | --- |
| 6 | 25 | 2.98 | 0.19 | 1.636 | 3.023 | 0.068 | 2.53 | 2.64 | 2.82 | 3.02 | 3.23 | 3.41 | 3.52 |
| 7 | 32 | 3.16 | 0.22 | 1.636 | 3.127 | 0.068 | 2.61 | 2.73 | 2.92 | 3.13 | 3.34 | 3.53 | 3.64 |
| 8 | 34 | 3.26 | 0.24 | 1.636 | 3.235 | 0.068 | 2.70 | 2.82 | 3.02 | 3.24 | 3.45 | 3.65 | 3.77 |
| 9 | 4 | 3.20 | 0.18 | 1.636 | 3.347 | 0.068 | 2.80 | 2.92 | 3.12 | 3.35 | 3.57 | 3.78 | 3.90 |
| 10 | 43 | 3.43 | 0.22 | 1.636 | 3.463 | 0.068 | 2.89 | 3.02 | 3.23 | 3.46 | 3.70 | 3.91 | 4.03 |
| 11 | 68 | 3.54 | 0.23 | 1.636 | 3.582 | 0.068 | 2.99 | 3.12 | 3.34 | 3.58 | 3.82 | 4.04 | 4.17 |
| 12 | 58 | 3.74 | 0.24 | 1.636 | 3.706 | 0.068 | 3.10 | 3.23 | 3.46 | 3.71 | 3.96 | 4.18 | 4.32 |
| 13 | 44 | 3.86 | 0.33 | 1.636 | 3.834 | 0.068 | 3.20 | 3.34 | 3.58 | 3.83 | 4.09 | 4.33 | 4.46 |
| 14 | 17 | 4.00 | 0.22 | 1.636 | 3.967 | 0.068 | 3.31 | 3.46 | 3.70 | 3.97 | 4.23 | 4.47 | 4.62 |
| 15 | 26 | 4.02 | 0.28 | 1.636 | 4.103 | 0.068 | 3.43 | 3.58 | 3.83 | 4.10 | 4.38 | 4.63 | 4.78 |

**Table S11.** Age (years), number of individuals (N), mean, standard deviation (S.D.), L, M, and S values, and percentiles of the third metacarpal according to bone age in females.

| **Age** | **N** | **Mean** | **S.D.** | **L** | **M** | **S** | **P5** | **P10** | **P25** | **P50** | **P75** | **P90** | **P95** |
| --- | --- | --- | --- | --- | --- | --- | --- | --- | --- | --- | --- | --- | --- |
| 6 | 25 | 4.44 | 0.29 | 1.739 | 4.502 | 0.066 | 3.76 | 3.93 | 4.20 | 4.50 | 4.81 | 5.08 | 5.24 |
| 7 | 32 | 4.72 | 0.32 | 1.739 | 4.678 | 0.066 | 3.91 | 4.08 | 4.36 | 4.68 | 4.99 | 5.28 | 5.45 |
| 8 | 34 | 4.86 | 0.35 | 1.739 | 4.850 | 0.066 | 4.05 | 4.23 | 4.52 | 4.85 | 5.18 | 5.47 | 5.65 |
| 9 | 4 | 4.98 | 0.39 | 1.739 | 5.019 | 0.066 | 4.19 | 4.38 | 4.68 | 5.02 | 5.36 | 5.66 | 5.84 |
| 10 | 43 | 5.13 | 0.31 | 1.739 | 5.192 | 0.066 | 4.34 | 4.53 | 4.84 | 5.19 | 5.54 | 5.86 | 6.05 |
| 11 | 68 | 5.35 | 0.35 | 1.739 | 5.376 | 0.066 | 4.49 | 4.69 | 5.01 | 5.38 | 5.74 | 6.06 | 6.26 |
| 12 | 58 | 5.60 | 0.38 | 1.739 | 5.560 | 0.066 | 4.65 | 4.85 | 5.19 | 5.56 | 5.94 | 6.27 | 6.47 |
| 13 | 44 | 5.72 | 0.47 | 1.739 | 5.729 | 0.066 | 4.79 | 4.99 | 5.34 | 5.73 | 6.12 | 6.46 | 6.67 |
| 14 | 17 | 5.93 | 0.35 | 1.739 | 5.877 | 0.066 | 4.91 | 5.12 | 5.48 | 5.88 | 6.27 | 6.63 | 6.84 |
| 15 | 26 | 5.95 | 0.29 | 1.739 | 6.012 | 0.066 | 5.02 | 5.24 | 5.61 | 6.01 | 6.42 | 6.78 | 7.00 |

**Table S12.** Age (years), number of individuals (N), mean, standard deviation (S.D.), L, M, and S values, and percentiles of the distal phalanx of the fourth digit according to bone age in females.

| **Age** | **N** | **Mean** | **S.D.** | **L** | **M** | **S** | **P5** | **P10** | **P25** | **P50** | **P75** | **P90** | **P95** |
| --- | --- | --- | --- | --- | --- | --- | --- | --- | --- | --- | --- | --- | --- |
| 6 | 25 | 1.20 | 0.08 | 1.160 | 1.210 | 0.088 | 1.01 | 1.05 | 1.13 | 1.21 | 1.29 | 1.36 | 1.41 |
| 7 | 32 | 1.25 | 0.11 | 1.160 | 1.253 | 0.088 | 1.05 | 1.09 | 1.17 | 1.25 | 1.34 | 1.41 | 1.46 |
| 8 | 34 | 1.32 | 0.11 | 1.160 | 1.298 | 0.088 | 1.08 | 1.13 | 1.21 | 1.30 | 1.39 | 1.46 | 1.51 |
| 9 | 4 | 1.33 | 0.05 | 1.160 | 1.345 | 0.088 | 1.12 | 1.17 | 1.25 | 1.35 | 1.44 | 1.52 | 1.57 |
| 10 | 43 | 1.39 | 0.12 | 1.160 | 1.394 | 0.088 | 1.16 | 1.22 | 1.30 | 1.39 | 1.49 | 1.57 | 1.62 |
| 11 | 68 | 1.42 | 0.13 | 1.160 | 1.444 | 0.088 | 1.21 | 1.26 | 1.35 | 1.44 | 1.54 | 1.63 | 1.68 |
| 12 | 58 | 1.52 | 0.13 | 1.160 | 1.496 | 0.088 | 1.25 | 1.30 | 1.40 | 1.50 | 1.60 | 1.69 | 1.74 |
| 13 | 44 | 1.57 | 0.17 | 1.160 | 1.550 | 0.088 | 1.30 | 1.35 | 1.45 | 1.55 | 1.65 | 1.75 | 1.81 |
| 14 | 17 | 1.60 | 0.12 | 1.160 | 1.606 | 0.088 | 1.34 | 1.40 | 1.50 | 1.61 | 1.71 | 1.81 | 1.87 |
| 15 | 26 | 1.63 | 0.12 | 1.160 | 1.664 | 0.088 | 1.39 | 1.45 | 1.55 | 1.66 | 1.78 | 1.88 | 1.94 |

**Table S13.** Age (years), number of individuals (N), mean, standard deviation (S.D.), L, M, and S values, and percentiles of the middle phalanx of the fourth digit according to bone age in females.

| **Age** | **N** | **Mean** | **S.D.** | **L** | **M** | **S** | **P5** | **P10** | **P25** | **P50** | **P75** | **P90** | **P95** |
| --- | --- | --- | --- | --- | --- | --- | --- | --- | --- | --- | --- | --- | --- |
| 6 | 25 | 1.76 | 0.12 | 0.591 | 1.785 | 0.083 | 1.49 | 1.56 | 1.66 | 1.78 | 1.91 | 2.01 | 2.08 |
| 7 | 32 | 1.88 | 0.19 | 0.591 | 1.847 | 0.083 | 1.54 | 1.61 | 1.72 | 1.85 | 1.97 | 2.08 | 2.15 |
| 8 | 34 | 1.92 | 0.13 | 0.591 | 1.911 | 0.083 | 1.60 | 1.67 | 1.78 | 1.91 | 2.04 | 2.16 | 2.23 |
| 9 | 4 | 1.98 | 0.21 | 0.591 | 1.977 | 0.083 | 1.65 | 1.72 | 1.84 | 1.98 | 2.11 | 2.23 | 2.30 |
| 10 | 43 | 2.04 | 0.14 | 0.591 | 2.046 | 0.083 | 1.71 | 1.78 | 1.91 | 2.05 | 2.18 | 2.31 | 2.38 |
| 11 | 68 | 2.10 | 0.17 | 0.591 | 2.117 | 0.083 | 1.77 | 1.85 | 1.97 | 2.12 | 2.26 | 2.39 | 2.47 |
| 12 | 58 | 2.21 | 0.16 | 0.591 | 2.191 | 0.083 | 1.83 | 1.91 | 2.04 | 2.19 | 2.34 | 2.47 | 2.55 |
| 13 | 44 | 2.30 | 0.26 | 0.591 | 2.267 | 0.083 | 1.89 | 1.98 | 2.11 | 2.27 | 2.42 | 2.56 | 2.64 |
| 14 | 17 | 2.35 | 0.17 | 0.591 | 2.346 | 0.083 | 1.96 | 2.05 | 2.19 | 2.35 | 2.50 | 2.65 | 2.73 |
| 15 | 26 | 2.39 | 0.22 | 0.591 | 2.428 | 0.083 | 2.03 | 2.12 | 2.26 | 2.43 | 2.59 | 2.74 | 2.83 |

**Table S14.** Age (years), number of individuals (N), mean, standard deviation (S.D.), L, M, and S values, and percentiles of the proximal phalanx of the fourth digit according to bone age in females.

| **Age** | **N** | **Mean** | **S.D.** | **L** | **M** | **S** | **P5** | **P10** | **P25** | **P50** | **P75** | **P90** | **P95** |
| --- | --- | --- | --- | --- | --- | --- | --- | --- | --- | --- | --- | --- | --- |
| 6 | 25 | 2.79 | 0.18 | 1.412 | 2.822 | 0.071 | 2.36 | 2.46 | 2.63 | 2.82 | 3.01 | 3.18 | 3.29 |
| 7 | 32 | 2.93 | 0.22 | 1.412 | 2.920 | 0.071 | 2.44 | 2.55 | 2.72 | 2.92 | 3.12 | 3.29 | 3.40 |
| 8 | 34 | 3.05 | 0.24 | 1.412 | 3.021 | 0.071 | 2.52 | 2.63 | 2.82 | 3.02 | 3.22 | 3.41 | 3.52 |
| 9 | 4 | 3.00 | 0.18 | 1.412 | 3.125 | 0.071 | 2.61 | 2.72 | 2.91 | 3.13 | 3.34 | 3.53 | 3.64 |
| 10 | 43 | 3.22 | 0.20 | 1.412 | 3.233 | 0.071 | 2.70 | 2.82 | 3.02 | 3.23 | 3.45 | 3.65 | 3.77 |
| 11 | 68 | 3.32 | 0.23 | 1.412 | 3.345 | 0.071 | 2.80 | 2.92 | 3.12 | 3.35 | 3.57 | 3.77 | 3.90 |
| 12 | 58 | 3.48 | 0.23 | 1.412 | 3.461 | 0.071 | 2.89 | 3.02 | 3.23 | 3.46 | 3.69 | 3.90 | 4.03 |
| 13 | 44 | 3.60 | 0.31 | 1.412 | 3.581 | 0.071 | 2.99 | 3.12 | 3.34 | 3.58 | 3.82 | 4.04 | 4.17 |
| 14 | 17 | 3.73 | 0.23 | 1.412 | 3.705 | 0.071 | 3.10 | 3.23 | 3.45 | 3.70 | 3.95 | 4.18 | 4.31 |
| 15 | 26 | 3.77 | 0.30 | 1.412 | 3.833 | 0.071 | 3.20 | 3.34 | 3.57 | 3.83 | 4.09 | 4.32 | 4.46 |

**Table S15.** Age (years), number of individuals (N), mean, standard deviation (S.D.), L, M, and S values, and percentiles of the fourth metacarpal according to bone age in females.

| **Age** | **N** | **Mean** | **S.D.** | **L** | **M** | **S** | **P5** | **P10** | **P25** | **P50** | **P75** | **P90** | **P95** |
| --- | --- | --- | --- | --- | --- | --- | --- | --- | --- | --- | --- | --- | --- |
| 6 | 25 | 3.91 | 0.29 | 1.135 | 3.969 | 0.072 | 3.32 | 3.46 | 3.70 | 3.97 | 4.24 | 4.48 | 4.62 |
| 7 | 32 | 4.16 | 0.30 | 1.135 | 4.104 | 0.072 | 3.43 | 3.58 | 3.83 | 4.10 | 4.38 | 4.63 | 4.78 |
| 8 | 34 | 4.25 | 0.31 | 1.135 | 4.244 | 0.072 | 3.55 | 3.70 | 3.96 | 4.24 | 4.53 | 4.79 | 4.94 |
| 9 | 4 | 4.38 | 0.33 | 1.135 | 4.389 | 0.072 | 3.67 | 3.83 | 4.09 | 4.39 | 4.69 | 4.95 | 5.11 |
| 10 | 43 | 4.48 | 0.28 | 1.135 | 4.539 | 0.072 | 3.79 | 3.96 | 4.23 | 4.54 | 4.85 | 5.12 | 5.29 |
| 11 | 68 | 4.68 | 0.31 | 1.135 | 4.694 | 0.072 | 3.92 | 4.09 | 4.38 | 4.69 | 5.01 | 5.30 | 5.47 |
| 12 | 58 | 4.92 | 0.40 | 1.135 | 4.854 | 0.072 | 4.06 | 4.23 | 4.53 | 4.85 | 5.18 | 5.48 | 5.65 |
| 13 | 44 | 5.04 | 0.43 | 1.135 | 5.019 | 0.072 | 4.19 | 4.38 | 4.68 | 5.02 | 5.36 | 5.66 | 5.84 |
| 14 | 17 | 5.20 | 0.30 | 1.135 | 5.190 | 0.072 | 4.34 | 4.53 | 4.84 | 5.19 | 5.54 | 5.86 | 6.04 |
| 15 | 26 | 5.27 | 0.31 | 1.135 | 5.367 | 0.072 | 4.48 | 4.68 | 5.00 | 5.37 | 5.73 | 6.05 | 6.25 |

**Table S16.** Age (years), number of individuals (N), mean, standard deviation (S.D.), L, M, and S values, and percentiles of the distal phalanx of the fifth digit according to bone age in females.

| **Age** | **N** | **Mean** | **S.D.** | **L** | **M** | **S** | **P5** | **P10** | **P25** | **P50** | **P75** | **P90** | **P95** |
| --- | --- | --- | --- | --- | --- | --- | --- | --- | --- | --- | --- | --- | --- |
| 6 | 25 | 1.04 | 0.08 | 1.189 | 1.054 | 0.094 | 0.88 | 0.92 | 0.98 | 1.05 | 1.12 | 1.19 | 1.23 |
| 7 | 32 | 1.10 | 0.11 | 1.189 | 1.095 | 0.094 | 0.92 | 0.95 | 1.02 | 1.10 | 1.17 | 1.24 | 1.28 |
| 8 | 34 | 1.13 | 0.11 | 1.189 | 1.138 | 0.094 | 0.95 | 0.99 | 1.06 | 1.14 | 1.21 | 1.28 | 1.32 |
| 9 | 4 | 1.21 | 0.11 | 1.189 | 1.182 | 0.094 | 0.99 | 1.03 | 1.10 | 1.18 | 1.26 | 1.33 | 1.38 |
| 10 | 43 | 1.23 | 0.12 | 1.189 | 1.226 | 0.094 | 1.02 | 1.07 | 1.14 | 1.23 | 1.31 | 1.38 | 1.43 |
| 11 | 68 | 1.26 | 0.12 | 1.189 | 1.271 | 0.094 | 1.06 | 1.11 | 1.19 | 1.27 | 1.36 | 1.43 | 1.48 |
| 12 | 58 | 1.33 | 0.12 | 1.189 | 1.317 | 0.094 | 1.10 | 1.15 | 1.23 | 1.32 | 1.41 | 1.49 | 1.53 |
| 13 | 44 | 1.38 | 0.15 | 1.189 | 1.363 | 0.094 | 1.14 | 1.19 | 1.27 | 1.36 | 1.46 | 1.54 | 1.59 |
| 14 | 17 | 1.41 | 0.10 | 1.189 | 1.409 | 0.094 | 1.18 | 1.23 | 1.31 | 1.41 | 1.50 | 1.59 | 1.64 |
| 15 | 26 | 1.43 | 0.13 | 1.189 | 1.456 | 0.094 | 1.22 | 1.27 | 1.36 | 1.46 | 1.55 | 1.64 | 1.70 |

**Table S17.** Age (years), number of individuals (N), mean, standard deviation (S.D.), L, M, and S values, and percentiles of the middle phalanx of the fifth digit according to bone age in females.

| **Age** | **N** | **Mean** | **S.D.** | **L** | **M** | **S** | **P5** | **P10** | **P25** | **P50** | **P75** | **P90** | **P95** |
| --- | --- | --- | --- | --- | --- | --- | --- | --- | --- | --- | --- | --- | --- |
| 6 | 25 | 1.15 | 0.14 | 2.120 | 1.184 | 0.119 | 0.99 | 1.03 | 1.10 | 1.18 | 1.26 | 1.34 | 1.38 |
| 7 | 32 | 1.23 | 0.17 | 2.120 | 1.228 | 0.119 | 1.03 | 1.07 | 1.14 | 1.23 | 1.31 | 1.38 | 1.43 |
| 8 | 34 | 1.26 | 0.16 | 2.120 | 1.273 | 0.119 | 1.06 | 1.11 | 1.19 | 1.27 | 1.36 | 1.44 | 1.48 |
| 9 | 4 | 1.33 | 0.15 | 2.120 | 1.320 | 0.119 | 1.10 | 1.15 | 1.23 | 1.32 | 1.41 | 1.49 | 1.54 |
| 10 | 43 | 1.36 | 0.16 | 2.120 | 1.368 | 0.119 | 1.14 | 1.19 | 1.28 | 1.37 | 1.46 | 1.54 | 1.59 |
| 11 | 68 | 1.40 | 0.17 | 2.120 | 1.418 | 0.119 | 1.19 | 1.24 | 1.32 | 1.42 | 1.51 | 1.60 | 1.65 |
| 12 | 58 | 1.46 | 0.17 | 2.120 | 1.470 | 0.119 | 1.23 | 1.28 | 1.37 | 1.47 | 1.57 | 1.66 | 1.71 |
| 13 | 44 | 1.53 | 0.21 | 2.120 | 1.525 | 0.119 | 1.27 | 1.33 | 1.42 | 1.52 | 1.63 | 1.72 | 1.78 |
| 14 | 17 | 1.57 | 0.17 | 2.120 | 1.581 | 0.119 | 1.32 | 1.38 | 1.47 | 1.58 | 1.69 | 1.78 | 1.84 |
| 15 | 26 | 1.60 | 0.21 | 2.120 | 1.639 | 0.119 | 1.37 | 1.43 | 1.53 | 1.64 | 1.75 | 1.85 | 1.91 |

**Table S18.** Age (years), number of individuals (N), mean, standard deviation (S.D.), L, M, and S values, and percentiles of the proximal phalanx of the fifth digit according to bone age in females.

| **Age** | **N** | **Mean** | **S.D.** | **L** | **M** | **S** | **P5** | **P10** | **P25** | **P50** | **P75** | **P90** | **P95** |
| --- | --- | --- | --- | --- | --- | --- | --- | --- | --- | --- | --- | --- | --- |
| 6 | 25 | 2.14 | 0.12 | 0.819 | 2.154 | 0.085 | 1.80 | 1.88 | 2.01 | 2.15 | 2.30 | 2.43 | 2.51 |
| 7 | 32 | 2.24 | 0.17 | 0.819 | 2.233 | 0.085 | 1.87 | 1.95 | 2.08 | 2.23 | 2.38 | 2.52 | 2.60 |
| 8 | 34 | 2.32 | 0.18 | 0.819 | 2.314 | 0.085 | 1.93 | 2.02 | 2.16 | 2.31 | 2.47 | 2.61 | 2.70 |
| 9 | 4 | 2.35 | 0.24 | 0.819 | 2.399 | 0.085 | 2.00 | 2.09 | 2.24 | 2.40 | 2.56 | 2.71 | 2.79 |
| 10 | 43 | 2.47 | 0.17 | 0.819 | 2.487 | 0.085 | 2.08 | 2.17 | 2.32 | 2.49 | 2.65 | 2.81 | 2.90 |
| 11 | 68 | 2.59 | 0.27 | 0.819 | 2.577 | 0.085 | 2.15 | 2.25 | 2.40 | 2.58 | 2.75 | 2.91 | 3.00 |
| 12 | 58 | 2.70 | 0.19 | 0.819 | 2.669 | 0.085 | 2.23 | 2.33 | 2.49 | 2.67 | 2.85 | 3.01 | 3.11 |
| 13 | 44 | 2.78 | 0.32 | 0.819 | 2.764 | 0.085 | 2.31 | 2.41 | 2.58 | 2.76 | 2.95 | 3.12 | 3.22 |
| 14 | 17 | 2.86 | 0.20 | 0.819 | 2.862 | 0.085 | 2.39 | 2.49 | 2.67 | 2.86 | 3.05 | 3.23 | 3.33 |
| 15 | 26 | 2.90 | 0.26 | 0.819 | 2.962 | 0.085 | 2.47 | 2.58 | 2.76 | 2.96 | 3.16 | 3.34 | 3.45 |

**Table S19.** Age (years), number of individuals (N), mean, standard deviation (S.D.), L, M, and S values, and percentiles of the fifth metacarpal according to bone age in females.

| **Age** | **N** | **Mean** | **S.D.** | **L** | **M** | **S** | **P5** | **P10** | **P25** | **P50** | **P75** | **P90** | **P95** |
| --- | --- | --- | --- | --- | --- | --- | --- | --- | --- | --- | --- | --- | --- |
| 6 | 25 | 3.56 | 0.20 | 1.746 | 3.594 | 0.071 | 3.00 | 3.13 | 3.35 | 3.59 | 3.84 | 4.05 | 4.18 |
| 7 | 32 | 3.78 | 0.28 | 1.746 | 3.747 | 0.071 | 3.13 | 3.27 | 3.49 | 3.75 | 4.00 | 4.23 | 4.36 |
| 8 | 34 | 3.90 | 0.29 | 1.746 | 3.895 | 0.071 | 3.25 | 3.40 | 3.63 | 3.90 | 4.16 | 4.39 | 4.54 |
| 9 | 4 | 4.03 | 0.30 | 1.746 | 4.038 | 0.071 | 3.37 | 3.52 | 3.77 | 4.04 | 4.31 | 4.56 | 4.70 |
| 10 | 43 | 4.16 | 0.26 | 1.746 | 4.184 | 0.071 | 3.50 | 3.65 | 3.90 | 4.18 | 4.47 | 4.72 | 4.87 |
| 11 | 68 | 4.31 | 0.29 | 1.746 | 4.341 | 0.071 | 3.63 | 3.78 | 4.05 | 4.34 | 4.63 | 4.90 | 5.06 |
| 12 | 58 | 4.52 | 0.32 | 1.746 | 4.502 | 0.071 | 3.76 | 3.92 | 4.20 | 4.50 | 4.81 | 5.08 | 5.24 |
| 13 | 44 | 4.69 | 0.41 | 1.746 | 4.646 | 0.071 | 3.88 | 4.05 | 4.33 | 4.65 | 4.96 | 5.24 | 5.41 |
| 14 | 17 | 4.66 | 0.47 | 1.746 | 4.761 | 0.071 | 3.98 | 4.15 | 4.44 | 4.76 | 5.08 | 5.37 | 5.54 |
| 15 | 26 | 4.82 | 0.31 | 1.746 | 4.861 | 0.071 | 4.06 | 4.24 | 4.53 | 4.86 | 5.19 | 5.48 | 5.66 |

**Male – Bone Age**

**Table S20.** Age (years), number of individuals (N), mean, standard deviation (S.D.), L, M, and S values, and percentiles of the distal phalanx of the first digit according to bone age in males.

| **Age** | **N** | **Mean** | **S.D.** | **L** | **M** | **S** | **P5** | **P10** | **P25** | **P50** | **P75** | **P90** | **P95** |
| --- | --- | --- | --- | --- | --- | --- | --- | --- | --- | --- | --- | --- | --- |
| 6 | 29 | 1.50 | 0.12 | -0.209 | 1.506 | 0.092 | 1.26 | 1.31 | 1.40 | 1.51 | 1.61 | 1.70 | 1.75 |
| 7 | 28 | 1.62 | 0.18 | -0.209 | 1.560 | 0.092 | 1.30 | 1.36 | 1.45 | 1.56 | 1.67 | 1.76 | 1.82 |
| 8 | 40 | 1.62 | 0.14 | -0.209 | 1.616 | 0.092 | 1.35 | 1.41 | 1.51 | 1.62 | 1.72 | 1.82 | 1.88 |
| 9 | 40 | 1.68 | 0.17 | -0.209 | 1.674 | 0.092 | 1.40 | 1.46 | 1.56 | 1.67 | 1.79 | 1.89 | 1.95 |
| 10 | 53 | 1.74 | 0.16 | -0.209 | 1.735 | 0.092 | 1.45 | 1.51 | 1.62 | 1.74 | 1.85 | 1.96 | 2.02 |
| 11 | 56 | 1.79 | 0.15 | -0.209 | 1.800 | 0.092 | 1.50 | 1.57 | 1.68 | 1.80 | 1.92 | 2.03 | 2.10 |
| 12 | 30 | 1.88 | 0.16 | -0.209 | 1.869 | 0.092 | 1.56 | 1.63 | 1.74 | 1.87 | 2.00 | 2.11 | 2.18 |
| 13 | 62 | 1.94 | 0.19 | -0.209 | 1.941 | 0.092 | 1.62 | 1.69 | 1.81 | 1.94 | 2.07 | 2.19 | 2.26 |
| 14 | 60 | 2.04 | 0.18 | -0.209 | 2.017 | 0.092 | 1.69 | 1.76 | 1.88 | 2.02 | 2.15 | 2.28 | 2.35 |
| 15 | 47 | 2.11 | 0.19 | -0.209 | 2.097 | 0.092 | 1.75 | 1.83 | 1.96 | 2.10 | 2.24 | 2.37 | 2.44 |

**Table S21.** Age (years), number of individuals (N), mean, standard deviation (S.D.), L, M, and S values, and percentiles of the proximal phalanx of the first digit according to bone age in males.

| **Age** | **N** | **Mean** | **S.D.** | **L** | **M** | **S** | **P5** | **P10** | **P25** | **P50** | **P75** | **P90** | **P95** |
| --- | --- | --- | --- | --- | --- | --- | --- | --- | --- | --- | --- | --- | --- |
| 6 | 29 | 1.97 | 0.16 | -0.537 | 1.979 | 0.076 | 1.65 | 1.73 | 1.85 | 1.98 | 2.11 | 2.23 | 2.30 |
| 7 | 28 | 2.09 | 0.14 | -0.537 | 2.049 | 0.076 | 1.71 | 1.79 | 1.91 | 2.05 | 2.19 | 2.31 | 2.39 |
| 8 | 40 | 2.14 | 0.15 | -0.537 | 2.114 | 0.076 | 1.77 | 1.84 | 1.97 | 2.11 | 2.26 | 2.38 | 2.46 |
| 9 | 40 | 2.17 | 0.17 | -0.537 | 2.178 | 0.076 | 1.82 | 1.90 | 2.03 | 2.18 | 2.32 | 2.46 | 2.54 |
| 10 | 53 | 2.27 | 0.17 | -0.537 | 2.258 | 0.076 | 1.89 | 1.97 | 2.11 | 2.26 | 2.41 | 2.55 | 2.63 |
| 11 | 56 | 2.35 | 0.19 | -0.537 | 2.364 | 0.076 | 1.98 | 2.06 | 2.20 | 2.36 | 2.52 | 2.67 | 2.75 |
| 12 | 30 | 2.55 | 0.18 | -0.537 | 2.498 | 0.076 | 2.09 | 2.18 | 2.33 | 2.50 | 2.67 | 2.82 | 2.91 |
| 13 | 62 | 2.66 | 0.22 | -0.537 | 2.651 | 0.076 | 2.21 | 2.31 | 2.47 | 2.65 | 2.83 | 2.99 | 3.09 |
| 14 | 60 | 2.84 | 0.26 | -0.537 | 2.816 | 0.076 | 2.35 | 2.46 | 2.63 | 2.82 | 3.01 | 3.18 | 3.28 |
| 15 | 47 | 3.00 | 0.18 | -0.537 | 2.994 | 0.076 | 2.50 | 2.61 | 2.79 | 2.99 | 3.20 | 3.38 | 3.49 |

**Table S22.** Age (years), number of individuals (N), mean, standard deviation (S.D.), L, M, and S values, and percentiles of the first metacarpal according to bone age in males.

| **Age** | **N** | **Mean** | **S.D.** | **L** | **M** | **S** | **P5** | **P10** | **P25** | **P50** | **P75** | **P90** | **P95** |
| --- | --- | --- | --- | --- | --- | --- | --- | --- | --- | --- | --- | --- | --- |
| 6 | 29 | 2.99 | 0.20 | -0.419 | 2.994 | 0.071 | 2.50 | 2.61 | 2.79 | 2.99 | 3.20 | 3.38 | 3.49 |
| 7 | 28 | 3.14 | 0.22 | -0.419 | 3.117 | 0.071 | 2.60 | 2.72 | 2.91 | 3.12 | 3.33 | 3.52 | 3.63 |
| 8 | 40 | 3.29 | 0.23 | -0.419 | 3.242 | 0.071 | 2.71 | 2.83 | 3.02 | 3.24 | 3.46 | 3.66 | 3.78 |
| 9 | 40 | 3.36 | 0.30 | -0.419 | 3.371 | 0.071 | 2.82 | 2.94 | 3.14 | 3.37 | 3.60 | 3.80 | 3.93 |
| 10 | 53 | 3.52 | 0.27 | -0.419 | 3.513 | 0.071 | 2.94 | 3.06 | 3.28 | 3.51 | 3.75 | 3.96 | 4.09 |
| 11 | 56 | 3.69 | 0.25 | -0.419 | 3.674 | 0.071 | 3.07 | 3.20 | 3.43 | 3.67 | 3.92 | 4.15 | 4.28 |
| 12 | 30 | 3.86 | 0.28 | -0.419 | 3.858 | 0.071 | 3.22 | 3.36 | 3.60 | 3.86 | 4.12 | 4.35 | 4.49 |
| 13 | 62 | 4.05 | 0.28 | -0.419 | 4.069 | 0.071 | 3.40 | 3.55 | 3.79 | 4.07 | 4.34 | 4.59 | 4.74 |
| 14 | 60 | 4.35 | 0.35 | -0.419 | 4.309 | 0.071 | 3.60 | 3.76 | 4.02 | 4.31 | 4.60 | 4.86 | 5.02 |
| 15 | 47 | 4.59 | 0.27 | -0.419 | 4.573 | 0.071 | 3.82 | 3.99 | 4.26 | 4.57 | 4.88 | 5.16 | 5.32 |

**Table S23.** Age (years), number of individuals (N), mean, standard deviation (S.D.), L, M, and S values, and percentiles of the distal phalanx of the second digit according to bone age in males.

| **Age** | **N** | **Mean** | **S.D.** | **L** | **M** | **S** | **P5** | **P10** | **P25** | **P50** | **P75** | **P90** | **P95** |
| --- | --- | --- | --- | --- | --- | --- | --- | --- | --- | --- | --- | --- | --- |
| 6 | 29 | 1.10 | 0.09 | 0.050 | 1.098 | 0.095 | 0.92 | 0.96 | 1.02 | 1.10 | 1.17 | 1.24 | 1.28 |
| 7 | 28 | 1.16 | 0.13 | 0.050 | 1.134 | 0.095 | 0.95 | 0.99 | 1.06 | 1.13 | 1.21 | 1.28 | 1.32 |
| 8 | 40 | 1.16 | 0.10 | 0.050 | 1.172 | 0.095 | 0.98 | 1.02 | 1.09 | 1.17 | 1.25 | 1.32 | 1.36 |
| 9 | 40 | 1.24 | 0.14 | 0.050 | 1.214 | 0.095 | 1.01 | 1.06 | 1.13 | 1.21 | 1.30 | 1.37 | 1.41 |
| 10 | 53 | 1.26 | 0.12 | 0.050 | 1.262 | 0.095 | 1.05 | 1.10 | 1.18 | 1.26 | 1.35 | 1.42 | 1.47 |
| 11 | 56 | 1.30 | 0.12 | 0.050 | 1.320 | 0.095 | 1.10 | 1.15 | 1.23 | 1.32 | 1.41 | 1.49 | 1.54 |
| 12 | 30 | 1.41 | 0.11 | 0.050 | 1.389 | 0.095 | 1.16 | 1.21 | 1.30 | 1.39 | 1.48 | 1.57 | 1.62 |
| 13 | 62 | 1.48 | 0.14 | 0.050 | 1.466 | 0.095 | 1.23 | 1.28 | 1.37 | 1.47 | 1.57 | 1.65 | 1.71 |
| 14 | 60 | 1.57 | 0.14 | 0.050 | 1.547 | 0.095 | 1.29 | 1.35 | 1.44 | 1.55 | 1.65 | 1.75 | 1.80 |
| 15 | 47 | 1.63 | 0.16 | 0.050 | 1.631 | 0.095 | 1.36 | 1.42 | 1.52 | 1.63 | 1.74 | 1.84 | 1.90 |

**Table S24.** Age (years), number of individuals (N), mean, standard deviation (S.D.), L, M, and S values, and percentiles of the middle phalanx of the second digit according to bone age in males.

| **Age** | **N** | **Mean** | **S.D.** | **L** | **M** | **S** | **P5** | **P10** | **P25** | **P50** | **P75** | **P90** | **P95** |
| --- | --- | --- | --- | --- | --- | --- | --- | --- | --- | --- | --- | --- | --- |
| 6 | 29 | 1.48 | 0.14 | -6.499 | 1.528 | 0.196 | 1.28 | 1.33 | 1.42 | 1.53 | 1.63 | 1.72 | 1.78 |
| 7 | 28 | 1.60 | 0.15 | -6.499 | 1.594 | 0.196 | 1.33 | 1.39 | 1.49 | 1.59 | 1.70 | 1.80 | 1.86 |
| 8 | 40 | 1.62 | 0.11 | -6.499 | 1.664 | 0.196 | 1.39 | 1.45 | 1.55 | 1.66 | 1.78 | 1.88 | 1.94 |
| 9 | 40 | 1.66 | 0.13 | -6.499 | 1.736 | 0.196 | 1.45 | 1.51 | 1.62 | 1.74 | 1.85 | 1.96 | 2.02 |
| 10 | 53 | 1.72 | 0.17 | -6.499 | 1.812 | 0.196 | 1.51 | 1.58 | 1.69 | 1.81 | 1.93 | 2.04 | 2.11 |
| 11 | 56 | 2.07 | 2.13 | -6.499 | 1.892 | 0.196 | 1.58 | 1.65 | 1.76 | 1.89 | 2.02 | 2.13 | 2.20 |
| 12 | 30 | 1.91 | 0.12 | -6.499 | 1.974 | 0.196 | 1.65 | 1.72 | 1.84 | 1.97 | 2.11 | 2.23 | 2.30 |
| 13 | 62 | 1.98 | 0.18 | -6.499 | 2.061 | 0.196 | 1.72 | 1.80 | 1.92 | 2.06 | 2.20 | 2.33 | 2.40 |
| 14 | 60 | 2.09 | 0.19 | -6.499 | 2.151 | 0.196 | 1.80 | 1.88 | 2.01 | 2.15 | 2.30 | 2.43 | 2.51 |
| 15 | 47 | 2.21 | 0.17 | -6.499 | 2.246 | 0.196 | 1.88 | 1.96 | 2.09 | 2.25 | 2.40 | 2.53 | 2.62 |

**Table S25.** Age (years), number of individuals (N), mean, standard deviation (S.D.), L, M, and S values, and percentiles of the proximal phalanx of the second digit according to bone age in males.

| **Age** | **N** | **Mean** | **S.D.** | **L** | **M** | **S** | **P5** | **P10** | **P25** | **P50** | **P75** | **P90** | **P95** |
| --- | --- | --- | --- | --- | --- | --- | --- | --- | --- | --- | --- | --- | --- |
| 6 | 29 | 2.64 | 0.24 | 1.070 | 2.677 | 0.074 | 2.24 | 2.33 | 2.50 | 2.68 | 2.86 | 3.02 | 3.12 |
| 7 | 28 | 2.79 | 0.22 | 1.070 | 2.771 | 0.074 | 2.32 | 2.42 | 2.58 | 2.77 | 2.96 | 3.13 | 3.23 |
| 8 | 40 | 2.91 | 0.18 | 1.070 | 2.860 | 0.074 | 2.39 | 2.49 | 2.67 | 2.86 | 3.05 | 3.23 | 3.33 |
| 9 | 40 | 2.92 | 0.23 | 1.070 | 2.945 | 0.074 | 2.46 | 2.57 | 2.75 | 2.94 | 3.14 | 3.32 | 3.43 |
| 10 | 53 | 3.03 | 0.23 | 1.070 | 3.041 | 0.074 | 2.54 | 2.65 | 2.84 | 3.04 | 3.25 | 3.43 | 3.54 |
| 11 | 56 | 3.15 | 0.22 | 1.070 | 3.160 | 0.074 | 2.64 | 2.76 | 2.95 | 3.16 | 3.37 | 3.57 | 3.68 |
| 12 | 30 | 3.34 | 0.23 | 1.070 | 3.307 | 0.074 | 2.76 | 2.88 | 3.08 | 3.31 | 3.53 | 3.73 | 3.85 |
| 13 | 62 | 3.46 | 0.27 | 1.070 | 3.478 | 0.074 | 2.91 | 3.03 | 3.24 | 3.48 | 3.71 | 3.92 | 4.05 |
| 14 | 60 | 3.69 | 0.32 | 1.070 | 3.666 | 0.074 | 3.06 | 3.20 | 3.42 | 3.67 | 3.91 | 4.14 | 4.27 |
| 15 | 47 | 3.86 | 0.22 | 1.070 | 3.865 | 0.074 | 3.23 | 3.37 | 3.60 | 3.87 | 4.13 | 4.36 | 4.50 |

**Table S26.** Age (years), number of individuals (N), mean, standard deviation (S.D.), L, M, and S values, and percentiles of the second metacarpal according to bone age in males.

| **Age** | **N** | **Mean** | **S.D.** | **L** | **M** | **S** | **P5** | **P10** | **P25** | **P50** | **P75** | **P90** | **P95** |
| --- | --- | --- | --- | --- | --- | --- | --- | --- | --- | --- | --- | --- | --- |
| 6 | 29 | 4.52 | 0.31 | -0.125 | 4.540 | 0.069 | 3.79 | 3.96 | 4.23 | 4.54 | 4.85 | 5.12 | 5.29 |
| 7 | 28 | 4.74 | 0.32 | -0.125 | 4.720 | 0.069 | 3.94 | 4.11 | 4.40 | 4.72 | 5.04 | 5.32 | 5.50 |
| 8 | 40 | 5.00 | 0.34 | -0.125 | 4.900 | 0.069 | 4.09 | 4.27 | 4.57 | 4.90 | 5.23 | 5.53 | 5.71 |
| 9 | 40 | 5.07 | 0.44 | -0.125 | 5.079 | 0.069 | 4.24 | 4.43 | 4.74 | 5.08 | 5.42 | 5.73 | 5.91 |
| 10 | 53 | 5.26 | 0.40 | -0.125 | 5.270 | 0.069 | 4.40 | 4.59 | 4.91 | 5.27 | 5.63 | 5.95 | 6.14 |
| 11 | 56 | 5.49 | 0.35 | -0.125 | 5.486 | 0.069 | 4.58 | 4.78 | 5.12 | 5.49 | 5.86 | 6.19 | 6.39 |
| 12 | 30 | 5.77 | 0.30 | -0.125 | 5.732 | 0.069 | 4.79 | 5.00 | 5.34 | 5.73 | 6.12 | 6.47 | 6.67 |
| 13 | 62 | 5.98 | 0.43 | -0.125 | 6.011 | 0.069 | 5.02 | 5.24 | 5.61 | 6.01 | 6.42 | 6.78 | 7.00 |
| 14 | 60 | 6.38 | 0.48 | -0.125 | 6.326 | 0.069 | 5.29 | 5.52 | 5.90 | 6.33 | 6.75 | 7.14 | 7.37 |
| 15 | 47 | 6.69 | 0.31 | -0.125 | 6.669 | 0.069 | 5.57 | 5.81 | 6.22 | 6.67 | 7.12 | 7.52 | 7.77 |

**Table S27.** Age (years), number of individuals (N), mean, standard deviation (S.D.), L, M, and S values, and percentiles of the distal phalanx of the third digit according to bone age in males.

| **Age** | **N** | **Mean** | **S.D.** | **L** | **M** | **S** | **P5** | **P10** | **P25** | **P50** | **P75** | **P90** | **P95** |
| --- | --- | --- | --- | --- | --- | --- | --- | --- | --- | --- | --- | --- | --- |
| 6 | 29 | 1.17 | 0.10 | 0.187 | 1.173 | 0.095 | 0.98 | 1.02 | 1.09 | 1.17 | 1.25 | 1.32 | 1.37 |
| 7 | 28 | 1.25 | 0.14 | 0.187 | 1.212 | 0.095 | 1.01 | 1.06 | 1.13 | 1.21 | 1.29 | 1.37 | 1.41 |
| 8 | 40 | 1.24 | 0.11 | 0.187 | 1.252 | 0.095 | 1.05 | 1.09 | 1.17 | 1.25 | 1.34 | 1.41 | 1.46 |
| 9 | 40 | 1.31 | 0.15 | 0.187 | 1.296 | 0.095 | 1.08 | 1.13 | 1.21 | 1.30 | 1.38 | 1.46 | 1.51 |
| 10 | 53 | 1.35 | 0.14 | 0.187 | 1.347 | 0.095 | 1.13 | 1.17 | 1.26 | 1.35 | 1.44 | 1.52 | 1.57 |
| 11 | 56 | 1.40 | 0.13 | 0.187 | 1.408 | 0.095 | 1.18 | 1.23 | 1.31 | 1.41 | 1.50 | 1.59 | 1.64 |
| 12 | 30 | 1.49 | 0.12 | 0.187 | 1.482 | 0.095 | 1.24 | 1.29 | 1.38 | 1.48 | 1.58 | 1.67 | 1.73 |
| 13 | 62 | 1.58 | 0.16 | 0.187 | 1.567 | 0.095 | 1.31 | 1.37 | 1.46 | 1.57 | 1.67 | 1.77 | 1.82 |
| 14 | 60 | 1.68 | 0.15 | 0.187 | 1.659 | 0.095 | 1.39 | 1.45 | 1.55 | 1.66 | 1.77 | 1.87 | 1.93 |
| 15 | 47 | 1.76 | 0.16 | 0.187 | 1.756 | 0.095 | 1.47 | 1.53 | 1.64 | 1.76 | 1.87 | 1.98 | 2.04 |

**Table S28.** Age (years), number of individuals (N), mean, standard deviation (S.D.), L, M, and S values, and percentiles of the middle phalanx of the third digit according to bone age in males.

| **Age** | **N** | **Mean** | **S.D.** | **L** | **M** | **S** | **P5** | **P10** | **P25** | **P50** | **P75** | **P90** | **P95** |
| --- | --- | --- | --- | --- | --- | --- | --- | --- | --- | --- | --- | --- | --- |
| 6 | 29 | 1.87 | 0.13 | 0.229 | 1.881 | 0.079 | 1.57 | 1.64 | 1.75 | 1.88 | 2.01 | 2.12 | 2.19 |
| 7 | 28 | 1.98 | 0.16 | 0.229 | 1.940 | 0.079 | 1.62 | 1.69 | 1.81 | 1.94 | 2.07 | 2.19 | 2.26 |
| 8 | 40 | 2.01 | 0.14 | 0.229 | 1.996 | 0.079 | 1.67 | 1.74 | 1.86 | 2.00 | 2.13 | 2.25 | 2.32 |
| 9 | 40 | 2.06 | 0.14 | 0.229 | 2.054 | 0.079 | 1.72 | 1.79 | 1.92 | 2.05 | 2.19 | 2.32 | 2.39 |
| 10 | 53 | 2.11 | 0.18 | 0.229 | 2.122 | 0.079 | 1.77 | 1.85 | 1.98 | 2.12 | 2.26 | 2.39 | 2.47 |
| 11 | 56 | 2.20 | 0.19 | 0.229 | 2.205 | 0.079 | 1.84 | 1.92 | 2.06 | 2.21 | 2.35 | 2.49 | 2.57 |
| 12 | 30 | 2.34 | 0.13 | 0.229 | 2.305 | 0.079 | 1.93 | 2.01 | 2.15 | 2.30 | 2.46 | 2.60 | 2.68 |
| 13 | 62 | 2.42 | 0.21 | 0.229 | 2.416 | 0.079 | 2.02 | 2.11 | 2.25 | 2.42 | 2.58 | 2.73 | 2.81 |
| 14 | 60 | 2.54 | 0.22 | 0.229 | 2.538 | 0.079 | 2.12 | 2.21 | 2.37 | 2.54 | 2.71 | 2.86 | 2.96 |
| 15 | 47 | 2.68 | 0.21 | 0.229 | 2.668 | 0.079 | 2.23 | 2.33 | 2.49 | 2.67 | 2.85 | 3.01 | 3.11 |

**Table S29.** Age (years), number of individuals (N), mean, standard deviation (S.D.), L, M, and S values, and percentiles of the proximal phalanx of the third digit according to bone age in males.

| **Age** | **N** | **Mean** | **S.D.** | **L** | **M** | **S** | **P5** | **P10** | **P25** | **P50** | **P75** | **P90** | **P95** |
| --- | --- | --- | --- | --- | --- | --- | --- | --- | --- | --- | --- | --- | --- |
| 6 | 29 | 3.00 | 0.18 | -0.302 | 3.018 | 0.070 | 2.52 | 2.63 | 2.81 | 3.02 | 3.22 | 3.40 | 3.51 |
| 7 | 28 | 3.14 | 0.26 | -0.302 | 3.116 | 0.070 | 2.60 | 2.72 | 2.91 | 3.12 | 3.33 | 3.52 | 3.63 |
| 8 | 40 | 3.28 | 0.20 | -0.302 | 3.211 | 0.070 | 2.68 | 2.80 | 2.99 | 3.21 | 3.43 | 3.62 | 3.74 |
| 9 | 40 | 3.27 | 0.23 | -0.302 | 3.302 | 0.070 | 2.76 | 2.88 | 3.08 | 3.30 | 3.52 | 3.73 | 3.85 |
| 10 | 53 | 3.41 | 0.24 | -0.302 | 3.405 | 0.070 | 2.84 | 2.97 | 3.17 | 3.40 | 3.63 | 3.84 | 3.96 |
| 11 | 56 | 3.53 | 0.23 | -0.302 | 3.529 | 0.070 | 2.95 | 3.08 | 3.29 | 3.53 | 3.77 | 3.98 | 4.11 |
| 12 | 30 | 3.71 | 0.25 | -0.302 | 3.678 | 0.070 | 3.07 | 3.21 | 3.43 | 3.68 | 3.93 | 4.15 | 4.28 |
| 13 | 62 | 3.85 | 0.31 | -0.302 | 3.853 | 0.070 | 3.22 | 3.36 | 3.59 | 3.85 | 4.11 | 4.35 | 4.49 |
| 14 | 60 | 4.08 | 0.33 | -0.302 | 4.053 | 0.070 | 3.39 | 3.53 | 3.78 | 4.05 | 4.33 | 4.57 | 4.72 |
| 15 | 47 | 4.29 | 0.23 | -0.302 | 4.273 | 0.070 | 3.57 | 3.73 | 3.98 | 4.27 | 4.56 | 4.82 | 4.98 |

**Table S30.** Age (years), number of individuals (N), mean, standard deviation (S.D.), L, M, and S values, and percentiles of the third metacarpal according to bone age in males.

| **Age** | **N** | **Mean** | **S.D.** | **L** | **M** | **S** | **P5** | **P10** | **P25** | **P50** | **P75** | **P90** | **P95** |
| --- | --- | --- | --- | --- | --- | --- | --- | --- | --- | --- | --- | --- | --- |
| 6 | 29 | 4.37 | 0.31 | -0.396 | 4.396 | 0.070 | 3.67 | 3.83 | 4.10 | 4.40 | 4.69 | 4.96 | 5.12 |
| 7 | 28 | 4.61 | 0.33 | -0.396 | 4.578 | 0.070 | 3.83 | 3.99 | 4.27 | 4.58 | 4.89 | 5.17 | 5.33 |
| 8 | 40 | 4.84 | 0.36 | -0.396 | 4.759 | 0.070 | 3.98 | 4.15 | 4.44 | 4.76 | 5.08 | 5.37 | 5.54 |
| 9 | 40 | 4.94 | 0.43 | -0.396 | 4.935 | 0.070 | 4.12 | 4.30 | 4.60 | 4.94 | 5.27 | 5.57 | 5.75 |
| 10 | 53 | 5.14 | 0.38 | -0.396 | 5.116 | 0.070 | 4.27 | 4.46 | 4.77 | 5.12 | 5.46 | 5.77 | 5.96 |
| 11 | 56 | 5.30 | 0.33 | -0.396 | 5.315 | 0.070 | 4.44 | 4.63 | 4.96 | 5.31 | 5.67 | 6.00 | 6.19 |
| 12 | 30 | 5.58 | 0.32 | -0.396 | 5.541 | 0.070 | 4.63 | 4.83 | 5.17 | 5.54 | 5.91 | 6.25 | 6.45 |
| 13 | 62 | 5.77 | 0.42 | -0.396 | 5.800 | 0.070 | 4.85 | 5.06 | 5.41 | 5.80 | 6.19 | 6.54 | 6.75 |
| 14 | 60 | 6.15 | 0.46 | -0.396 | 6.097 | 0.070 | 5.09 | 5.32 | 5.69 | 6.10 | 6.51 | 6.88 | 7.10 |
| 15 | 47 | 6.45 | 0.35 | -0.396 | 6.420 | 0.070 | 5.36 | 5.60 | 5.99 | 6.42 | 6.85 | 7.24 | 7.48 |

**Table S31.** Age (years), number of individuals (N), mean, standard deviation (S.D.), L, M, and S values, and percentiles of the distal phalanx of the fourth digit according to bone age in males.

| **Age** | **N** | **Mean** | **S.D.** | **L** | **M** | **S** | **P5** | **P10** | **P25** | **P50** | **P75** | **P90** | **P95** |
| --- | --- | --- | --- | --- | --- | --- | --- | --- | --- | --- | --- | --- | --- |
| 6 | 29 | 1.24 | 0.10 | -0.106 | 1.237 | 0.094 | 1.03 | 1.08 | 1.15 | 1.24 | 1.32 | 1.40 | 1.44 |
| 7 | 28 | 1.30 | 0.15 | -0.106 | 1.276 | 0.094 | 1.07 | 1.11 | 1.19 | 1.28 | 1.36 | 1.44 | 1.49 |
| 8 | 40 | 1.31 | 0.12 | -0.106 | 1.318 | 0.094 | 1.10 | 1.15 | 1.23 | 1.32 | 1.41 | 1.49 | 1.53 |
| 9 | 40 | 1.39 | 0.16 | -0.106 | 1.364 | 0.094 | 1.14 | 1.19 | 1.27 | 1.36 | 1.46 | 1.54 | 1.59 |
| 10 | 53 | 1.43 | 0.13 | -0.106 | 1.415 | 0.094 | 1.18 | 1.23 | 1.32 | 1.42 | 1.51 | 1.60 | 1.65 |
| 11 | 56 | 1.46 | 0.13 | -0.106 | 1.476 | 0.094 | 1.23 | 1.29 | 1.38 | 1.48 | 1.58 | 1.67 | 1.72 |
| 12 | 30 | 1.55 | 0.14 | -0.106 | 1.549 | 0.094 | 1.29 | 1.35 | 1.44 | 1.55 | 1.65 | 1.75 | 1.80 |
| 13 | 62 | 1.65 | 0.15 | -0.106 | 1.635 | 0.094 | 1.37 | 1.43 | 1.52 | 1.63 | 1.75 | 1.84 | 1.90 |
| 14 | 60 | 1.74 | 0.16 | -0.106 | 1.731 | 0.094 | 1.45 | 1.51 | 1.61 | 1.73 | 1.85 | 1.95 | 2.02 |
| 15 | 47 | 1.85 | 0.19 | -0.106 | 1.834 | 0.094 | 1.53 | 1.60 | 1.71 | 1.83 | 1.96 | 2.07 | 2.14 |

**Table S32.** Age (years), number of individuals (N), mean, standard deviation (S.D.), L, M, and S values, and percentiles of the middle phalanx of the fourth digit according to bone age in males.

| **Age** | **N** | **Mean** | **S.D.** | **L** | **M** | **S** | **P5** | **P10** | **P25** | **P50** | **P75** | **P90** | **P95** |
| --- | --- | --- | --- | --- | --- | --- | --- | --- | --- | --- | --- | --- | --- |
| 6 | 29 | 1.78 | 0.14 | 0.193 | 1.786 | 0.079 | 1.49 | 1.56 | 1.67 | 1.79 | 1.91 | 2.01 | 2.08 |
| 7 | 28 | 1.87 | 0.18 | 0.193 | 1.843 | 0.079 | 1.54 | 1.61 | 1.72 | 1.84 | 1.97 | 2.08 | 2.15 |
| 8 | 40 | 1.91 | 0.12 | 0.193 | 1.901 | 0.079 | 1.59 | 1.66 | 1.77 | 1.90 | 2.03 | 2.14 | 2.21 |
| 9 | 40 | 1.96 | 0.15 | 0.193 | 1.963 | 0.079 | 1.64 | 1.71 | 1.83 | 1.96 | 2.10 | 2.21 | 2.29 |
| 10 | 53 | 2.04 | 0.18 | 0.193 | 2.033 | 0.079 | 1.70 | 1.77 | 1.90 | 2.03 | 2.17 | 2.29 | 2.37 |
| 11 | 56 | 2.12 | 0.18 | 0.193 | 2.114 | 0.079 | 1.77 | 1.84 | 1.97 | 2.11 | 2.26 | 2.38 | 2.46 |
| 12 | 30 | 2.20 | 0.12 | 0.193 | 2.207 | 0.079 | 1.84 | 1.92 | 2.06 | 2.21 | 2.36 | 2.49 | 2.57 |
| 13 | 62 | 2.31 | 0.19 | 0.193 | 2.315 | 0.079 | 1.93 | 2.02 | 2.16 | 2.31 | 2.47 | 2.61 | 2.70 |
| 14 | 60 | 2.46 | 0.21 | 0.193 | 2.434 | 0.079 | 2.03 | 2.12 | 2.27 | 2.43 | 2.60 | 2.75 | 2.83 |
| 15 | 47 | 2.57 | 0.17 | 0.193 | 2.564 | 0.079 | 2.14 | 2.23 | 2.39 | 2.56 | 2.74 | 2.89 | 2.99 |

**Table S33.** Age (years), number of individuals (N), mean, standard deviation (S.D.), L, M, and S values, and percentiles of the proximal phalanx of the fourth digit according to bone age in males.

| **Age** | **N** | **Mean** | **S.D.** | **L** | **M** | **S** | **P5** | **P10** | **P25** | **P50** | **P75** | **P90** | **P95** |
| --- | --- | --- | --- | --- | --- | --- | --- | --- | --- | --- | --- | --- | --- |
| 6 | 29 | 2.83 | 0.18 | -2.522 | 2.786 | 0.074 | 2.33 | 2.43 | 2.60 | 2.79 | 2.97 | 3.14 | 3.24 |
| 7 | 28 | 2.96 | 0.23 | -2.522 | 2.891 | 0.074 | 2.42 | 2.52 | 2.70 | 2.89 | 3.09 | 3.26 | 3.37 |
| 8 | 40 | 3.07 | 0.18 | -2.522 | 3.001 | 0.074 | 2.51 | 2.62 | 2.80 | 3.00 | 3.20 | 3.39 | 3.49 |
| 9 | 40 | 3.09 | 0.21 | -2.522 | 3.115 | 0.074 | 2.60 | 2.72 | 2.91 | 3.12 | 3.33 | 3.51 | 3.63 |
| 10 | 53 | 3.41 | 1.37 | -2.522 | 3.234 | 0.074 | 2.70 | 2.82 | 3.02 | 3.23 | 3.45 | 3.65 | 3.77 |
| 11 | 56 | 3.34 | 0.22 | -2.522 | 3.357 | 0.074 | 2.80 | 2.93 | 3.13 | 3.36 | 3.58 | 3.79 | 3.91 |
| 12 | 30 | 3.50 | 0.22 | -2.522 | 3.486 | 0.074 | 2.91 | 3.04 | 3.25 | 3.49 | 3.72 | 3.93 | 4.06 |
| 13 | 62 | 3.60 | 0.28 | -2.522 | 3.619 | 0.074 | 3.02 | 3.16 | 3.38 | 3.62 | 3.86 | 4.08 | 4.21 |
| 14 | 60 | 3.84 | 0.32 | -2.522 | 3.758 | 0.074 | 3.14 | 3.28 | 3.50 | 3.76 | 4.01 | 4.24 | 4.38 |
| 15 | 47 | 4.01 | 0.22 | -2.522 | 3.903 | 0.074 | 3.26 | 3.40 | 3.64 | 3.90 | 4.17 | 4.40 | 4.54 |

**Table S34.** Age (years), number of individuals (N), mean, standard deviation (S.D.), L, M, and S values, and percentiles of the fourth metacarpal according to bone age in males.

| **Age** | **N** | **Mean** | **S.D.** | **L** | **M** | **S** | **P5** | **P10** | **P25** | **P50** | **P75** | **P90** | **P95** |
| --- | --- | --- | --- | --- | --- | --- | --- | --- | --- | --- | --- | --- | --- |
| 6 | 29 | 3.84 | 0.32 | -0.200 | 3.855 | 0.072 | 3.22 | 3.36 | 3.59 | 3.85 | 4.11 | 4.35 | 4.49 |
| 7 | 28 | 4.04 | 0.28 | -0.200 | 4.008 | 0.072 | 3.35 | 3.49 | 3.74 | 4.01 | 4.28 | 4.52 | 4.67 |
| 8 | 40 | 4.23 | 0.31 | -0.200 | 4.162 | 0.072 | 3.48 | 3.63 | 3.88 | 4.16 | 4.44 | 4.70 | 4.85 |
| 9 | 40 | 4.31 | 0.34 | -0.200 | 4.317 | 0.072 | 3.61 | 3.76 | 4.03 | 4.32 | 4.61 | 4.87 | 5.03 |
| 10 | 53 | 4.51 | 0.35 | -0.200 | 4.481 | 0.072 | 3.74 | 3.91 | 4.18 | 4.48 | 4.78 | 5.06 | 5.22 |
| 11 | 56 | 4.64 | 0.29 | -0.200 | 4.664 | 0.072 | 3.90 | 4.07 | 4.35 | 4.66 | 4.98 | 5.26 | 5.43 |
| 12 | 30 | 4.89 | 0.30 | -0.200 | 4.874 | 0.072 | 4.07 | 4.25 | 4.55 | 4.87 | 5.20 | 5.50 | 5.68 |
| 13 | 62 | 5.11 | 0.40 | -0.200 | 5.114 | 0.072 | 4.27 | 4.46 | 4.77 | 5.11 | 5.46 | 5.77 | 5.96 |
| 14 | 60 | 5.43 | 0.45 | -0.200 | 5.383 | 0.072 | 4.50 | 4.69 | 5.02 | 5.38 | 5.75 | 6.07 | 6.27 |
| 15 | 47 | 5.69 | 0.27 | -0.200 | 5.674 | 0.072 | 4.74 | 4.95 | 5.29 | 5.67 | 6.06 | 6.40 | 6.61 |

**Table S35.** Age (years), number of individuals (N), mean, standard deviation (S.D.), L, M, and S values, and percentiles of the distal phalanx of the fifth digit according to bone age in males.

| **Age** | **N** | **Mean** | **S.D.** | **L** | **M** | **S** | **P5** | **P10** | **P25** | **P50** | **P75** | **P90** | **P95** |
| --- | --- | --- | --- | --- | --- | --- | --- | --- | --- | --- | --- | --- | --- |
| 6 | 29 | 1.07 | 0.08 | 0.494 | 1.061 | 0.093 | 0.89 | 0.92 | 0.99 | 1.06 | 1.13 | 1.20 | 1.24 |
| 7 | 28 | 1.12 | 0.12 | 0.494 | 1.099 | 0.093 | 0.92 | 0.96 | 1.02 | 1.10 | 1.17 | 1.24 | 1.28 |
| 8 | 40 | 1.13 | 0.11 | 0.494 | 1.140 | 0.093 | 0.95 | 0.99 | 1.06 | 1.14 | 1.22 | 1.29 | 1.33 |
| 9 | 40 | 1.19 | 0.12 | 0.494 | 1.185 | 0.093 | 0.99 | 1.03 | 1.11 | 1.19 | 1.27 | 1.34 | 1.38 |
| 10 | 53 | 1.24 | 0.12 | 0.494 | 1.237 | 0.093 | 1.03 | 1.08 | 1.15 | 1.24 | 1.32 | 1.40 | 1.44 |
| 11 | 56 | 1.29 | 0.12 | 0.494 | 1.296 | 0.093 | 1.08 | 1.13 | 1.21 | 1.30 | 1.38 | 1.46 | 1.51 |
| 12 | 30 | 1.36 | 0.11 | 0.494 | 1.363 | 0.093 | 1.14 | 1.19 | 1.27 | 1.36 | 1.45 | 1.54 | 1.59 |
| 13 | 62 | 1.45 | 0.14 | 0.494 | 1.438 | 0.093 | 1.20 | 1.25 | 1.34 | 1.44 | 1.54 | 1.62 | 1.67 |
| 14 | 60 | 1.54 | 0.14 | 0.494 | 1.519 | 0.093 | 1.27 | 1.32 | 1.42 | 1.52 | 1.62 | 1.71 | 1.77 |
| 15 | 47 | 1.60 | 0.15 | 0.494 | 1.604 | 0.093 | 1.34 | 1.40 | 1.50 | 1.60 | 1.71 | 1.81 | 1.87 |

**Table S36.** Age (years), number of individuals (N), mean, standard deviation (S.D.), L, M, and S values, and percentiles of the middle phalanx of the fifth digit according to bone age in males.

| **Age** | **N** | **Mean** | **S.D.** | **L** | **M** | **S** | **P5** | **P10** | **P25** | **P50** | **P75** | **P90** | **P95** |
| --- | --- | --- | --- | --- | --- | --- | --- | --- | --- | --- | --- | --- | --- |
| 6 | 29 | 1.19 | 0.13 | 0.923 | 1.204 | 0.106 | 1.01 | 1.05 | 1.12 | 1.20 | 1.29 | 1.36 | 1.40 |
| 7 | 28 | 1.28 | 0.17 | 0.923 | 1.243 | 0.106 | 1.04 | 1.08 | 1.16 | 1.24 | 1.33 | 1.40 | 1.45 |
| 8 | 40 | 1.27 | 0.11 | 0.923 | 1.282 | 0.106 | 1.07 | 1.12 | 1.20 | 1.28 | 1.37 | 1.45 | 1.49 |
| 9 | 40 | 1.34 | 0.13 | 0.923 | 1.323 | 0.106 | 1.11 | 1.15 | 1.23 | 1.32 | 1.41 | 1.49 | 1.54 |
| 10 | 53 | 1.36 | 0.17 | 0.923 | 1.370 | 0.106 | 1.14 | 1.19 | 1.28 | 1.37 | 1.46 | 1.55 | 1.60 |
| 11 | 56 | 1.42 | 0.17 | 0.923 | 1.426 | 0.106 | 1.19 | 1.24 | 1.33 | 1.43 | 1.52 | 1.61 | 1.66 |
| 12 | 30 | 1.47 | 0.17 | 0.923 | 1.496 | 0.106 | 1.25 | 1.30 | 1.39 | 1.50 | 1.60 | 1.69 | 1.74 |
| 13 | 62 | 1.58 | 0.16 | 0.923 | 1.580 | 0.106 | 1.32 | 1.38 | 1.47 | 1.58 | 1.69 | 1.78 | 1.84 |
| 14 | 60 | 1.70 | 0.17 | 0.923 | 1.678 | 0.106 | 1.40 | 1.46 | 1.56 | 1.68 | 1.79 | 1.89 | 1.95 |
| 15 | 47 | 1.80 | 0.12 | 0.923 | 1.785 | 0.106 | 1.49 | 1.56 | 1.66 | 1.79 | 1.91 | 2.01 | 2.08 |

**Table S37.** Age (years), number of individuals (N), mean, standard deviation (S.D.), L, M, and S values, and percentiles of the proximal phalanx of the fifth digit according to bone age in males.

| **Age** | **N** | **Mean** | **S.D.** | **L** | **M** | **S** | **P5** | **P10** | **P25** | **P50** | **P75** | **P90** | **P95** |
| --- | --- | --- | --- | --- | --- | --- | --- | --- | --- | --- | --- | --- | --- |
| 6 | 29 | 2.16 | 0.15 | -0.134 | 2.163 | 0.074 | 1.81 | 1.89 | 2.02 | 2.16 | 2.31 | 2.44 | 2.52 |
| 7 | 28 | 2.26 | 0.18 | -0.134 | 2.231 | 0.074 | 1.86 | 1.95 | 2.08 | 2.23 | 2.38 | 2.52 | 2.60 |
| 8 | 40 | 2.33 | 0.15 | -0.134 | 2.300 | 0.074 | 1.92 | 2.01 | 2.14 | 2.30 | 2.46 | 2.59 | 2.68 |
| 9 | 40 | 2.36 | 0.18 | -0.134 | 2.372 | 0.074 | 1.98 | 2.07 | 2.21 | 2.37 | 2.53 | 2.68 | 2.76 |
| 10 | 53 | 2.46 | 0.19 | -0.134 | 2.456 | 0.074 | 2.05 | 2.14 | 2.29 | 2.46 | 2.62 | 2.77 | 2.86 |
| 11 | 56 | 2.55 | 0.18 | -0.134 | 2.556 | 0.074 | 2.14 | 2.23 | 2.38 | 2.56 | 2.73 | 2.88 | 2.98 |
| 12 | 30 | 2.70 | 0.18 | -0.134 | 2.677 | 0.074 | 2.24 | 2.33 | 2.50 | 2.68 | 2.86 | 3.02 | 3.12 |
| 13 | 62 | 2.81 | 0.23 | -0.134 | 2.815 | 0.074 | 2.35 | 2.45 | 2.63 | 2.82 | 3.01 | 3.18 | 3.28 |
| 14 | 60 | 3.01 | 0.26 | -0.134 | 2.971 | 0.074 | 2.48 | 2.59 | 2.77 | 2.97 | 3.17 | 3.35 | 3.46 |
| 15 | 47 | 3.14 | 0.16 | -0.134 | 3.137 | 0.074 | 2.62 | 2.74 | 2.93 | 3.14 | 3.35 | 3.54 | 3.65 |

**Table S38.** Age (years), number of individuals (N), mean, standard deviation (S.D.), L, M, and S values, and percentiles of the fifth metacarpal according to bone age in males.

| **Age** | **N** | **Mean** | **S.D.** | **L** | **M** | **S** | **P5** | **P10** | **P25** | **P50** | **P75** | **P90** | **P95** |
| --- | --- | --- | --- | --- | --- | --- | --- | --- | --- | --- | --- | --- | --- |
| 6 | 29 | 3.53 | 0.24 | -0.554 | 3.543 | 0.069 | 2.96 | 3.09 | 3.30 | 3.54 | 3.78 | 4.00 | 4.13 |
| 7 | 28 | 3.71 | 0.26 | -0.554 | 3.683 | 0.069 | 3.08 | 3.21 | 3.43 | 3.68 | 3.93 | 4.15 | 4.29 |
| 8 | 40 | 3.90 | 0.25 | -0.554 | 3.822 | 0.069 | 3.19 | 3.33 | 3.56 | 3.82 | 4.08 | 4.31 | 4.45 |
| 9 | 40 | 3.94 | 0.34 | -0.554 | 3.962 | 0.069 | 3.31 | 3.45 | 3.70 | 3.96 | 4.23 | 4.47 | 4.61 |
| 10 | 53 | 4.14 | 0.32 | -0.554 | 4.117 | 0.069 | 3.44 | 3.59 | 3.84 | 4.12 | 4.39 | 4.64 | 4.79 |
| 11 | 56 | 4.29 | 0.24 | -0.554 | 4.294 | 0.069 | 3.59 | 3.74 | 4.00 | 4.29 | 4.58 | 4.84 | 5.00 |
| 12 | 30 | 4.51 | 0.28 | -0.554 | 4.499 | 0.069 | 3.76 | 3.92 | 4.20 | 4.50 | 4.80 | 5.08 | 5.24 |
| 13 | 62 | 4.74 | 0.36 | -0.554 | 4.735 | 0.069 | 3.96 | 4.13 | 4.42 | 4.73 | 5.05 | 5.34 | 5.51 |
| 14 | 60 | 5.04 | 0.39 | -0.554 | 4.999 | 0.069 | 4.18 | 4.36 | 4.66 | 5.00 | 5.34 | 5.64 | 5.82 |
| 15 | 47 | 5.30 | 0.24 | -0.554 | 5.286 | 0.069 | 4.42 | 4.61 | 4.93 | 5.29 | 5.64 | 5.96 | 6.16 |

**Figures**

**Female chronological age**


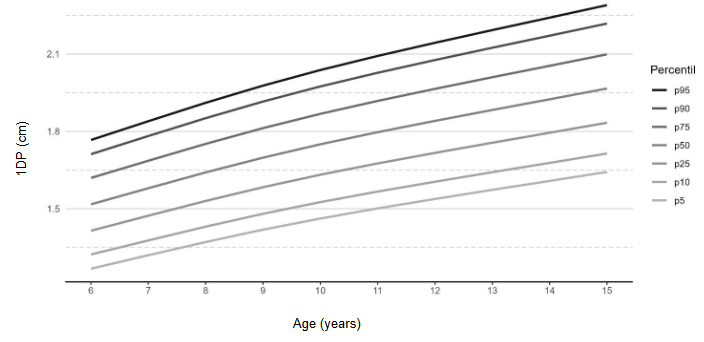


**Figure S1.** Percentile curves of the length of the distal phalanx of the first digit in females according to chronological age.


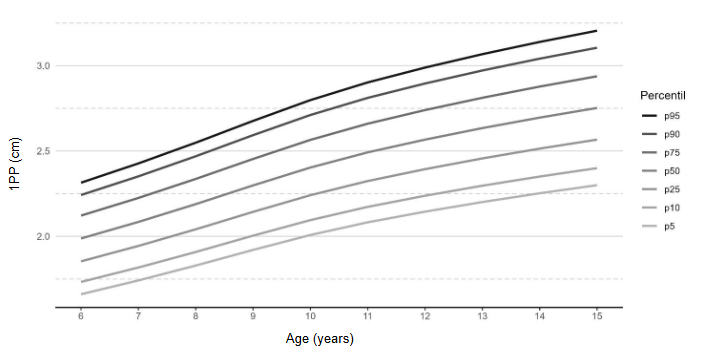


**Figure S2.** Percentile curves of the length of the proximal phalanx of the first digit in females according to chronological age.


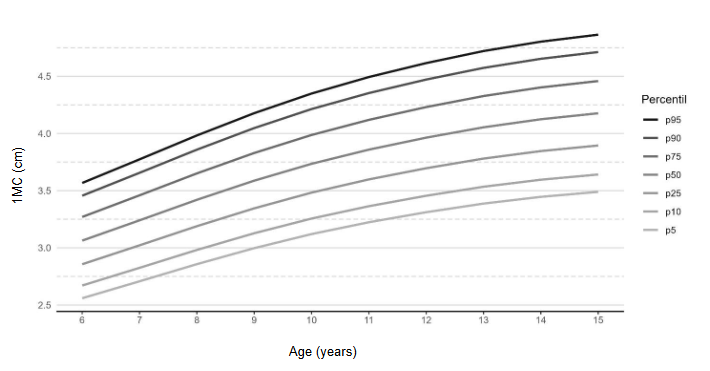


**Figure S3.** Percentile curves of the length of the first metacarpal in females according to chronological age.


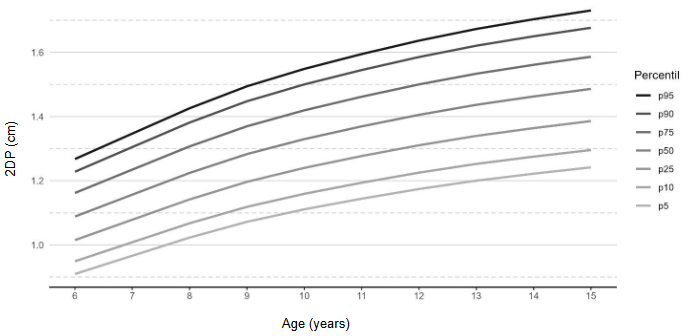


**Figure S4.** Percentile curves of the length of the distal phalanx of the second digit in females according to chronological age.


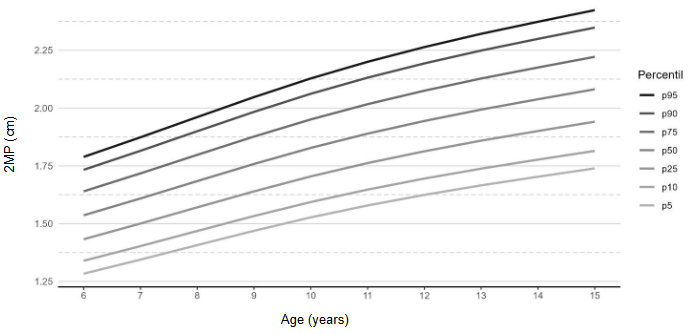


**Figure S5.** Percentile curves of the length of the middle phalanx of the second digit in females according to chronological age.


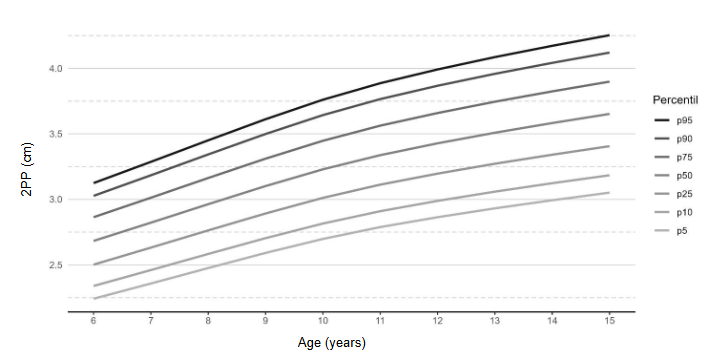


**Figure S6.** Percentile curves of the length of the proximal phalanx of the second digit in females according to chronological age.


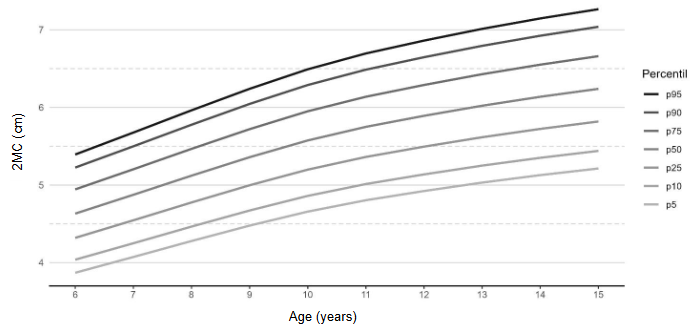


**Figure S7.** Percentile curves of the length of the second metacarpal in females according to chronological age.


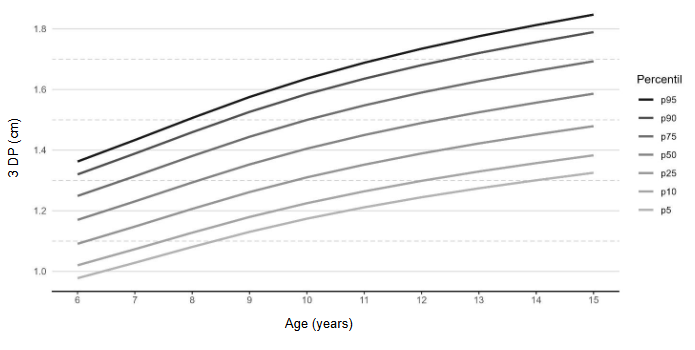


**Figure S8.** Percentile curves of the length of the distal phalanx of the third digit in females according to chronological age.


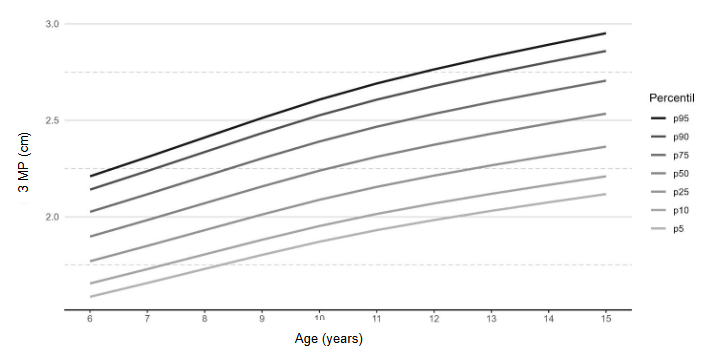


**Figure S9.** Percentile curves of the length of the middle phalanx of the third digit in females according to chronological age.


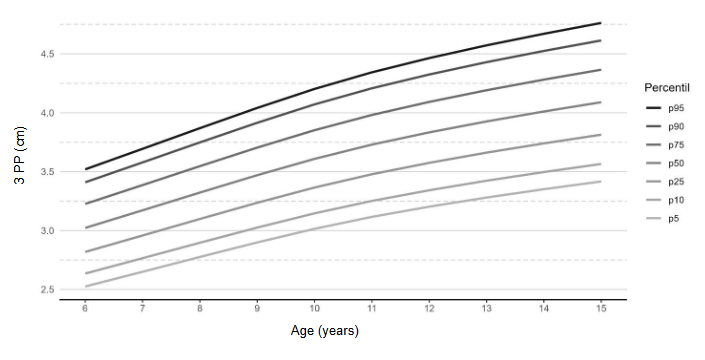


**Figure S10.** Percentile curves of the length of the proximal phalanx of the third digit in females according to chronological age.


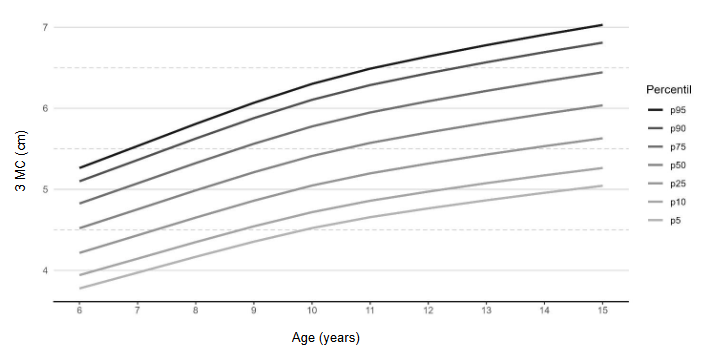


**Figure S11.** Percentile curves of the length of the third metacarpal in females according to chronological age.


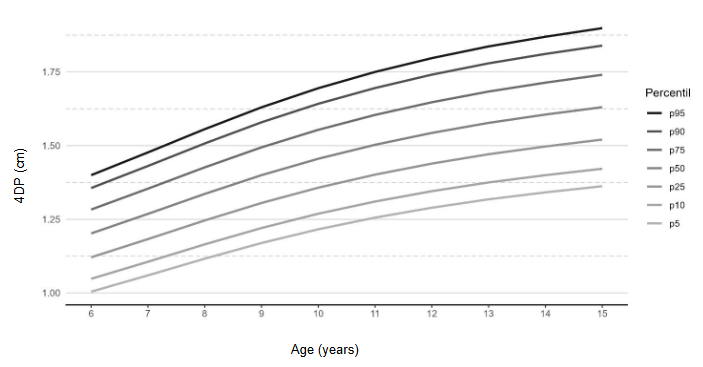


**Figure S12.** Percentile curves of the length of the distal phalanx of the fourth digit in females according to chronological age.


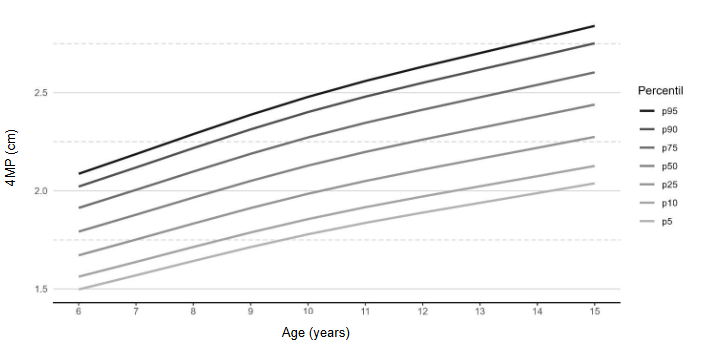


**Figure S13.** Percentile curves of the length of the middle phalanx of the fourth digit in females according to chronological age.


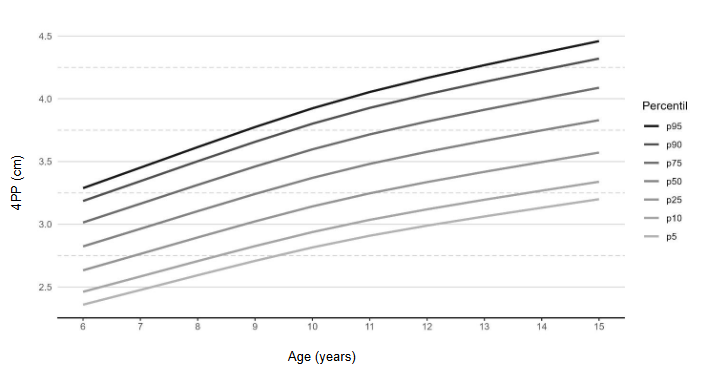


**Figure S14.** Percentile curves of the length of the proximal phalanx of the fourth digit in females according to chronological age.


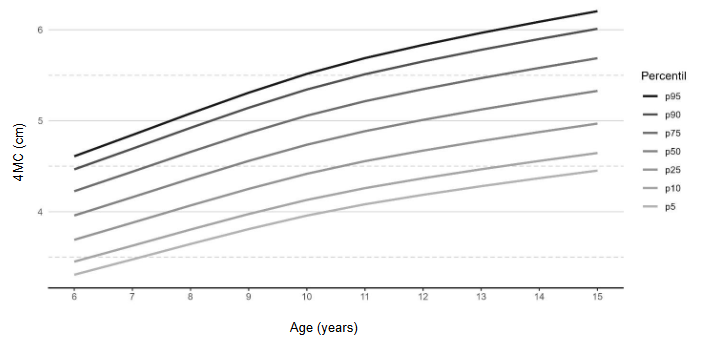


**Figure S15.** Percentile curves of the length of the fourth metacarpal in females according to chronological age.


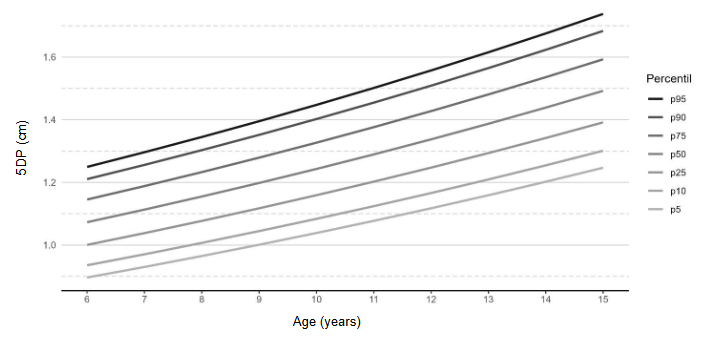


**Figure S16.** Percentile curves of the length of the distal phalanx of the fifth digit in females according to chronological age.


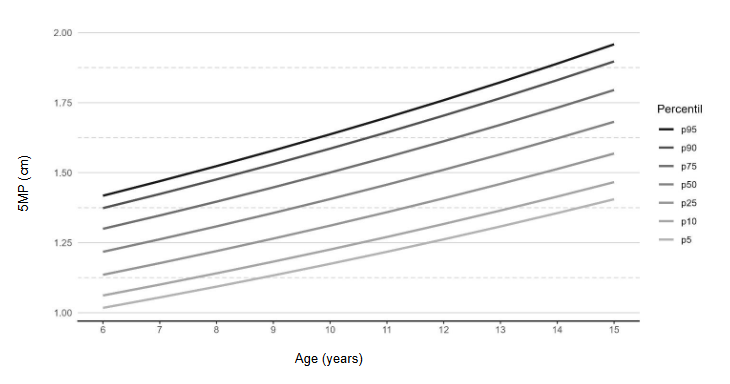


**Figure S17.** Percentile curves of the length of the middle phalanx of the fifth digit in females according to chronological age.


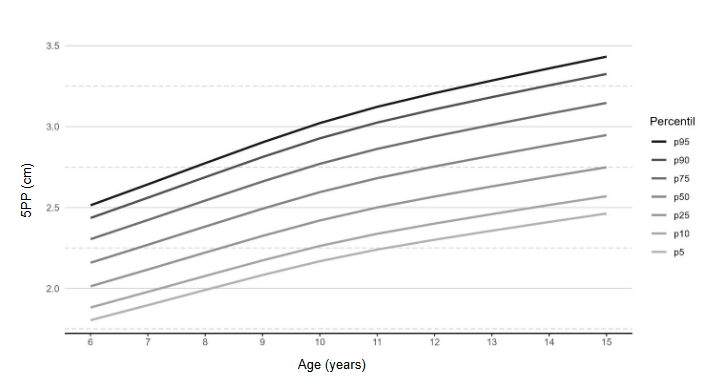


**Figure S18.** Percentile curves of the length of the proximal phalanx of the fifth digit in females according to chronological age.


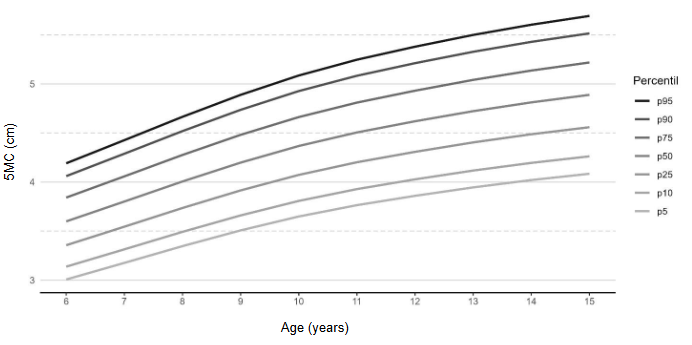


**Figure S19.** Percentile curves of the length of the fifth metacarpal in females according to chronological age.

**Male chronological age**


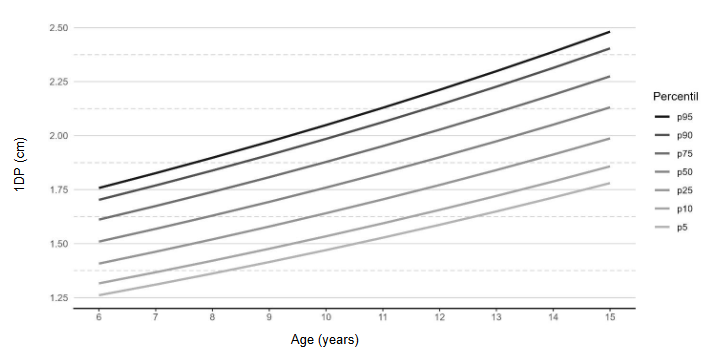


**Figure S20.** Percentile curves of the length of the distal phalanx of the first digit in males according to chronological age.


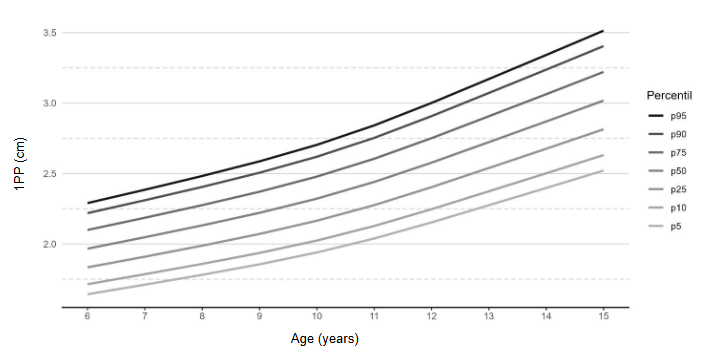


**Figure S21.** Percentile curves of the length of the proximal phalanx of the first digit in males according to chronological age.


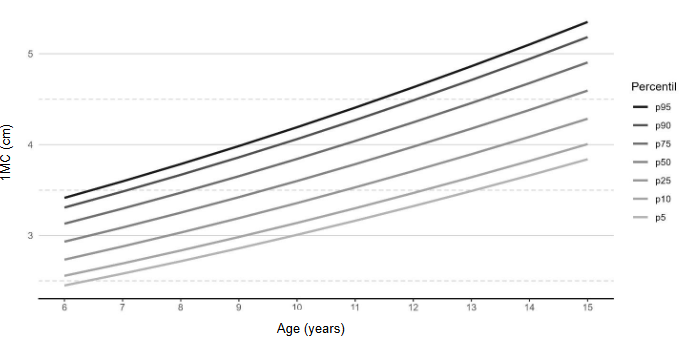


**Figure S22.** Percentile curves of the length of the first metacarpal in males according to chronological age.


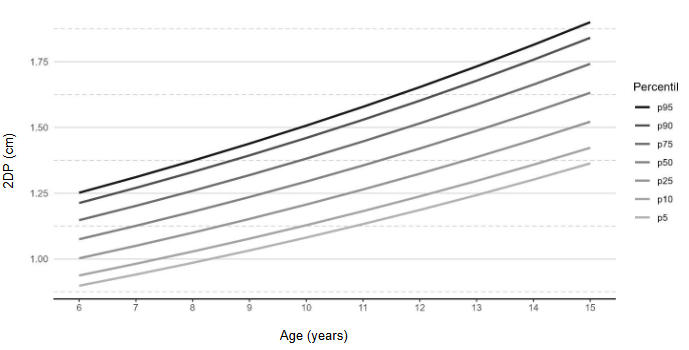


**Figure S23.** Percentile curves of the length of the distal phalanx of the second digit in males according to chronological age.


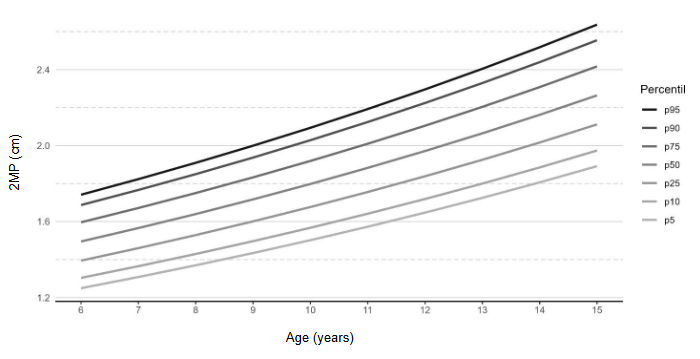


**Figure S24.** Percentile curves of the length of the middle phalanx of the second digit in males according to chronological age.


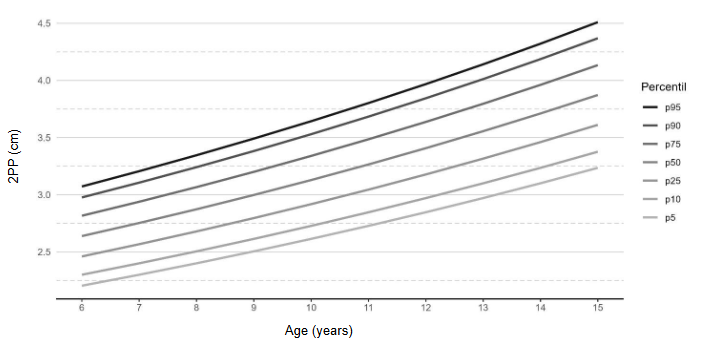


**Figure S25.** Percentile curves of the length of the proximal phalanx of the second digit in males according to chronological age.


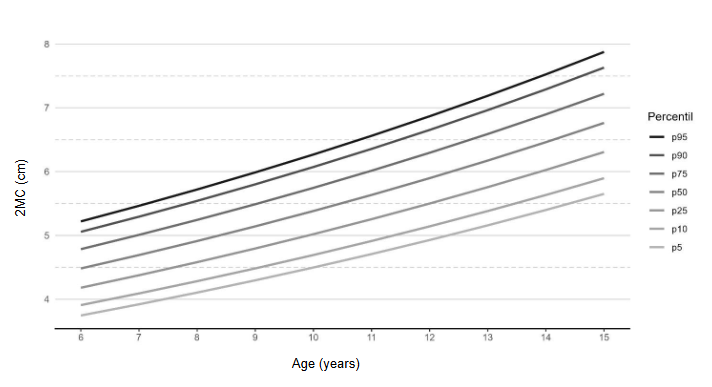


**Figure S26.** Percentile curves of the length of the second metacarpal in males according to chronological age.


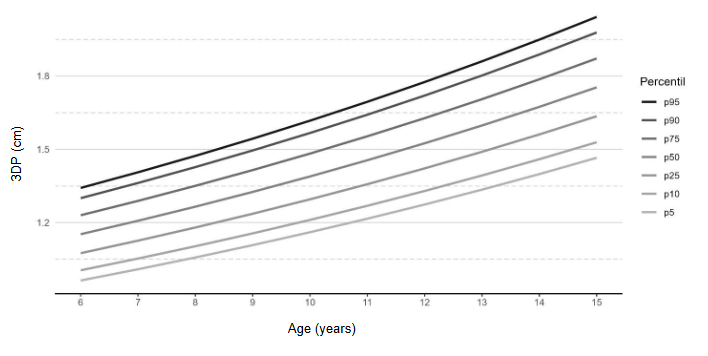


**Figure S27.** Percentile curves of the length of the distal phalanx of the third digit in males according to chronological age.


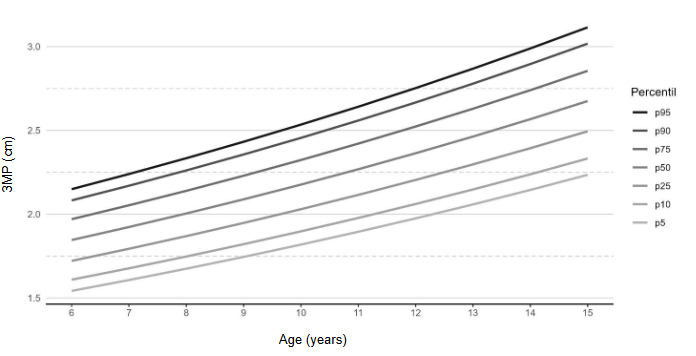


**Figure S28.** Percentile curves of the length of the middle phalanx of the third digit in males according to chronological age.


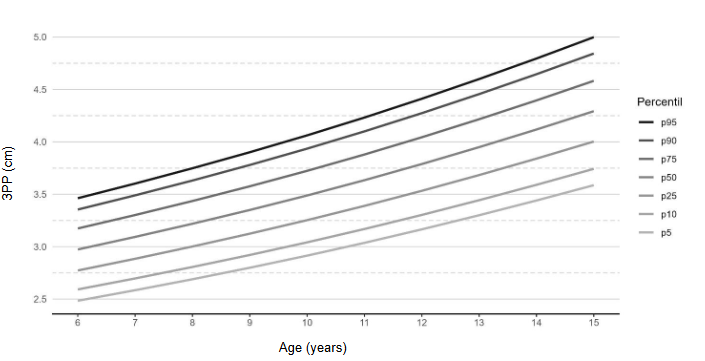


**Figure S29.** Percentile curves of the length of the proximal phalanx of the third digit in males according to chronological age.


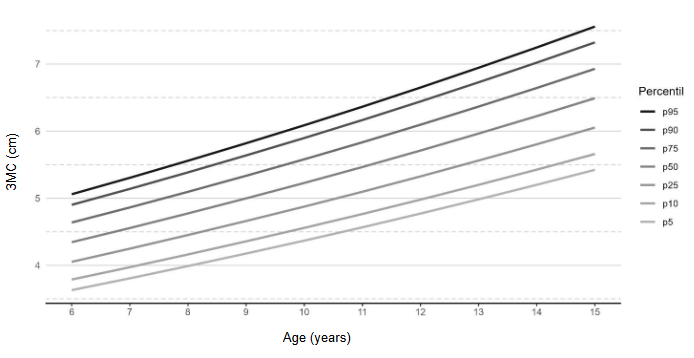


**Figure S30.** Percentile curves of the length of the third metacarpal in males according to chronological age.


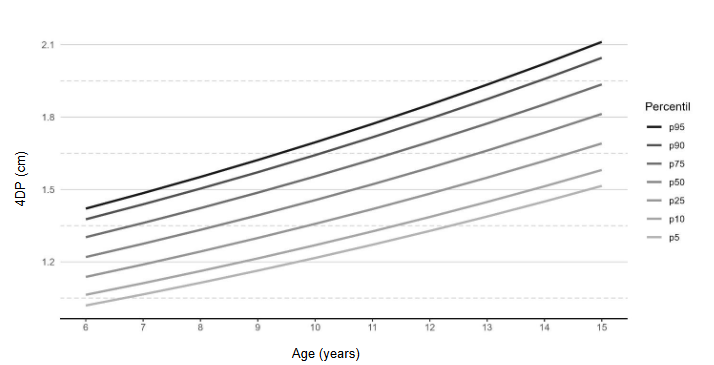


**Figure S31.** Percentile curves of the length of the distal phalanx of the fourth digit in males according to chronological age.


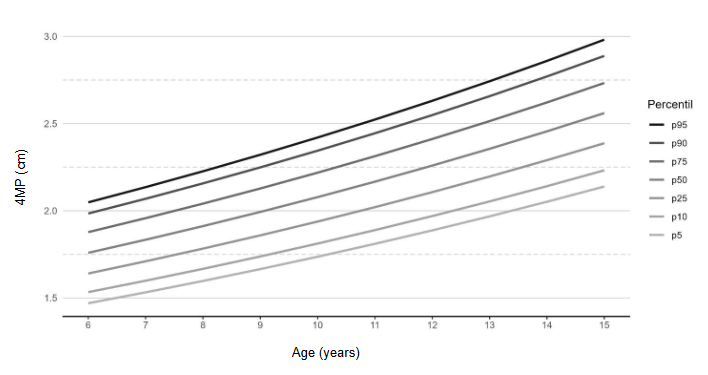


**Figure S32.** Percentile curves of the length of the middle phalanx of the fourth digit in males according to chronological age.


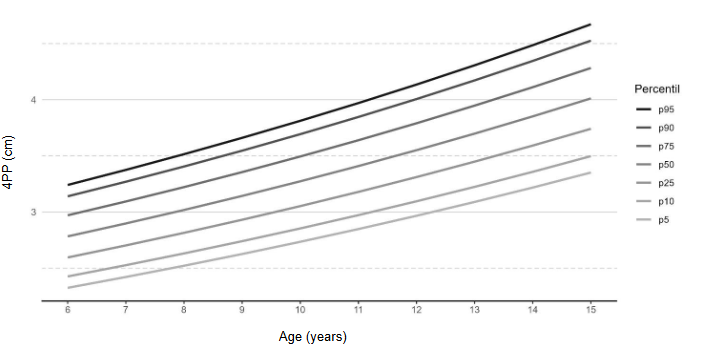


**Figure S33.** Percentile curves of the length of the proximal phalanx of the fourth digit in males according to chronological age.


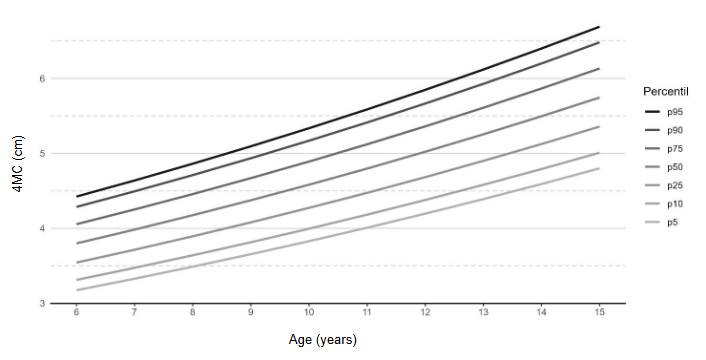


**Figure S34.** Percentile curves of the length of the fourth metacarpal in males according to chronological age.


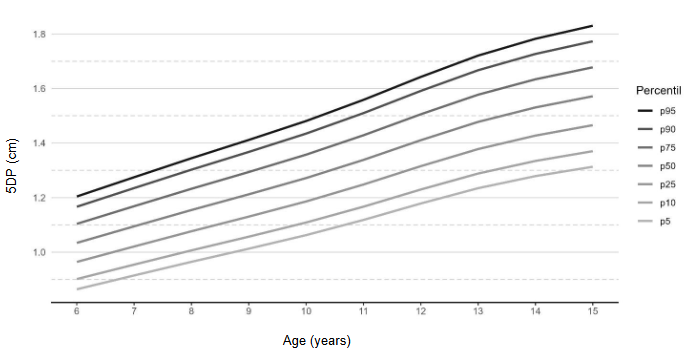


**Figure S35.** Percentile curves of the length of the distal phalanx of the fifth digit in males according to chronological age.


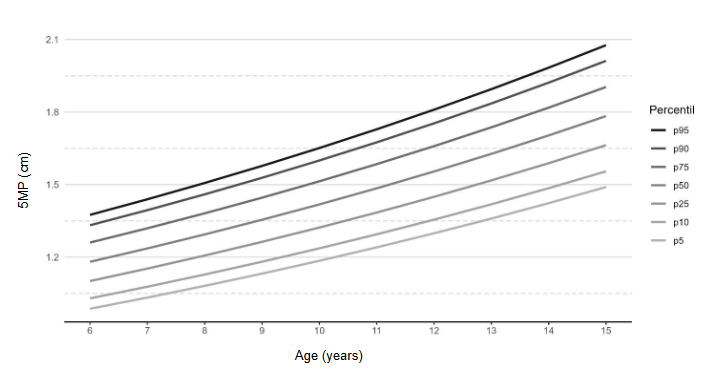


**Figure S36.** Percentile curves of the length of the middle phalanx of the fifth digit in males according to chronological age.


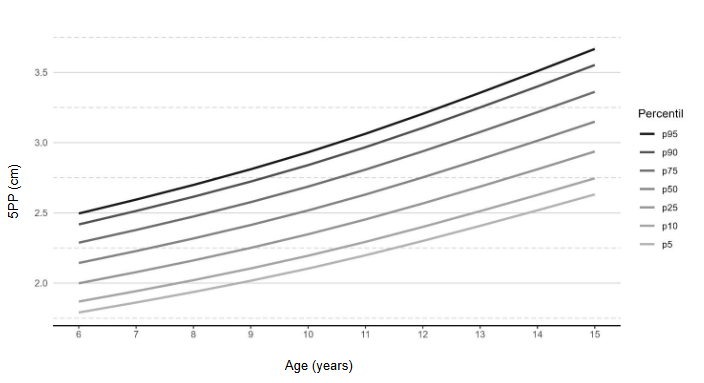


**Figure S37.** Percentile curves of the length of the proximal phalanx of the fifth digit in males according to chronological age.


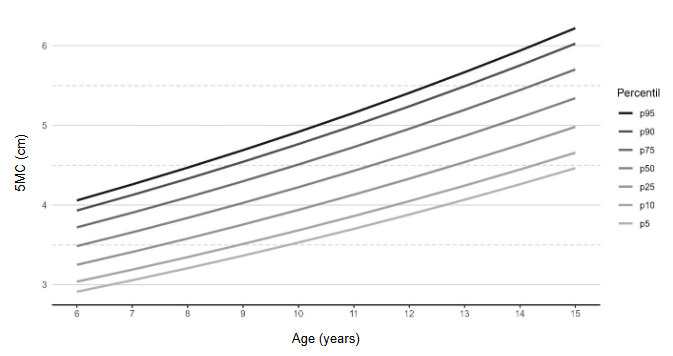


**Figure S38.** Percentile curves of the length of the fifth metacarpal in males according to chronological age.

**Female – Bone Age**


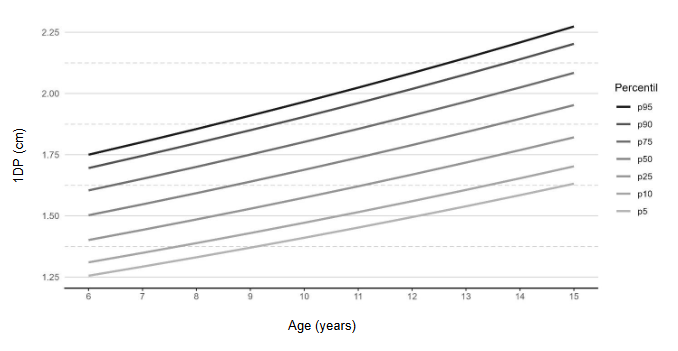


**Figure S39.** Percentile curves of the length of the distal phalanx of the first digit in females according to bone age.


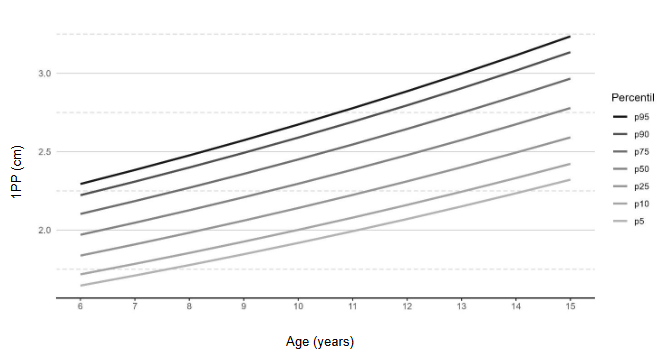


**Figure S40.** Percentile curves of the length of the proximal phalanx of the first digit in females according to bone age.


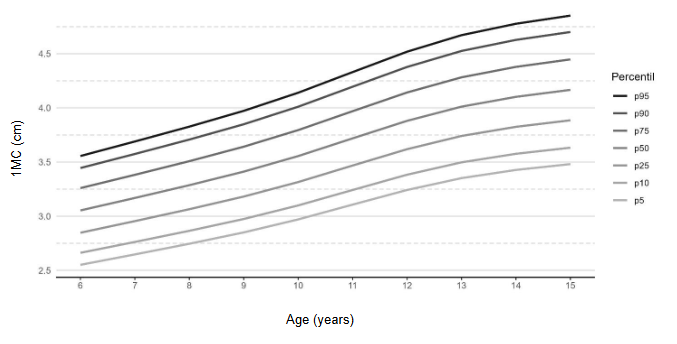


**Figure S41.** Percentile curves of the length of the first metacarpal in females according to bone age.


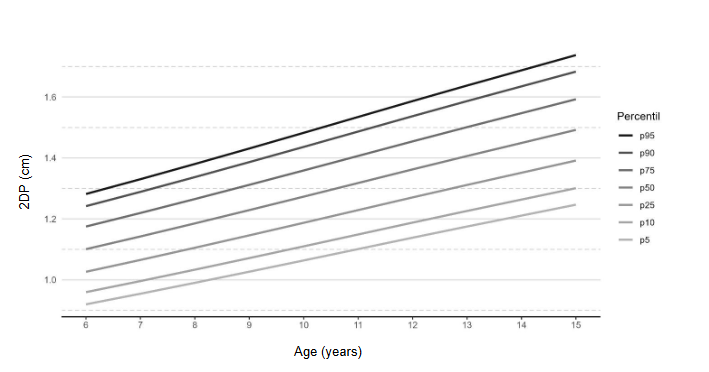


**Figure S42.** Percentile curves of the length of the distal phalanx of the second digit in females according to bone age.


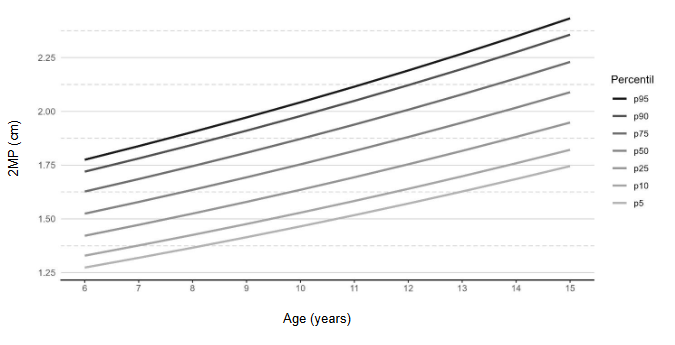


**Figure S43.** Percentile curves of the length of the middle phalanx of the second digit in females according to bone age.


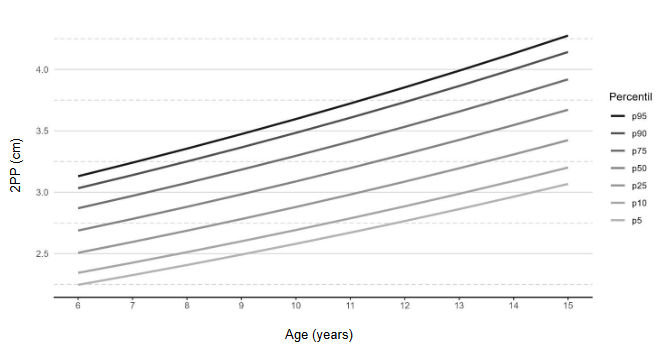


**Figure S44.** Percentile curves of the length of the proximal phalanx of the second digit in females according to bone age.


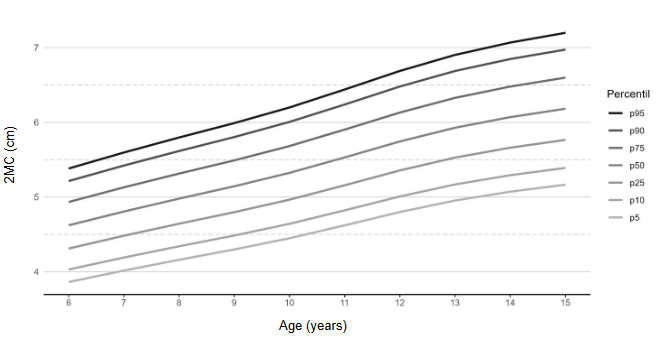


**Figure S45.** Percentile curves of the length of the second metacarpal in females according to bone age.


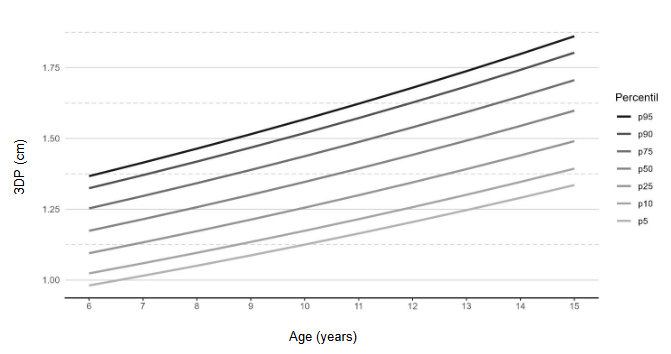


**Figure S46.** Percentile curves of the length of the distal phalanx of the third digit in females according to bone age.


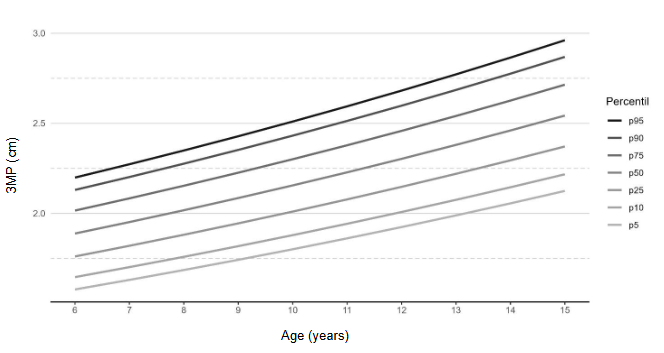


**Figure S47.** Percentile curves of the length of the middle phalanx of the third digit in females according to bone age.


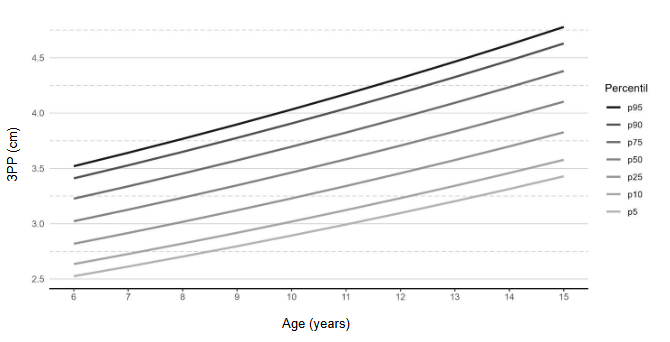


**Figure S48.** Percentile curves of the length of the proximal phalanx of the third digit in females according to bone age.


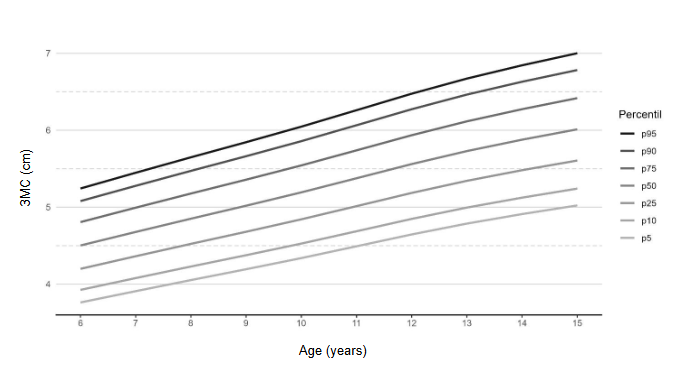


**Figure S49.** Percentile curves of the length of the third metacarpal in females according to bone age.


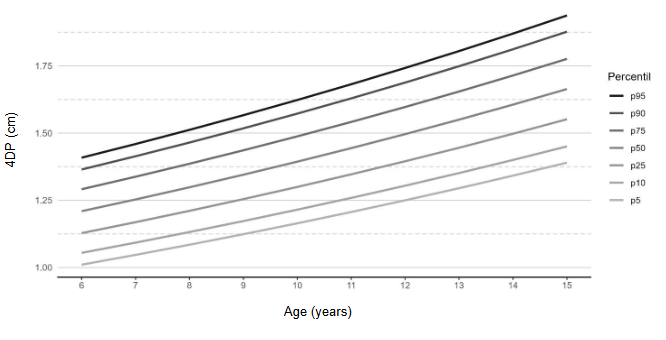


**Figure S50.** Percentile curves of the length of the distal phalanx of the fourth digit in females according to bone age.


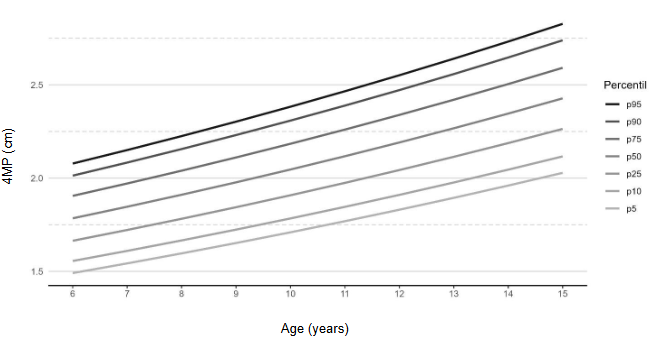


**Figure S51.** Percentile curves of the length of the middle phalanx of the fourth digit in females according to bone age.


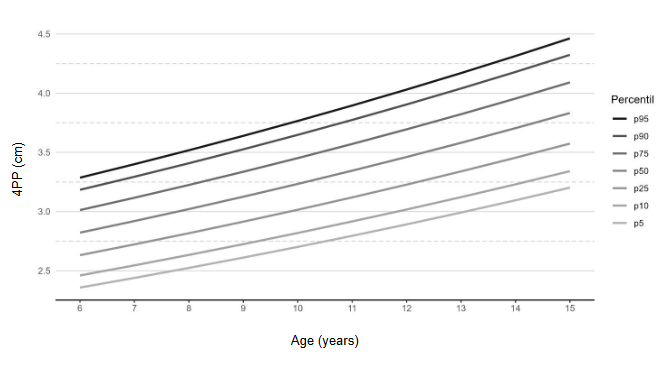


**Figure S52.** Percentile curves of the length of the proximal phalanx of the fourth digit in females according to bone age.


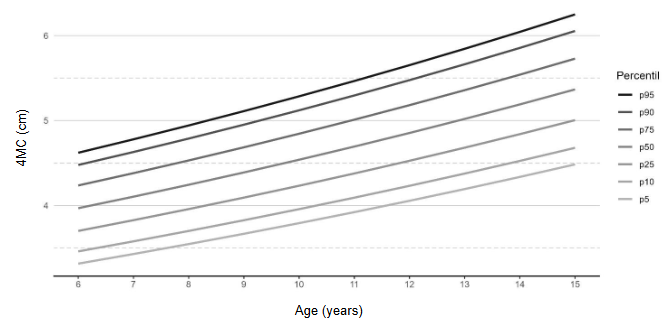


**Figure S53.** Percentile curves of the length of the fourth metacarpal in females according to bone age.


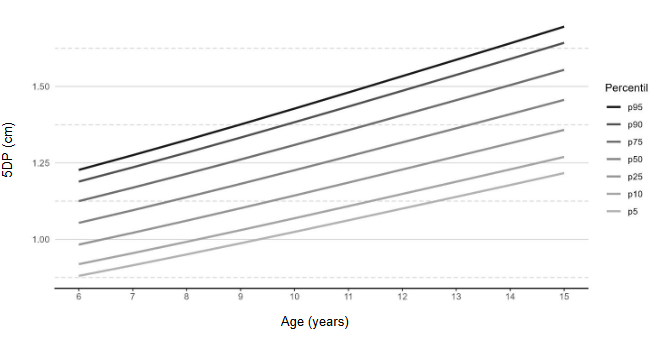


**Figure S54.** Percentile curves of the length of the distal phalanx of the fifth digit in females according to bone age.


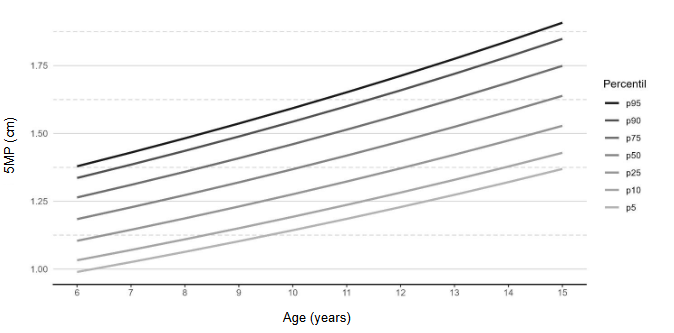


**Figure S55.** Percentile curves of the length of the middle phalanx of the fifth digit in females according to bone age.


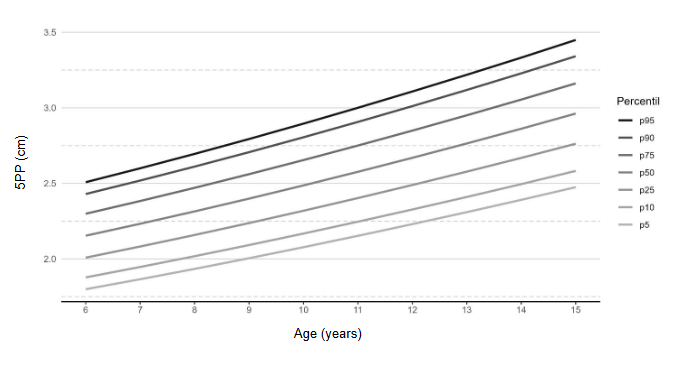


**Figure S56.** Percentile curves of the length of the proximal phalanx of the fifth digit in females according to bone age.


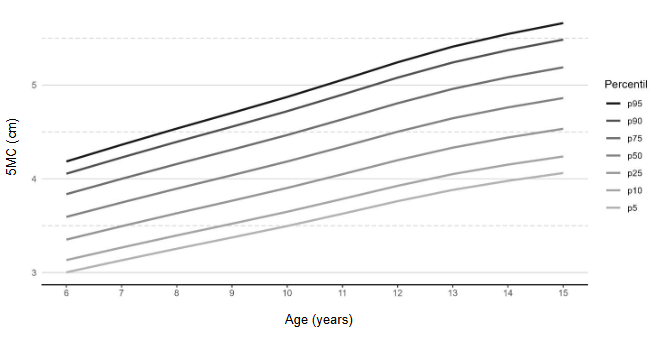


**Figure S57.** Percentile curves of the length of the fifth metacarpal in females according to bone age.

**Male – Bone Age**


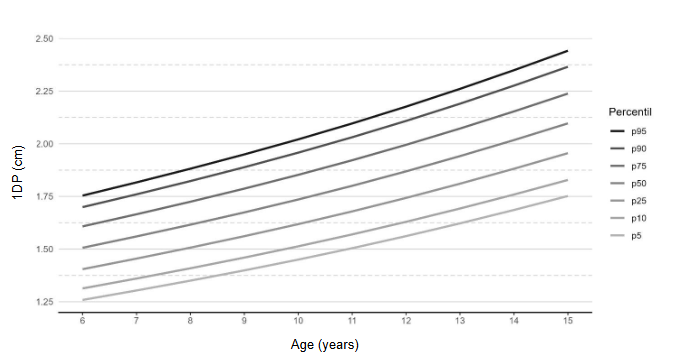


**Figure S58.** Percentile curves of the length of the distal phalanx of the first digit in males according to bone age.


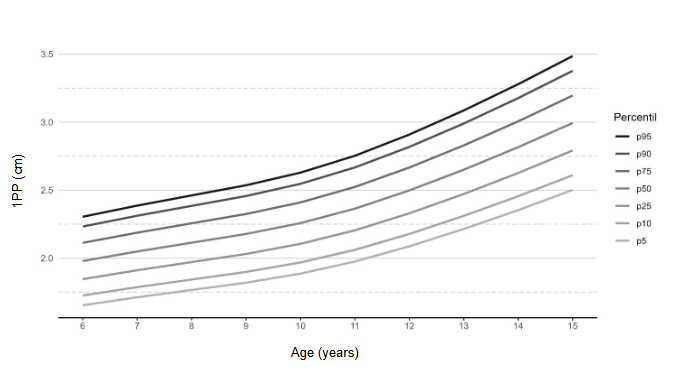


**Figure S59.** Percentile curves of the length of the proximal phalanx of the first digit in males according to bone age.


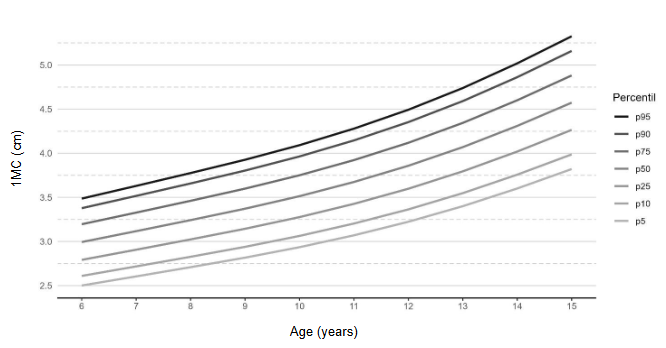


**Figure S60.** Percentile curves of the length of the first metacarpal in males according to bone age.


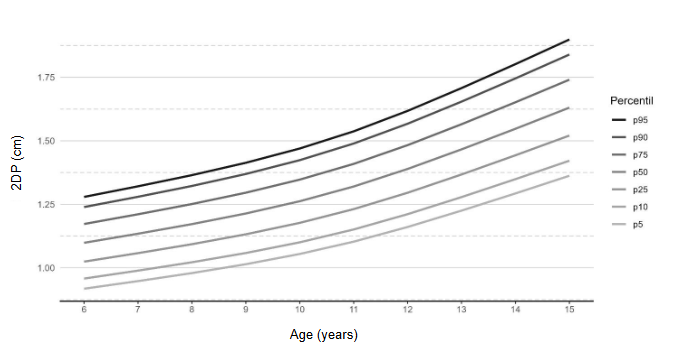


**Figure S61.** Percentile curves of the length of the distal phalanx of the second digit in males according to bone age.


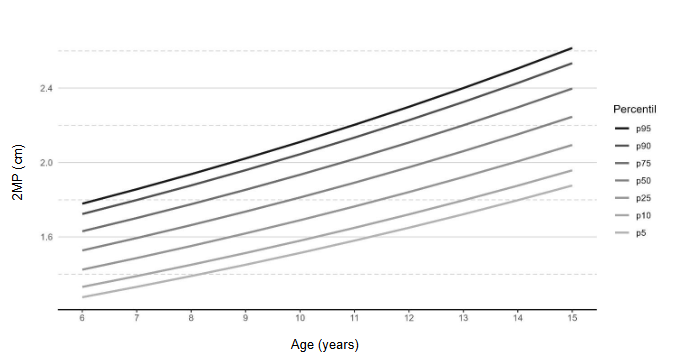


**Figure S62.** Percentile curves of the length of the middle phalanx of the second digit in males according to bone age.


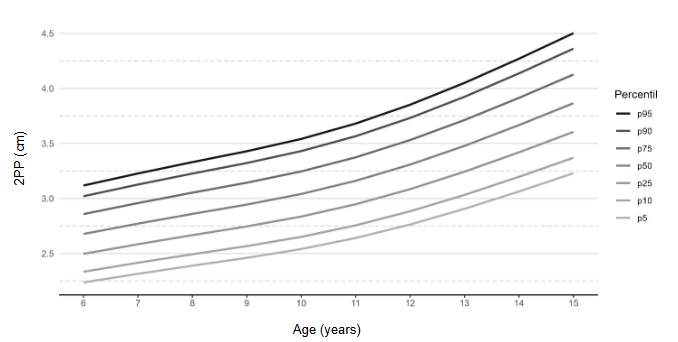


**Figure S63.** Percentile curves of the length of the proximal phalanx of the second digit in males according to bone age.


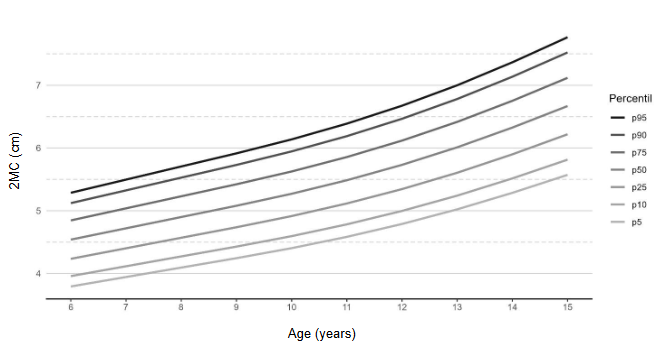


**Figure S64.** Percentile curves of the length of the second metacarpal in males according to bone age.


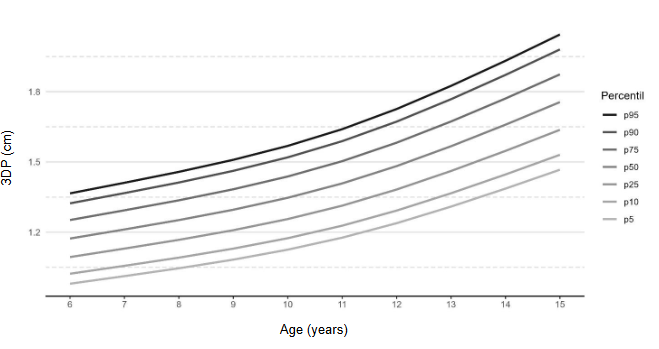


**Figure S65.** Percentile curves of the length of the distal phalanx of the third digit in males according to bone age.


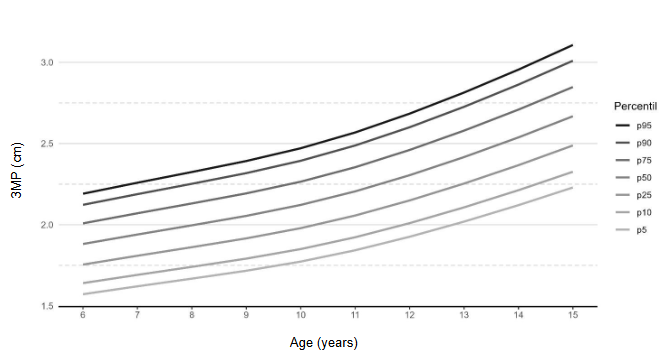


**Figure S66.** Percentile curves of the length of the middle phalanx of the third digit in males according to bone age.


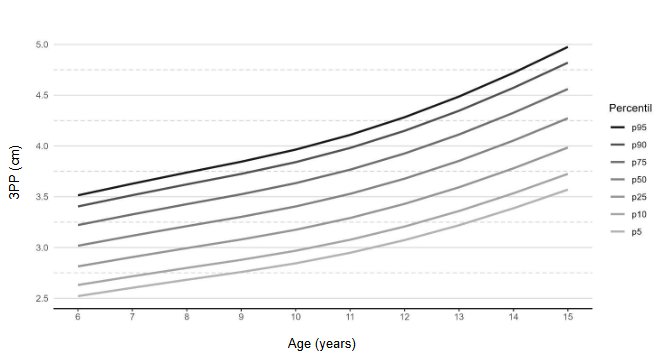


**Figure S67.** Percentile curves of the length of the proximal phalanx of the third digit in males according to bone age.


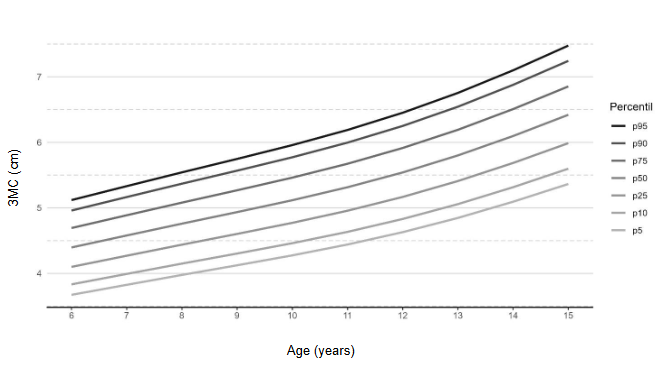


**Figure S68.** Percentile curves of the length of the third metacarpal in males according to bone age.


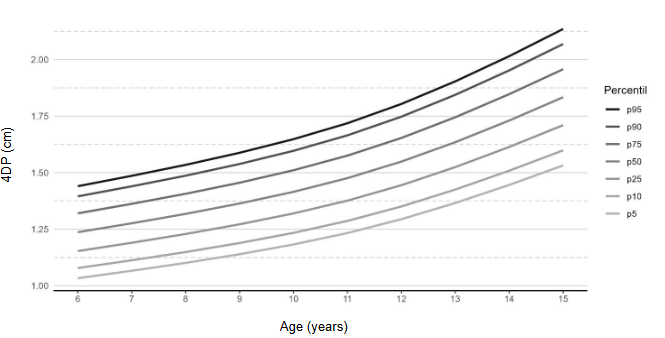


**Figure S69.** Percentile curves of the length of the distal phalanx of the fourth digit in males according to bone age.


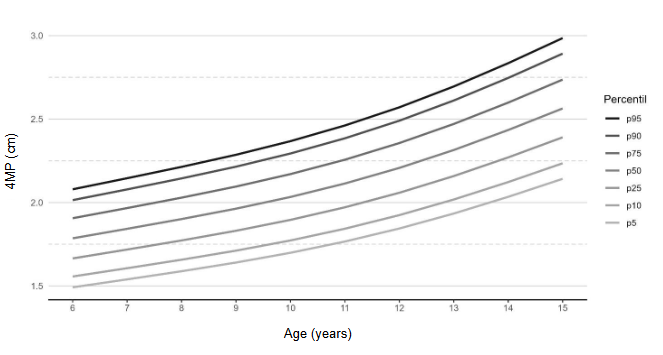


**Figure S70.** Percentile curves of the length of the middle phalanx of the fourth digit in males according to bone age.


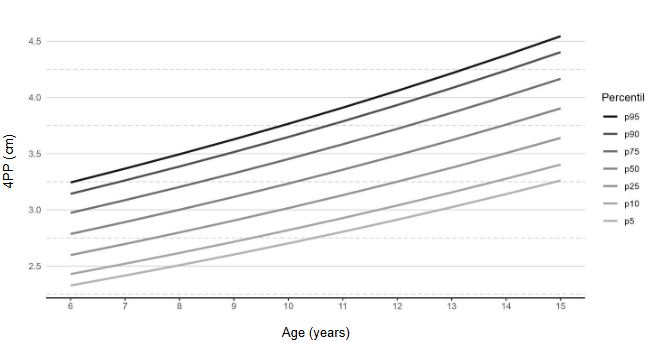


**Figure S71.** Percentile curves of the length of the proximal phalanx of the fourth digit in males according to bone age.


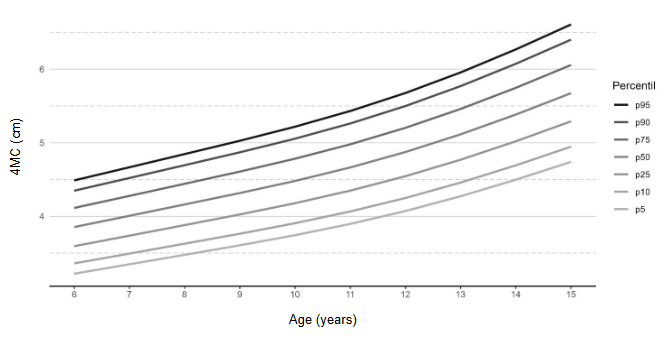


**Figure S72.** Percentile curves of the length of the fourth metacarpal in males according to bone age.


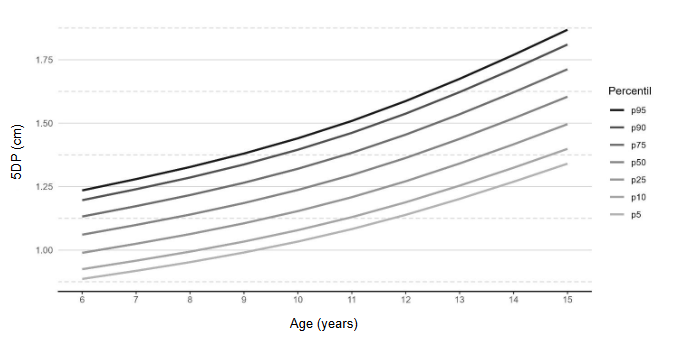


**Figure S73.** Percentile curves of the length of the distal phalanx of the fifth digit in males according to bone age.


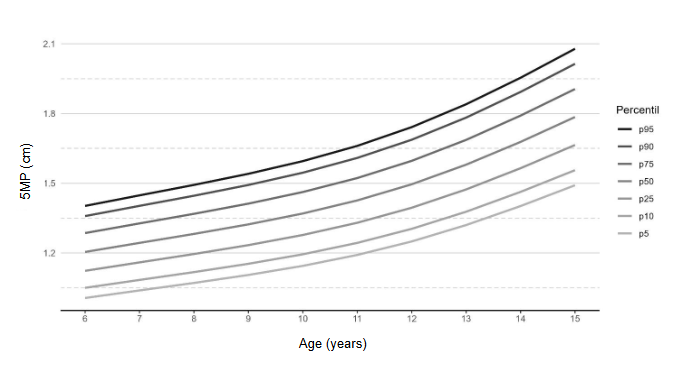


**Figure S74.** Percentile curves of the length of the middle phalanx of the fifth digit in males according to bone age.


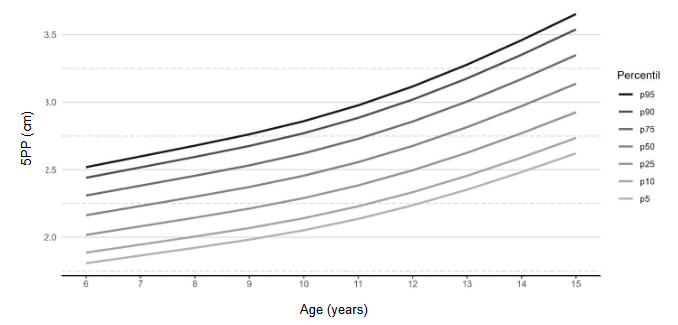


**Figure S75.** Percentile curves of the length of the proximal phalanx of the fifth digit in males according to bone age.


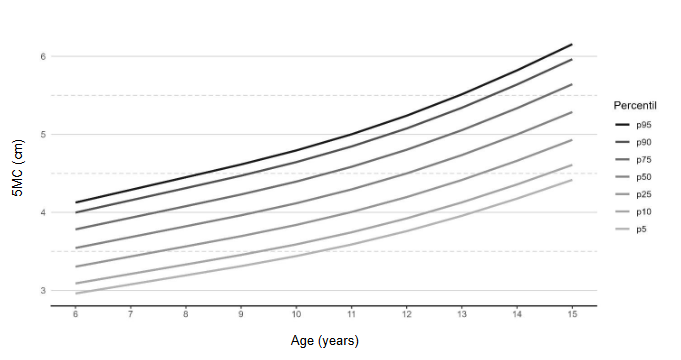


**Figure S76.** Percentile curves of the length of the fifth metacarpal in males according to bone age.
